# RAMS11 promotes CRC through mTOR-dependent inhibition of autophagy, suppression of apoptosis, and promotion of epithelial-mesenchymal transition

**DOI:** 10.1186/s12935-021-02023-6

**Published:** 2021-06-26

**Authors:** Md Zahirul Islam Khan, Helen Ka Wai Law

**Affiliations:** Department of Health Technology and Informatics, Faculty of Health and Social Sciences, The Hong Kong Polytechnic University, Hung Hom, Hong Kong, China

**Keywords:** LncRNAs, RAMS11, CRC, Autophagy, Apoptosis, EMT

## Abstract

**Background:**

Long non-coding RNAs (lncRNAs), a class of non-coding RNAs (ncRNAs) associated with diverse biological processes of cells. Over the past decades, cumulating research evidences revealed that abnormal expressions of lncRNAs are associated with colorectal cancer (CRC) initiation, progression, metastasis, and resistance to therapies. Moreover, their usefulness as candidate biomarkers for CRC diagnosis and prognosis are well evident throughout previous literature. In the current study, we examined the role and molecular mechanisms of newly identified lncRNA named RNA associated with metastasis-11 (RAMS11) in CRC development.

**Methods:**

The expression of RAMS11 in CRC cell lines DLD-1, HT-29, HCT-116, and SW480 and colon normal cells CCD-112-CoN were evaluated by quantitative RT-qPCR. The results showed that the RAMS11 is significantly upregulated in CRC cell lines compared to the normal cells. The CCK-8 proliferation assay, colony formation assay, and migration assay were performed to evaluate the biological and physiological functions of RAMS11 in vitro. To decipher the molecular mechanisms of RAMS11 medicated CRC progression, we further performed western blot analysis of the key pathway proteins (e.g., AMPK, AKT, and mTOR).

**Results:**

Our results revealed that higher expression of RAMS11 is associated with increased CRC proliferation, migration, and development of metastasis. Knockdown of RAMS11 induced autophagy, apoptosis along with reduction of epithelial-mesenchymal transition (EMT) suggesting that RAMS11 is involved in CRC progression. The molecular mechanisms of RAMS11 indicated that knockdown of RAMS11 significantly inhibited CRC carcinogenesis through mTOR-dependent autophagy induction.

**Conclusions:**

In sum, our results suggested that RAMS11 is an important oncogene in CRC pathogenesis. Targeting RAMS11 could be a potential therapeutic strategy for CRC management.

**Supplementary Information:**

The online version contains supplementary material available at 10.1186/s12935-021-02023-6.

## Background

Colorectal cancer (CRC) has been ranked as the third most diagnosed malignancy and the second leading cause of cancer-related death by The Global Cancer Incidence, Mortality and Prevalence (GLOBOCAN) statistics in 2018 [1, 2]. For the last few decades, the incidence and mortality was higher in western countries. However, the number of new cases and death is increasing in developing countries as well as East/Southeast Asia [3]. The formation of CRC is triggered by a sequences of genetic or epigenetic mutation of certain epithelial cells that acquired some selective advantages on their functional roles [4]. The abnormal cells then start to grow excessively and proliferate to form a benign adenoma which matured and turned into carcinoma, and lattermost metastasized to reach distant part of the body through the bloodstream [5]. Most of the CRC is diagnosed at age over 50 years and high mortality is mostly due to the development of metastasis [1, 3]. Globally, 20% of the patients diagnosed with CRC already had tumour metastasized to distant part of their body [1, 6]. Therefore, the search for a potential biomarkers and therapeutic targets for CRC diagnosis and prognosis may be useful for identifying individuals at risk of developing CRC or to hasten the diagnosis of early CRC for better treatment outcome.

Long non-coding RNAs (lncRNAs) are fragments of RNA, containing more than 200 nucleotides and routinely transcribed by RNA-polymerase-II in the human genome [7, 8]. LncRNAs have emerged to be a new aspect of cancer research. Previous research evidences suggested that their expressions are highly associated with specific cell-types and contributed to various cellular processes [7, 9]. It is noted that lncRNAs bind with DNA or RNA or proteins and play gene mediatory roles by promoting or inhibiting the transcription process [10]. Many studies also suggested that abnormal lncRNAs expressions contributed to CRC carcinogenesis through a cascade of regulatory signalling pathways such as autophagy, apoptosis, AMP activated protein kinase (AMPK), epithelial-mesenchymal transition (EMT), mTOR, PI3K/AKT, Wnt/β-catenin, JAK/STAT, MAPK, p53, and Notch [11–15]. In addition, the expression of lncRNAs may be used to monitor CRC progression and may practicable as diagnostic or therapeutic targets [15–17]. Therefore, exploring the epigenetic modification of lncRNAs associated with CRC growth and metastasis could open a new window for CRC diagnosis, prognosis and therapeutic targets.

RNA associated with metastasis-11 (RAMS11) is a newly identified lncRNA which was firstly identified by Dr. Maher’s Lab [18]. Using meta-analysis, they discovered that RAMS11 is highly upregulated in metastatic CRC and associated with reduced disease-free survival. In addition, the in vitro results indicated that upregulation of RAMS11 promoted aggressive CRC phenotypes by increasing proliferation, migration, invasion, and number of colonies in CRC cells. Furthermore, RAMS11 knockout reduced CRC growth and metastasis in vivo. Although their study has reported the role of RAMS11 in CRC carcinogenesis, they did not explored the molecular mechanisms such as autophagy. In this study, we aimed to explore the RAMS11 expression in CRC cell lines and the in-depth mechanism associated with carcinogenesis. We are novel in exploring the molecular mechanisms and demonstrated that silencing of RAMS11 may be used for personalized CRC management.

## Methods

### Cell lines and culture conditions

Human normal colon cell, CCD-112CoN, was acquired from American Type Culture Collection (ATCC), (Manassas, VA, USA) and human CRC HT-29-Red-Fluc cell was acquired from PerkinElmer, Inc. (Waltham, USA). In addition, three more human CRC cell lines, namely DLD-1, HCT-116 and SW480 were kindly provided by Professor Jun YU, Department of Medicine and Therapeutics, Institute of Digestive Diseases, The Chinese University of Hong Kong. The growth condition of CCD-112CoN cells were maintained with 10% fetal bovine serum (FBS), (Gibco, USA) in Eagle’s minimum essential medium (EMEM, ATCC, Manassas, VA). Whereas, HT-29, DLD-1, HCT-116 and SW480 were cultured in Dulbecco’s modified eagle medium (DMEM, Gibco, USA) with 10% FBS. Cell culture was maintained at 37 °C in 5% CO2 in 100% humidity.

### Dicer-substrate mediated transfection

To knockdown RAMS11, Dicer-substrate mediated silencing was performed. HCT116 and SW480 cells were seeded and cultured in 6-well plate. Transfection experiment was performed when cell density reached 60–70% confluence. A lipid-based in vitro transfection was carried out by Lipofectamine 2000 (Invitrogen, USA), according to the manufacturer’s protocol. TriFECTa Kits were purchased from Integrated DNA Technologies (IDT, USA) which contained a Dicer-substrate negative control (DSi-NC), positive control (Dsi-HPRT-S1), transfection control (Dsi-TYE 563) and predesigned Dsi-RAMS11 (target genes) duplex. The duplex sequences for Dsi-RAMS11 were: 5’–GAAUAAACAGGAUGUCUCUCACUTT-3’ and 3’–GACUUAUUUGUCCUACAGAGAGUGAAA-5’. The Dsi-NC and Dsi-HPRT-S1 sequence were not provided by the manufacturer. The Dsi-NC and Dsi-HPRT-S1 sequence were not provided by the manufacturer. The transfection conditions were optimized in preliminary experiments.

### RNA isolation and qRT-PCR

The total RNA from the colon cells were extracted using RNeasy mini kit (Qiagen, Germany) according to their guidelines. The RNA concentration was measured by NanoDrop200 (Thermo Scientific, USA). Following the standard protocol, first-strand cDNA was synthesized using Superscript II and Random Hexamer (Invitrogen, USA). Master Mix LightCycler 480 SYBR Green I (Roche, Switzerland) was used to complete the quantitative reaction using LightCycler 480 Instrument II (Roche, Switzerland). In order to get consistent results, melting temperature (Tm) 60 ± 2 °C and 45 cycles of amplification were followed. Detection of PCR product was based on SYBR green fluorescence signals. The melting curve analysis was performed to ensure specific target detection. Here, GAPDH was considered as the housekeeping gene and relative expression was calculated by 2^−△△Ct^ method.

### Cell viability assay

After 24 h of transfection, cells were trypsinized and counted by haemocytometer for seeding and performing cell proliferation assay using Cell Counting Kit-8 (CCK-8, Dojindo). 5 × 10^3^ cells in 100 µl of complete medium was seeded and cultured in a 96-well plate. According to CCK-8 cell proliferation assay protocol, 10 µL of CCK-8 solution was added to the well. After 3 h incubation at 37 °C + 5% CO_2_, the amount of formazan which represents the number of live cells were measured at absorbance 450 nM using SPECTROstar Nano Microplate Reader (BMG Labtech, Germany).

### Colony formation assay

Colony formation assay was performed to measure the cell proliferation in vitro. After being transfected for 24 h, 1 × 10^3^ cells were seeded and cultured for around two weeks in 6-well plate in triplicates. After colony formation, the colonies were fixed with a mixture of methanol and acetic acid at a ratio of 3:1. A solution of 0.5% crystal violet in methanol was used to stain and visualize the colonies. The images were photographed and the number of colonies were counted by ImageJ software, National Institutes of Health (NIH).

### Migration assay

In migration assay, 5 × 10^4^ cells in 70 µl DMEM with 10% FBS were carefully placed in both compartments of the Culture-Insert 2 Well (Ibidi LLC, Germany). After 24 h of cells settling, the culture inserts were gently removed by using tweezers to create a space of ~ 500 µm for measuring the cell migration ability. Then, each well was filled with 1.5 ml of complete medium. The photographs of the wound areas were taken using an inverted microscope (Nikon, Japan) at various time point of 0, 24 and 48 h respectively. The migration index indicating the size of the gap was measured using the MRI Wound Healing Tool in ImageJ (NIH).

### Western blotting

Western blotting was performed using standard, established protocol as previously published [19]. Briefly, protein isolation was performed using RIPA lysis and extraction buffer (Thermo Scientific, USA) with a supplement of cOmplete ULTRA Tablets, Mini EDTA-free, Easy pack Protease Inhibitor Cocktail (Roche, Switzerland). Protein concentration was quantified using BCA Protein Assay Kit (Thermo Fisher Scientific, USA), and similar amount of proteins were loaded and run on 8–12% SDS-PAGE at ambient temperature. Proteins were then transferred onto Immun-Blot PVDF Membrane (Bio-Rad Laboratories, Inc, USA), and followed by two hours blocking in 5% bovine serum albumin (BSA) (Hyclone BSA, GE Healthcare Life Science, USA) in Tris-buffer saline with a supplement of 0.1% tween 20 (TBST). Then the blocked-membrane were incubated overnight with primary antibodies: β-actin (#8457, Cell signalling technology, Inc., (CST, USA)), GAPDH (#2118, CST), AKT (#9272), Phophor-AKT (#9271, CST), AMPKα (#5832, CST), phosphor-AMPKα (#2535, CST), Bcl-2 (#2872, CST), Bcl-xL (#2764, CST), Beclin-1 (#3738, CST), Caspase-9 (#9502, CST), E-cadherin (#3195, CST), N-cadherin (#13116S, CST), LC3B (#2775, CST), p62 (#5114, CST), mTOR (#2972, CST), Phosphor-mTOR (#2535, CST), Snail (#3879, CST), Sox2 (#3579, CST), and Vimentin (#5741S, CST) at 4 °C. The secondary anti-rabit IgG, Horseradish peroxide (HRP)-linked or anti-mouse IgG-HRP-linked (#7076, CST) antibody were added and incubated with the membrane for two hours. Afterwards, Western Lightning Plus-Electrochemiluminescence (PerkinElmer, Inc., USA) was added to the membrane to visualize protein bands in a ChemiDoc MP Imaging System (Bio-Rad Laboratories, Inc, USA). The relative protein expressions were quantified using ImageJ software (NIH) with β-actin or GAPDH as internal control.

### Statistical analysis

All data are presented as mean ± standard error of mean (SEM) of at least three or more independent experiments. The statistical differences of the experimental data were calculated by student’s t test or one way ANOVA using GraphPad Prism version 8.0 (GraphPad Software, Inc., San Diego, CA, USA). The value of *P* < 0.05 is considered statistically significant.

## Results

### RAMS11 is highly overexpressed in CRC cell lines and can be downregulated by Dicer-substrate siRNA techniques

**Fig. 1:**
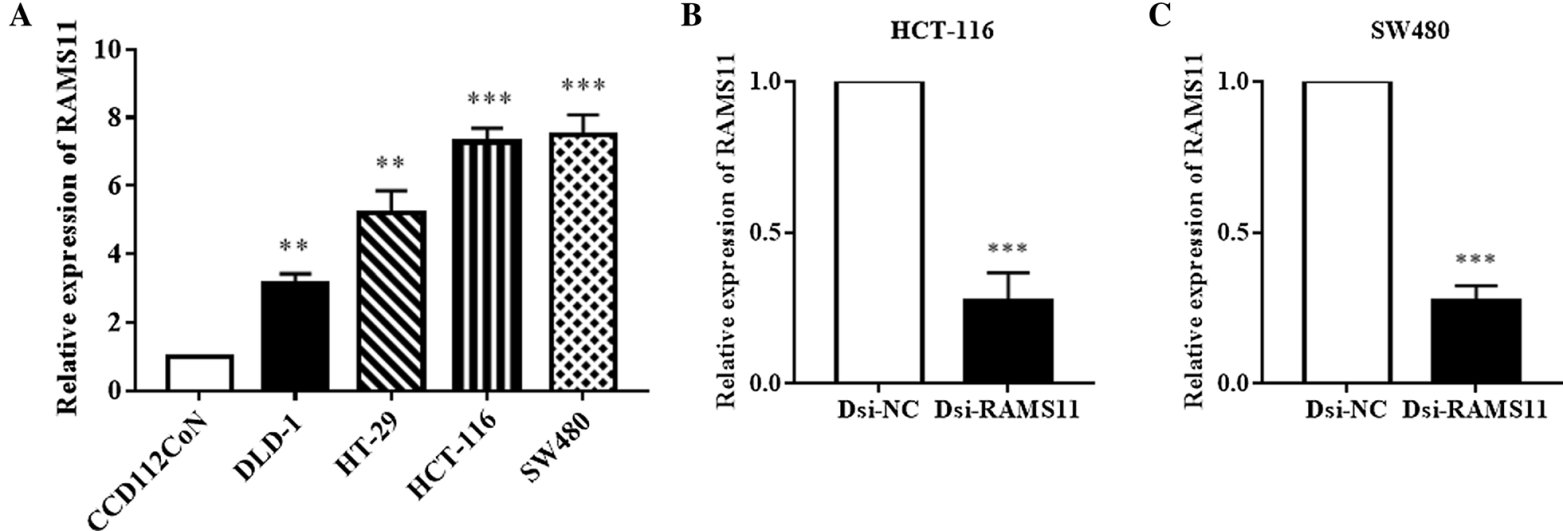
RAMS11 expression in colon cells and effective knockdown by Dicer-substrate siRNA techniques. **A** RAMS11 expression was measured in CRC cell lines (DLD-1, HT-29, HCT-16, and SW480) and colon normal cells (CCD-112-CoN) by using RT-qPCR. **B**, **C** The Dicer-substrate mediated RAMS11 knockdown was performed to downregulate the RAMS11 expression in HCT-116 and SW480 cells. The data was shown as mean ± SEM compared to normal cells, and negative control (Dsi-NC) group. (*P < 0.05, **P < 0.01, ***P < *0.001,* and n = 4)

The expression of RAMS11 was confirmed in CRC cell lines (DLD-1, HT-29, HCT-116, and SW480) and normal colon cells CCD-112CoN by RT-qPCR as shown in Fig. 1. Our results indicated that RAMS11 was significantly overexpressed in CRC cell lines compared to the normal cell line CCD-112CoN (Fig. 1A) suggesting that the RAMS11 expression may be associated with the carcinogenesis of CRC. RAMS11 was most abundantly expressed in SW480 > HCT-116 > HT-29 > DLD-1 respectively. Therefore, SW480 and HCT-116 were selected for Dicer-substrate mediated gene knockdown assay (Fig. 1B, C). The knockdown efficacy and efficiency of Dsi-RAMS11 compared to Dicer-mediated negative control (Dsi-NC) was evaluated using RT-qPCR and we confirmed more than 70% silencing in both HCT-116 (Fig. 1B) and SW480 (Fig. 1C) cells.

### Downregulation of RAMS11 inhibited cell proliferation, colony formation and migration of CRC cells

**Fig. 2:**
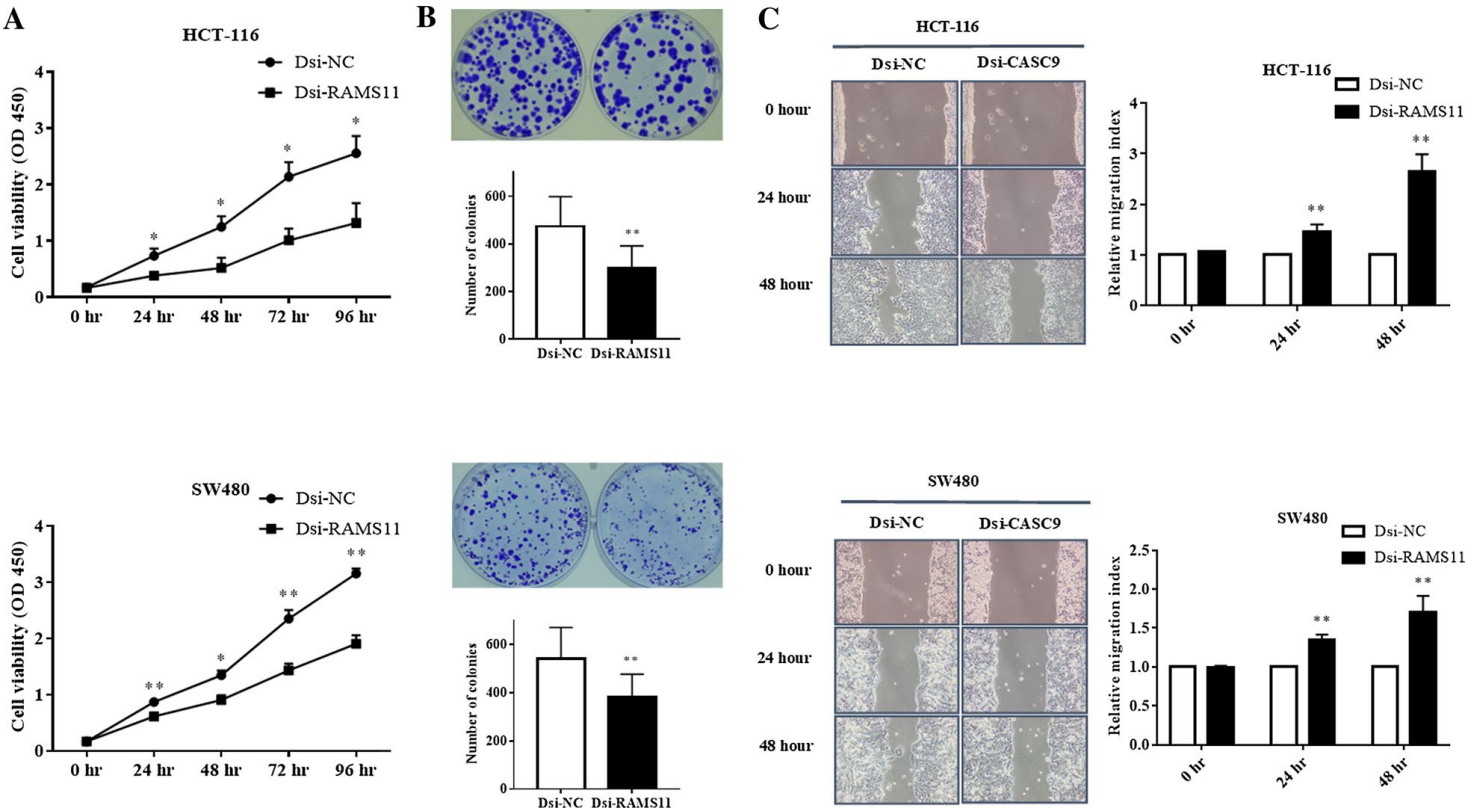
Downregulation of RAMS11 inhibited CRC cells proliferation, growth, and migration. **A** CCK-8 assay was performed to evaluate the proliferation of HCT-116 and SW480 cells after Dsi-RAMS11 transfection. Downregulation of RAMS11 significantly reduced both cells proliferation at 24, 48, 72, and 96 h of the experiments. **B** The number of colonies were also significantly decreased in both cells after Dsi-RAMS11. **C** The wound healing migration assay was performed to measure the migration index of HCT-116 and SW480 cells after Dsi-RAMS11. It shown that after RAMS11 knockdown, the migration index of HCT-116 and SW480 cells were significantly increased at 24 and 48 h of experiments compared to Dsi-NC. The data was shown as mean ± SEM compared to Dsi-NC group. (*P < 0.05, **P < *0.01,* and n = 4)

To understand the functional roles of RAMS11 in CRC proliferation, growth, and migration, we performed CCK-8 cell proliferation assay, colony formation assay, and migration assay. Our CCK-8 results showed that downregulation of RAMS11 significantly reduced cell proliferation of HCT-116 and SW480 cells compared to negative control Dsi-NC at 24, 48, 72, and 96 h after Dsi-RAMS11 transfection (Fig. 2A). In concordance with the cell proliferation results, the colony formation assay showed that downregulation of RAMS11 significantly decreased the number of colonies in both HCT-116 and SW480 cells (Fig. 2B) compared to the Dsi-NC group. Next, the wound healing migration assay was performed to demonstrate the migration ability of HCT-116 and SW480 cells. Our results confirmed a significant higher migration index of HCT-116 and SW480 cells after Dsi-RAMS11 treatment at 24 and 48 h post-transfection compared to negative control Dsi-NC (Fig. 2C).

### Downregulation of RAMS11 promoted autophagy in CRC cells

**Fig. 3:**
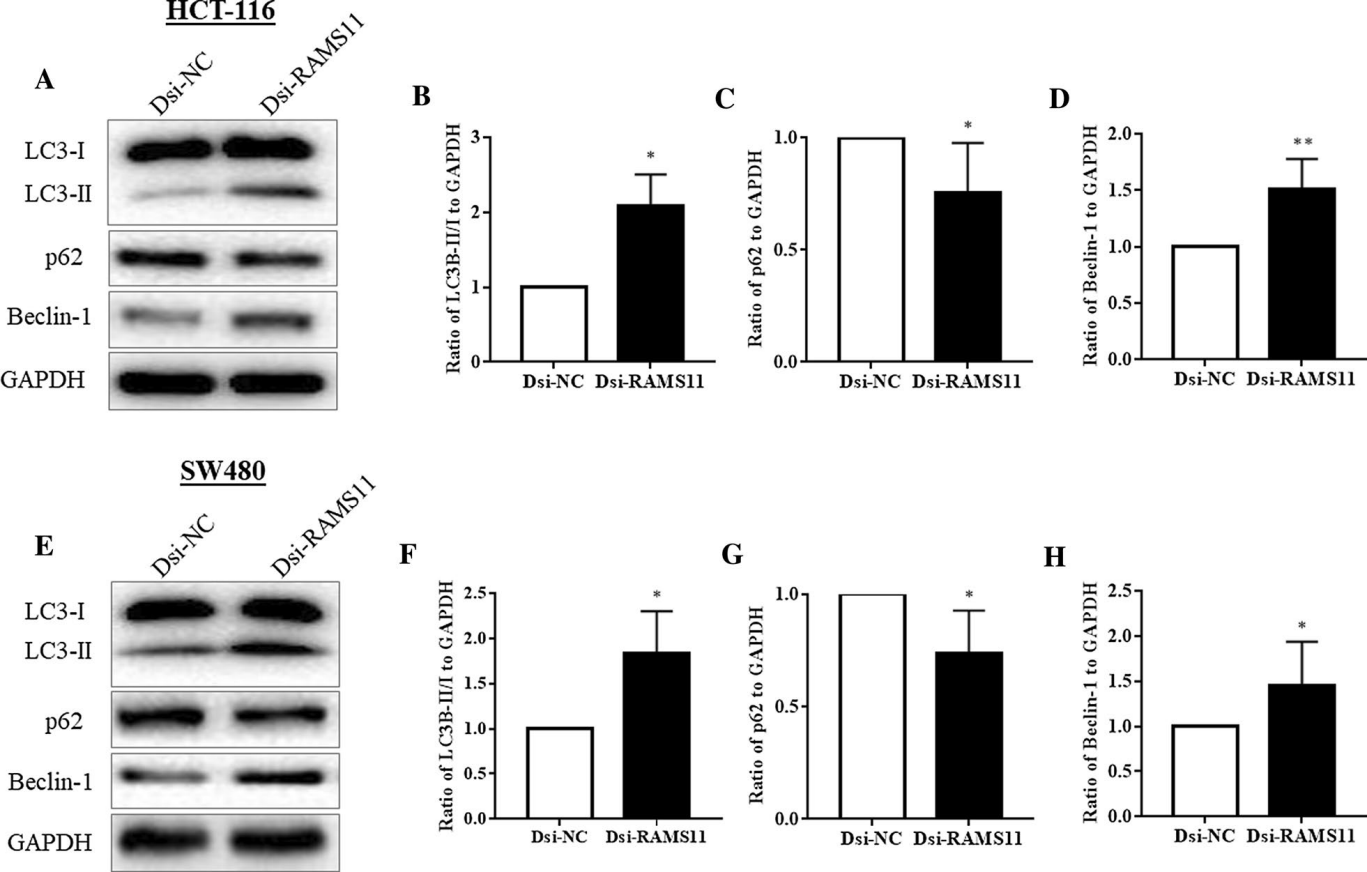
Downregulation of RAMS11 promoted autophagy in CRC cells. The autophagy marker proteins LC3B, p62, and Beclin-1 were measured using western blot in both HCT-116 **A**–**D**, and SW480 **E–H** cells. The Dsi-RAMS11 treated cells increased ratio of LC3-II/LC3-I, and Beclin-1 in both HCT-116 and SW480 cells, whereas reduced p62 expressions. The data was shown as relative expression mean ± SEM compared to Dsi-NC group using GAPDH as housekeeping gene. (*P < 0.05, **P < *0.01,* and n = 4)

Autophagy is one of key regulatory self-degradative process of cells to maintain homeostasis, and survival during stress and hypoxic conditions. The autophagy levels in CRC cells HCT-116 and SW480 were evaluated by analysing autophagy marker proteins LC3B, p62, and Beclin-1 using western blot after Dsi-RAMS11 transfection (Fig. 3). Our results demonstrated that downregulation of RAMS11 significantly increased LC3B expression in HCT-116 (Fig. 3A, B) and SW480 (Fig. 3E, F) cells. Silencing of RAMS11 significantly suppressed the expression of p62 in both HCT-116 (Fig. 3C) and SW480 (Fig. 3G) cells. Furthermore, we evaluated Beclin-1 expression which is associated with cellular key regulatory process autophagy and cell death. The western blot result showed that Dsi-RAMS11 significantly promoted Beclin-1 expression compared to Dsi-NC in HCT-116 (Fig. 3D) and SW480 (Fig. 3H) cells. Overall, silencing of RAMS11 increased LC3B and Beclin-1 expression to induced autophagy and reduced p62 expression to activate autophagic flux.

### Downregulation of RAMS11 increased apoptosis of CRC cells

**Fig. 4:**
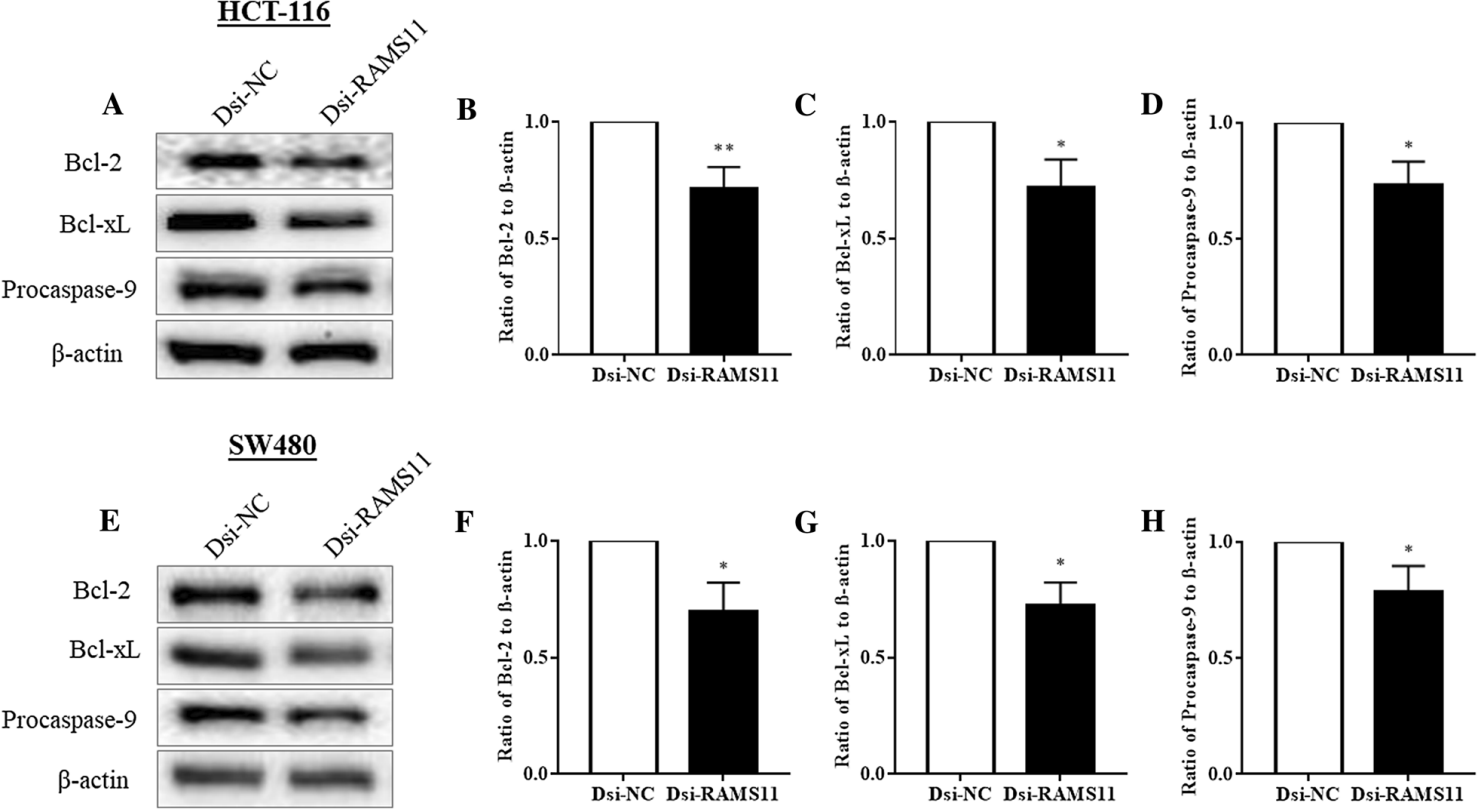
Downregulation of RAMS11 reduced the expression of apoptosis suppressors**.** The key apoptosis markers Bcl-2, Bcl-xL, and procaspase-9 were evaluated by western blotting in **A**–**D** HCT-116 and **E–H** SW480 cells. Downregulation of RAMS11 significantly reduced the expressions of Bcl-2, Bcl-xL, and procaspase-9 in both HCT-116 and SW480 cells compared to negative control Dsi-NC. The data was shown as relative expression mean ± SEM compared to Dsi-NC group using β-actin as housekeeping gene. (*P < 0.05, **P < *0.01*, and n = 4)

After confirmation of autophagy, we further explored the roles of RAMS11 in apoptosis (Fig. 4). Bcl-2 regulates apoptosis by inhibiting apoptosis. Overexpression of Bcl-2 in cancer cells may block apoptosis and enhance cell survival. In our experiment the silencing of RAMS11 led to significant reduction of Bcl-2 in HCT-116 (Fig. 4A, B) and SW480 (Fig. 4E, F) cells. Similarly, another Bcl-2 family protein Bcl-xL was downregulated in HCT-116 (Fig. 4C) and SW480 (Fig. 4G) cells. Similarly, procaspase-9 expression was also reduced in both cells after Dsi-RAMS11 (Fig. 4D, H). These results suggested that knockdown of RAMS11 promotes apoptotic cell death.

### Downregulation of RAMS11 inhibited AKT/mTOR signalling via promoting AMPK pathway

**Fig. 5:**
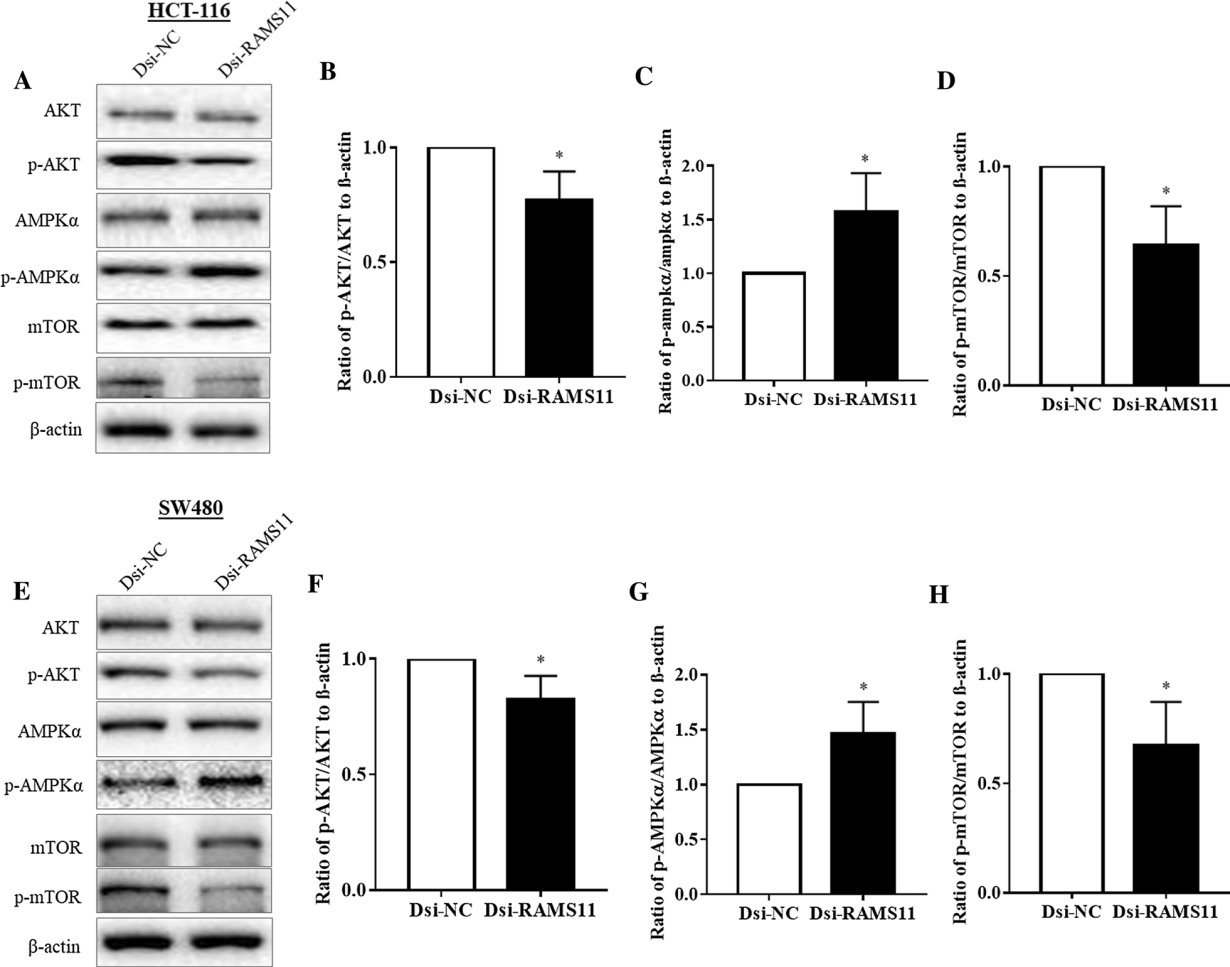
Knockdown of RAMS11 downregulated AKT/mTOR signalling by promoting AMPK pathway. Downregulation of RAMS11 significantly reduced the phosphorylation of AKT and mTOR and induced phosphorylation of AMPK in **B**–**D** HCT-116 and **F–H** SW480 cell. The data was shown as relative expression mean ± SEM compared to Dsi-NC group using β-actin as housekeeping gene. (*P < *0.05,* and n = 4)

Further investigation of the signalling pathways involving RAMS11 was performed by investigating the most frequently altered mTOR pathways with its upstream and downstream targets in CRC. To determine the AKT/AMPKα/mTOR signalling pathway, the phosphorylation of these proteins were analysed. As shown in Fig. 5A and E, the expressions of AKT, AMPKα, and mTOR in both Dsi-NC and Dsi-RAMS11 samples remained unchanged in both HCT-116 and SW480 cells. However, the expression of phosphorylated proteins p-AKT and p-mTOR expression in HCT-116 (Fig. 5B, D) and SW480 (Fig. 5F, H) cells were downregulated after Dsi-RAMS11 transfection. We also confirmed that Dsi-RAMS11 significantly increased the p-AMPK expression in both cell lines (Fig. 5C, G) compared to the Dsi-NC group. This results indicated that Dsi-RAMS11 may promote the activation of AMPK by reducing phosphorylation of p-AKT and p-mTOR.

### Downregulation of RAMS11 negatively altered EMT marker proteins and transcription factors

**Fig. 6:**
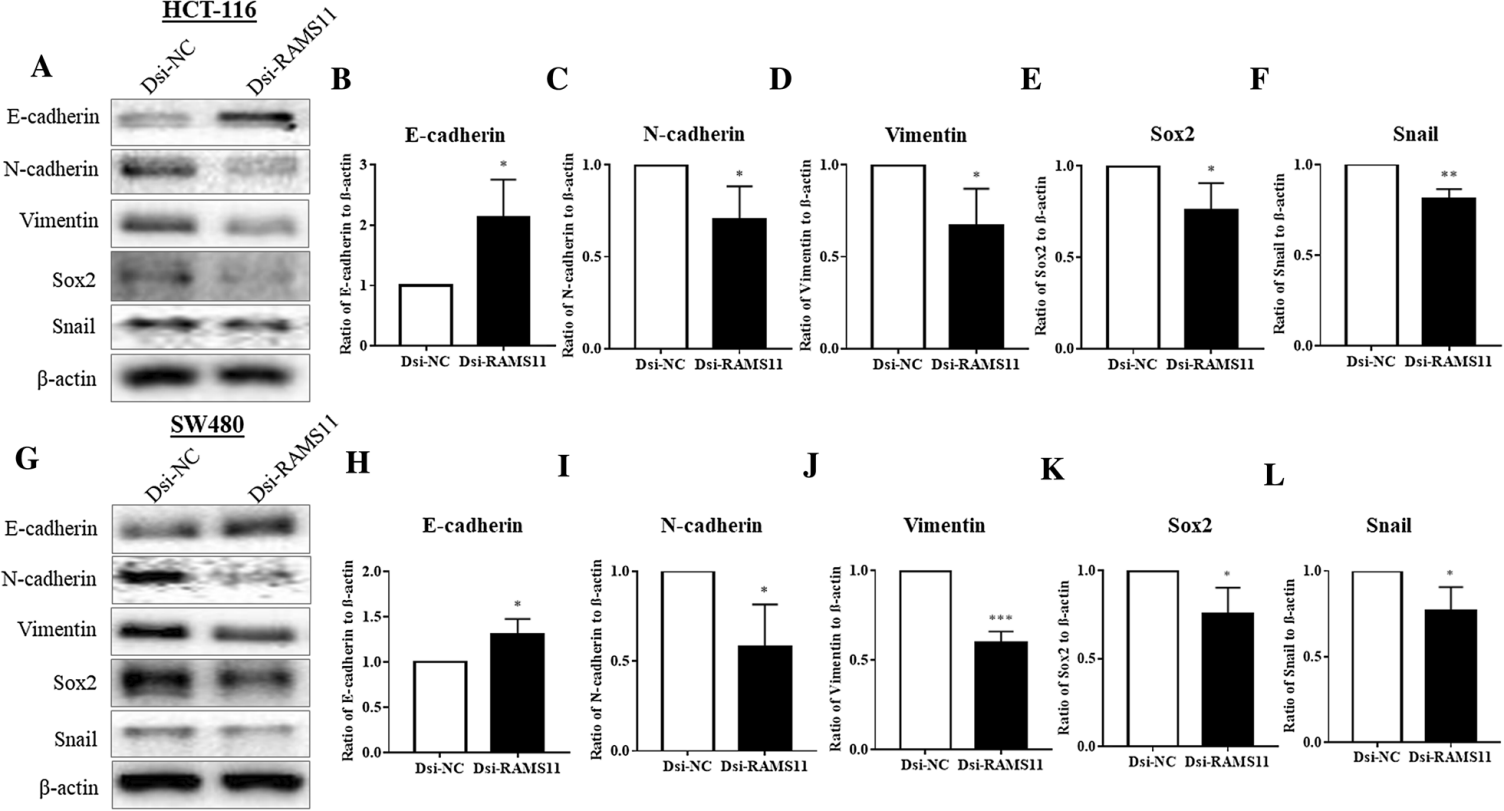
The knockdown of RAMS11 inhibited EMT markers and transcription factors in CRC cells. The EMT markers and transcription factors expressions were evaluated by western blotting in **A**–**F** HCT-116 and **G**–**L** SW480 cells. The Dsi-RAMS11 mediated knockdown significantly enhanced the expression of E-cadherin in both **B** HCT-116 and **H** SW480 cells compared to Dsi-NC. On the other hand, Dsi-RAMS11 significantly decreased mesenchymal markers N-cadherin and vimentin expressions in **C**, **D** HCT-116 and **I**, **J** SW480 cells. In addition, EMT regulated transcription factors Sox2 and Snail expressions were significantly downregulated in **E**, **F** HCT-116 and **K**–**L** SW480 cells after Dsi-RAMS11. The data was shown as relative expression mean ± SEM compared to Dsi-NC group using β-actin as internal control. (*P < 0.05, **P < *0.01*, ***P < *0.001* and n = 4)

We subsequently evaluated the expression of EMT marker proteins in CRC cell lines and evaluated the effect of RAMS11 gene silencing. Cancer cells gain migratory characteristics resulting in development of metastasis, chemo-resistance, and immune-suppression via EMT pathways. In EMT, the epithelial marker E-cadherin level is decreased, and mesenchymal proteins and transcription factors N-cadherin, vimentin, Sox2, and Snail levels are increased. In our study, we found that Dsi-RAMS11 significantly increased the epithelial marker E-cadherin in HCT-116 (Fig. 6B) and SW480 (Fig. 6H) cells compared to Dsi-NC. On the other hand, Dsi-RAMS11 significantly decreased the mesenchymal marker proteins N-cadherin and vimentin in both cell lines HCT-116 (Fig. 6C, D) and SW480 (Fig. 6I, J). Apart from that, we also evaluated the expressions of EMT regulated transcription factors Sox2 and Snail. Our results showed significant reductions of Snail and Sox2 expressions after Dsi-RAMS11 treatment in HCT-116 (Fig. 6E, F) and SW480 (Fig. 6K, L) cells.

## Discussion

Human genomic research using high-throughput next-generation sequencing (NGS) technologies revealed very few and inconsiderable amounts of protein-coding regions in the humans genome. Although predominant parts of RNA are transcribed, very small amount (≤ 2%) are capable of producing proteins [20]. The remaining major part (> 90%) of the human genome is therefore considered as non-coding RNAs (ncRNAs). Initially ncRNAs were thought to be non-functional junk molecules. The advancement of sequencing and bioinformatics analysis have now identified more and more ncRNAs with potential biological functions [21]. Among various kind of ncRNAs, the novel and rapidly emerging lncRNAs are ranked at high priority by researchers because of their involvement in diverse molecular events such as, transcriptional regulator, nuclear regions organization, and control of proteins molecules [22]. Modern research revealed that lncRNAs work as promoters of other RNAs from both sense and antisense strands to overlap genes, encode small proteins, and sometimes even act as small genes [23, 24]. LncRNAs are abundantly expressed in most cancers to alter normal biological processes. Their diverse expressions and mutations are inheritably connected with tumorigenesis, metastasis, and different tumor stages [21, 25, 26]. Importantly, the abundant expressions of lncRNAs from malignant tumors can be detected from circulatory blood or urine samples [27, 28]. Thus, lncRNAs extended its application as discovery of potential biomarkers and therapeutic indicators of cancer to improve treatment outcomes.

In this study, we are the first to demonstrate that lncRNA RAMS11 is associated with CRC progression and metastasis in vitro. Our study demonstrated that downregulation of RAMS11 is negatively associated with CRC cell proliferation, growth, and metastasis via autophagy, apoptosis, and AKT/AMPKα/mTOR signalling pathways.

Previous studies have reported that knockdown or downregulation of many carcinogenic genes or lncRNAs could potentially inhibit the tumour progressions [29, 30]. Hence, we knockdown RAMS11 and perform in vitro assays including cell proliferation, colony formation and migration assay and it was revealed that RAMS11 downregulation significantly reduced CRC cell survival, proliferation, and migration. Our results are in line with previous studies that demonstrated knockdown or knockout of lncRNAs, such as H19, SNHG14, CCAL, and MIR17HG [31–35], potentially reduced CRC cell proliferation, migration, metastasis, and increased chemo-sensitivity.

Several previous studies have shown that lncRNAs exert its oncogenic roles by regulating most commonly mutated mTOR with its upstream and downstream signalling pathways [36, 37]. Moreover, lncRNAs mediated inhibition or progression of cancer is regulated by mTOR-dependent or independent autophagy pathway. Autophagy is the cellular lysosomal degradative process of removing unnecessary or folded materials to maintain homeostasis and restore energy during nutrient stress and hypoxic conditions [38]. It is well established that inhibition of autophagy reduced elimination of damaged particles, accumulate folded materials from cells and results in cancer development [39]. In our exploration, it is suggested that RAMS11 silencing mediated autophagy induction reduced CRC cell growth and proliferation and our findings also implied that RAMS11 suppressed autophagy in CRC cell lines.

Most chemotherapy drugs promote programmed cell death process called apoptosis, however chemo-resistant cells does not respond to the therapy [40]. Apoptosis pathway is maintained by pro-apoptotic and pro-survival proteins that establish balance between cell survival and death by regulating Bcl-2 family proteins [41]. The mitochondrial containment of Bcl-2 participate in intrinsic apoptosis by restricting oligomerization of BAX or BAK responsible for extended cell cycle [41]. Bcl-2, Bcl-xL overexpression reduce apoptosis and facilitate immortalization of damaged cells, resulting in excessive proliferation and tumour development [41, 42]. In addition, a protein complex of cytochrome C, APAF1, and dATP form apoptosome in cytosol, which initially activate caspase 9 and followed by activation of caspase 3, 6, and 7 to stimulate cellular phagocytosis process [41, 43, 44]. Connecting our findings on cell proliferation, autophagy, and apoptosis, we suggest that RAMS11 support cell proliferation in CRC by downregulating autophagy and apoptosis process.

The AKT/AMPK/mTOR signalling is the major regulatory pathways associated with cellular autophagy, apoptosis, cell proliferation, migration, and angiogenesis in cancer [45, 46]. The serine-threonine protein kinase mTOR consist of two functionally distinct complexes called mTORC1 and mTORC2. In order to maintain cellular growth, proliferation, migration, apoptosis, and autophagy, the protein complexes mTORC1 and mTORC2 are activated by various stimulus, such as nutrient deprivation, stress, growth factors, and potential regulatory signallings (e.g. PI3K, AKT, MAPK, and AMPK) [47]. Studies have shown that mTORC1 activation inhibits autophagy induction whereas mTORC2 indirectly activates mTORC1 to suppress autophagy [46, 48]. The PI3K pathway activates mTORC2 by phosphorylatings AKT resulting activation of AKT and mTORC1 [46]. Another key regulatory signalling in mTORC1 dependent autophagy is AMPK, which also is activated in nutrient deprivation and stress condition [49]. Therefore, AMPK is considered to be an “energy controller” of eukaryotic cells. The phosphorylation of AMPK induce autophagy by restricting mTORC1 and activating several murine proteins Ser317, Ser777, and Ser555 in stress and energy starvation conditions [46, 48–50]. In this study, our key pathway investigation revealed that Dsi-RAMS11 potentially induced autophagy and apoptosis by phosphorylation of AMPK and inhibition of AKT and mTOR signalling.

The EMT process activation comprises of losing intracellular adhesion and polarity to increase migratory and invasive properties of cells [51]. The EMT induction promoted epithelial marker E-cadherin whereas reduced mesenchymal maker proteins N-cadherin, vimentin, and fibronectin [52]. In addition, the EMT process is regulated by a number of transcription factors such as Snail, Sox2, ZEB1, and TWIST [52, 53]. These transcription factors regulate EMT by direct or indirect regulation of EMT marker proteins [52, 53]. Snail activates EMT by reducing E-cadherin and claudins, and increasing vimentin and fibronectin in cancer [54]. Another well-established stem cell marker Sox2 play crucial roles in initiation and progression of carcinogenesis [55]. The previous research demonstration revealed that Sox2 knockdown potentially induces mesenchymal to epithelial transition (MET) process in CRC cells along with E-cadherin and vimentin via regulating Wnt pathway [55]. In the current study, we demonstrated that RAMS11 may promote CRC progression and development of metastasis by achieving EMT regulated invasive and migratory characteristics of CRC cells. Whereas, silencing of RAMS11 reverse the process by reducing EMT. Moreover, this findings explain the reduced growth and metastasis observed in the RAMS11 knockout mice previously [18].

## Conclusions

In summary, our study described the oncogenic roles of RAMS11 in CRC. We also demonstrated that downregulation of RAMS11 may provide a new branch of targeted therapy and better understanding of carcinogenesis via mTOR dependent activation of autophagy, promotion of apoptosis, and inhibition of EMT process. However, one important limitation of this study is the absence of in vivo demonstrations which might allow us to make stronger conclusions of our findings and to support RAMS11 as a useful cancer biomarker for CRC.

## Supplementary Information

**Additional file 1.** FASTA sequence of RAMS11

**Additional file 2.** Original data of the results

## Data Availability

All data generated and analysed during conducting this study are included in this article and its Additional file 1 and Additional file 2. The raw data associated with RAMS11 expression can be made available upon request.

## Abbreviations

ATCC: American Type Culture Collection
AMPK: AMP-activated protein kinase
CCK-8: Cell counting kit-8
CRC: Colorectal cancer
DMEM: Dulbecco’s modified eagle medium
EMEM: Eagle’s minimum essential medium
EMT: Epithelial-mesenchymal transition
FBS: Fetal bovine serum
GLOBOCAN: Global Cancer Incidence, Mortality and Prevalence
LncRNAs: Long-non coding RNAs
MET: Mesenchymal to epithelial transition
NIH: National Institutes of Health
NGS: Next-generation sequencing
ncRNAs: Non-coding RNAs
RAMS11: RNA associated with metastasis-11
