# Supplementary material for: RAMS11 promotes CRC through mTOR-dependent inhibition of autophagy, suppression of apoptosis, and promotion of epithelial-mesenchymal transition
Source: Cancer Cell Int. 2021 Jun 26;21:321. doi: 10.1186/s12935-021-02023-6 (PMC8236194; doi:10.1186/s12935-021-02023-6)

Original data for Figure 2

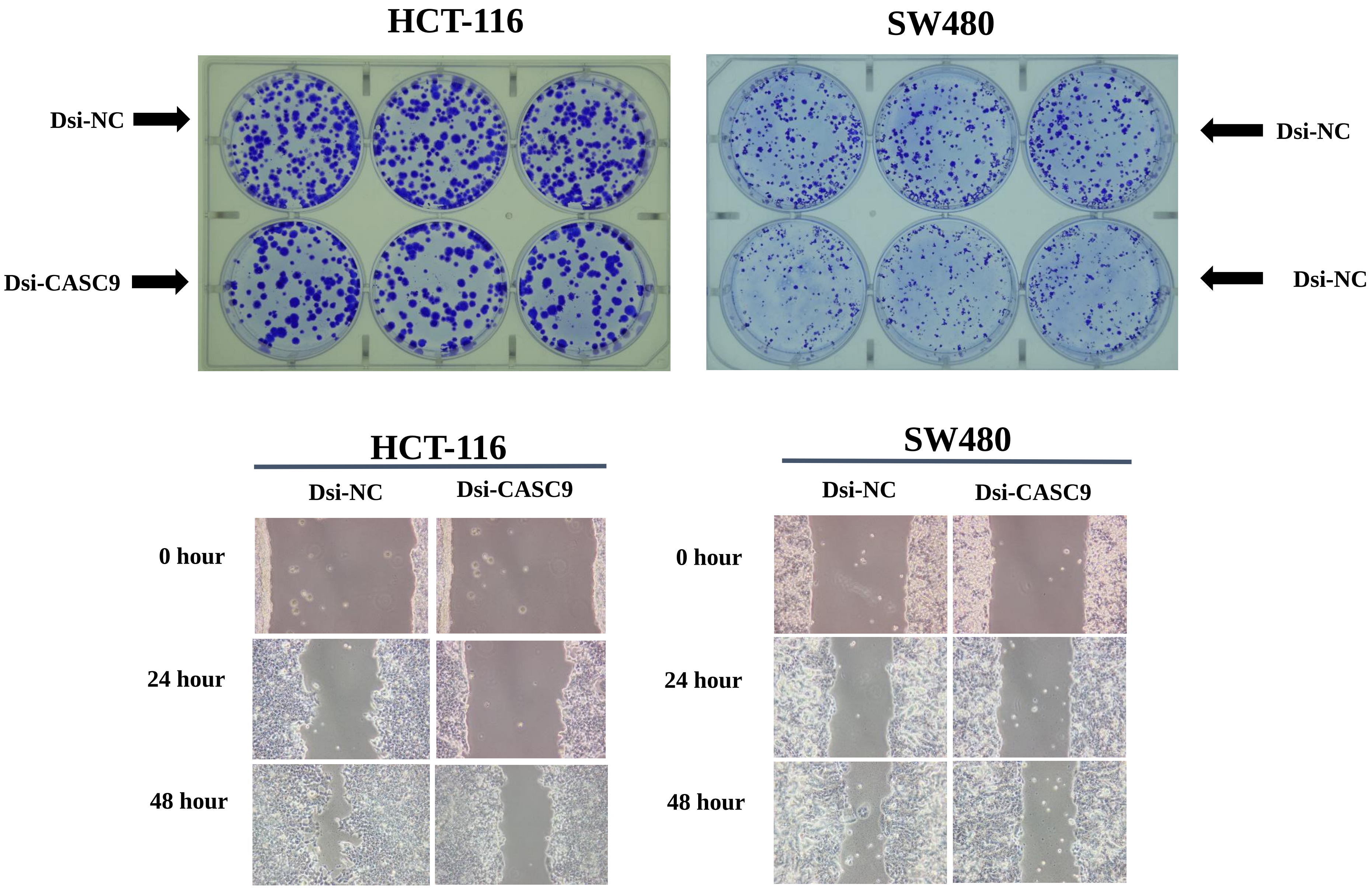

Original data for Figure 3

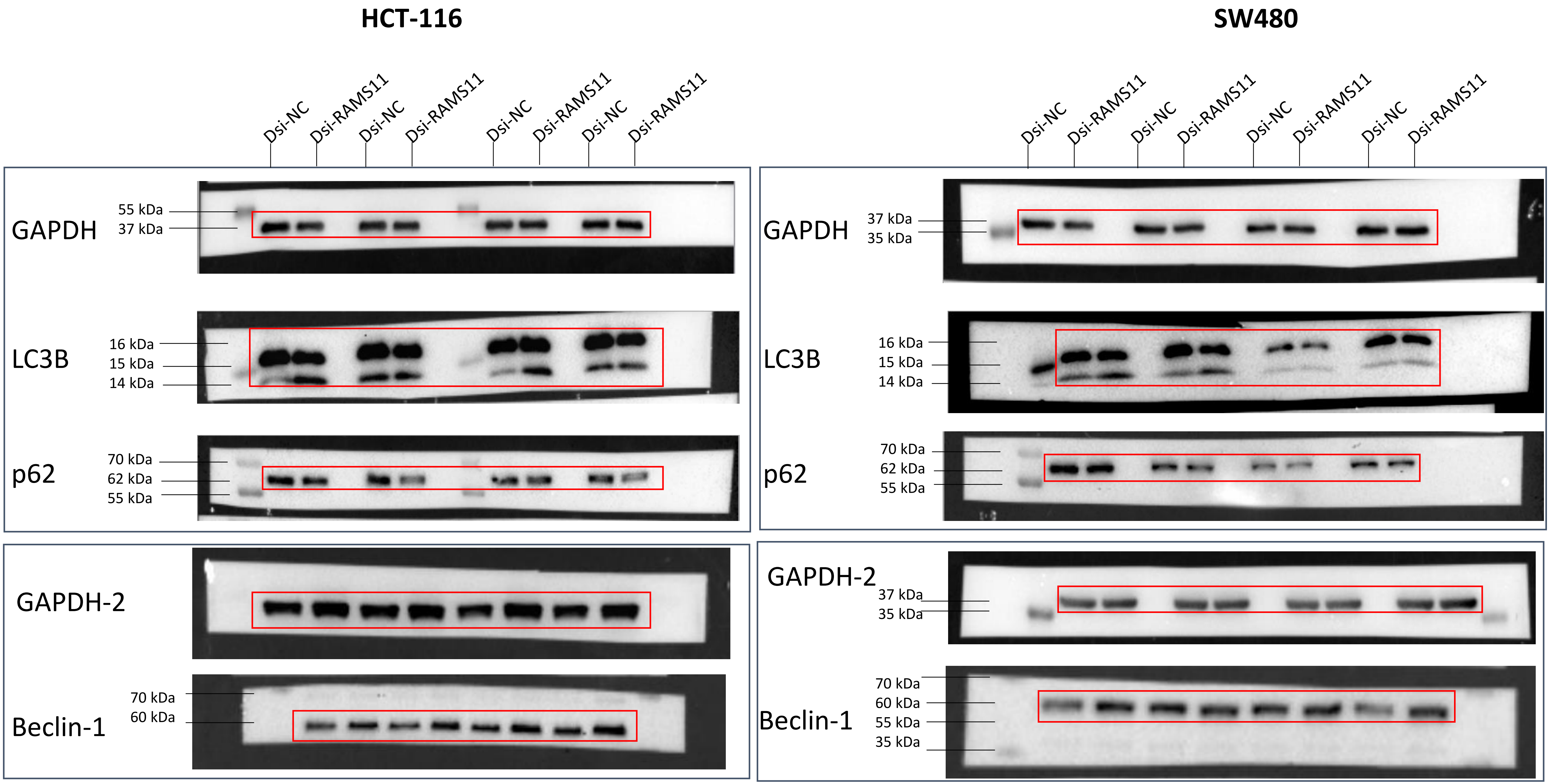

Original data for Figure 4

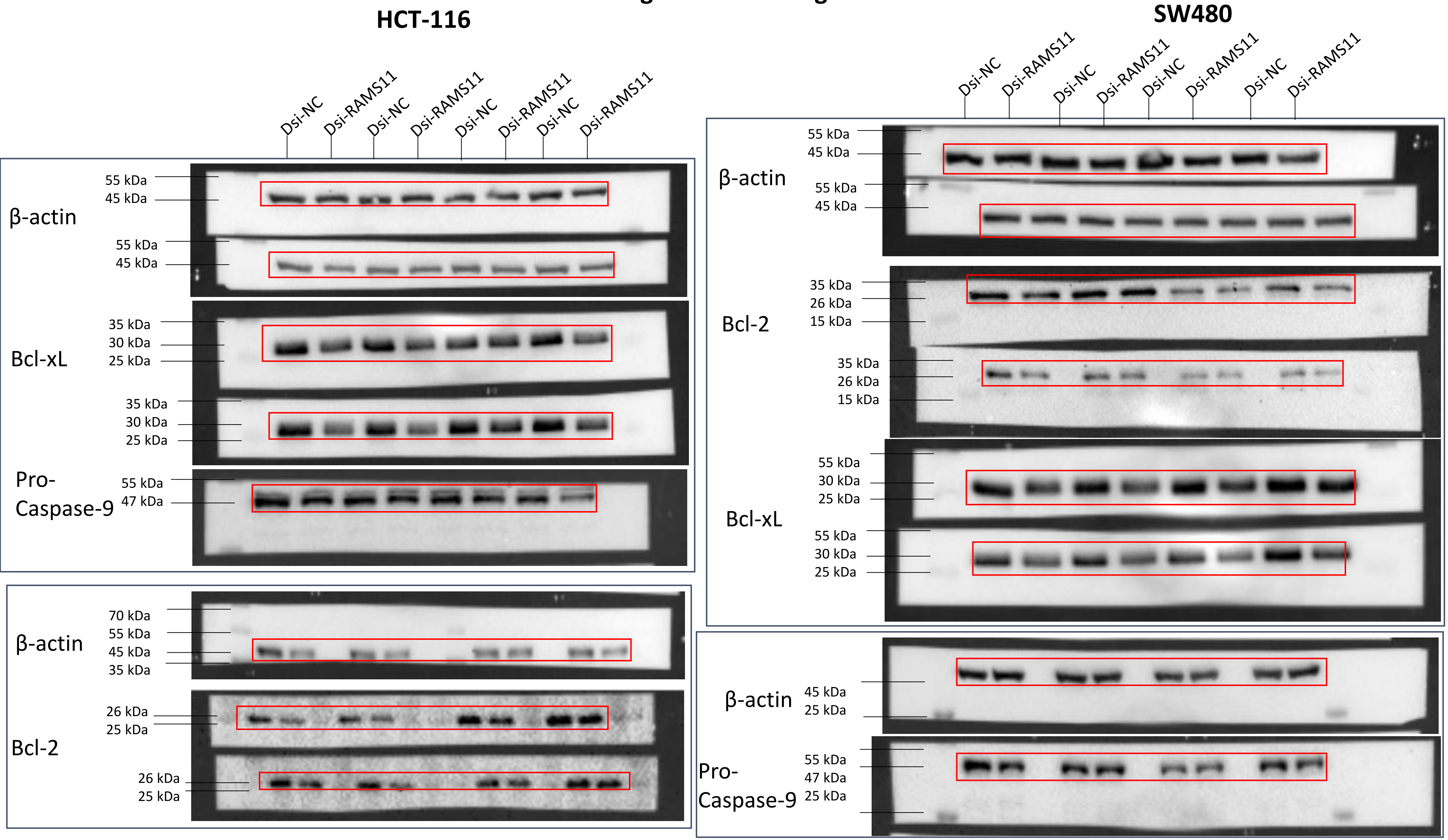

Original data for Figure 5

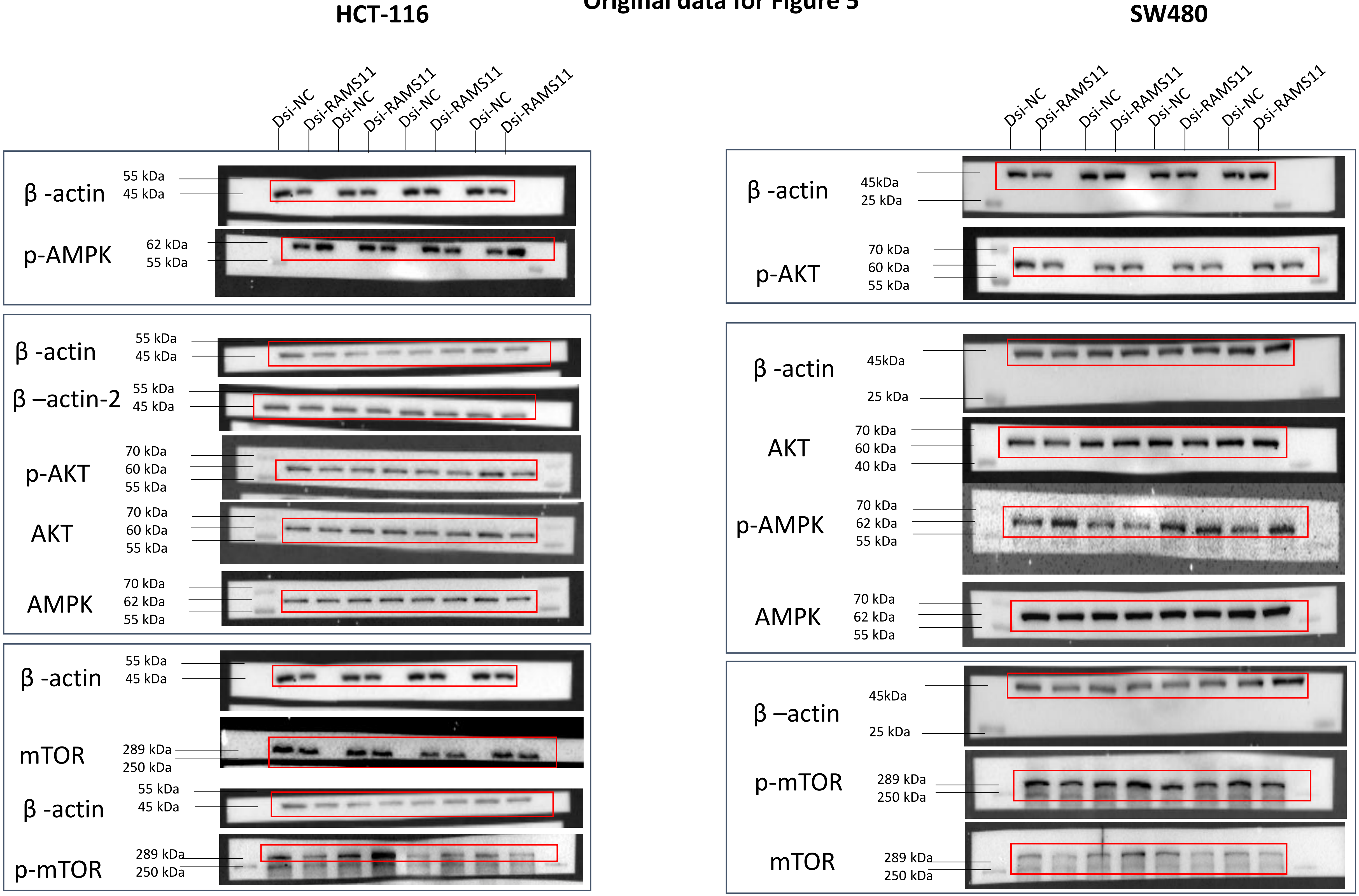

Original data for Figure 6

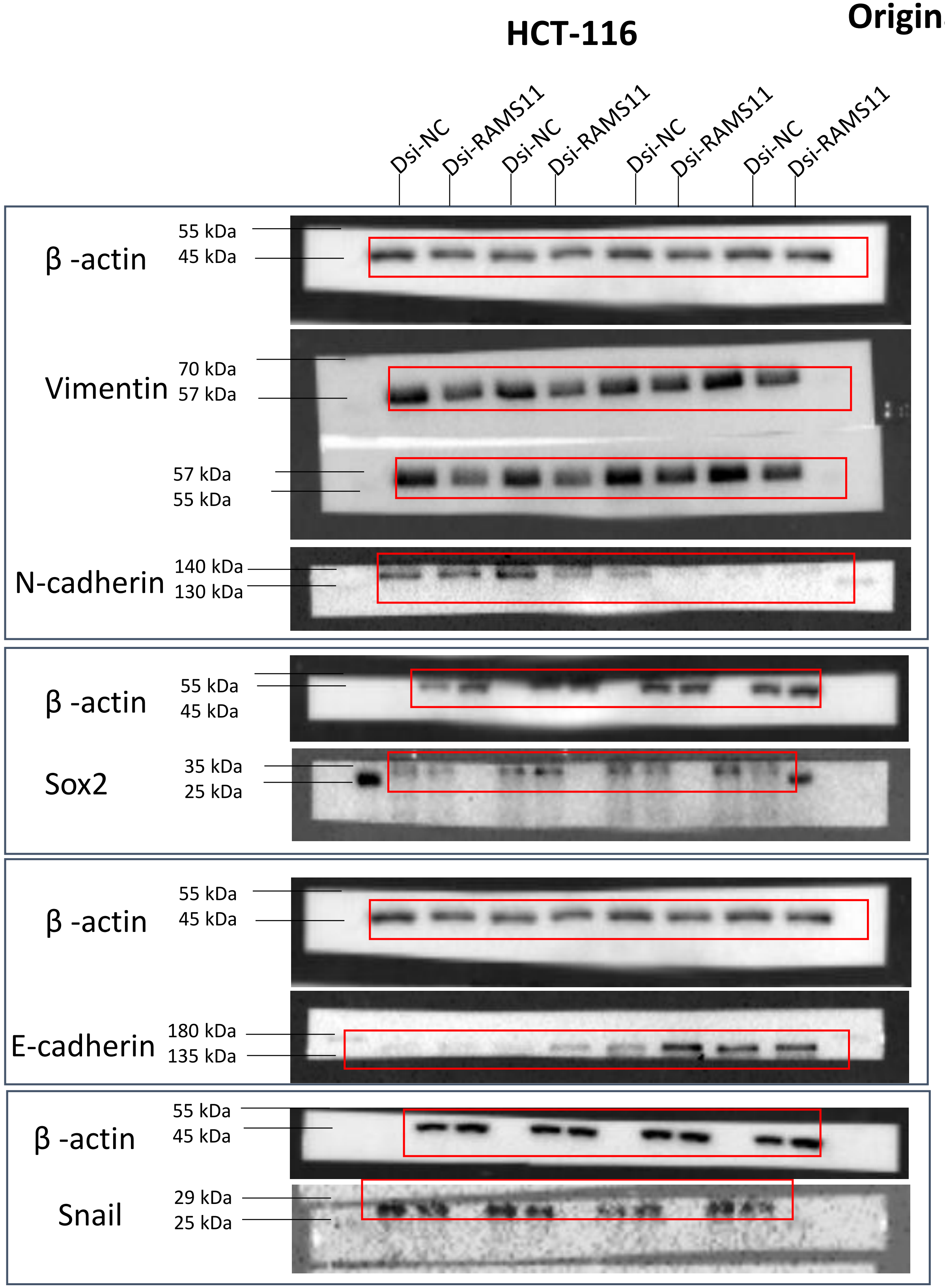

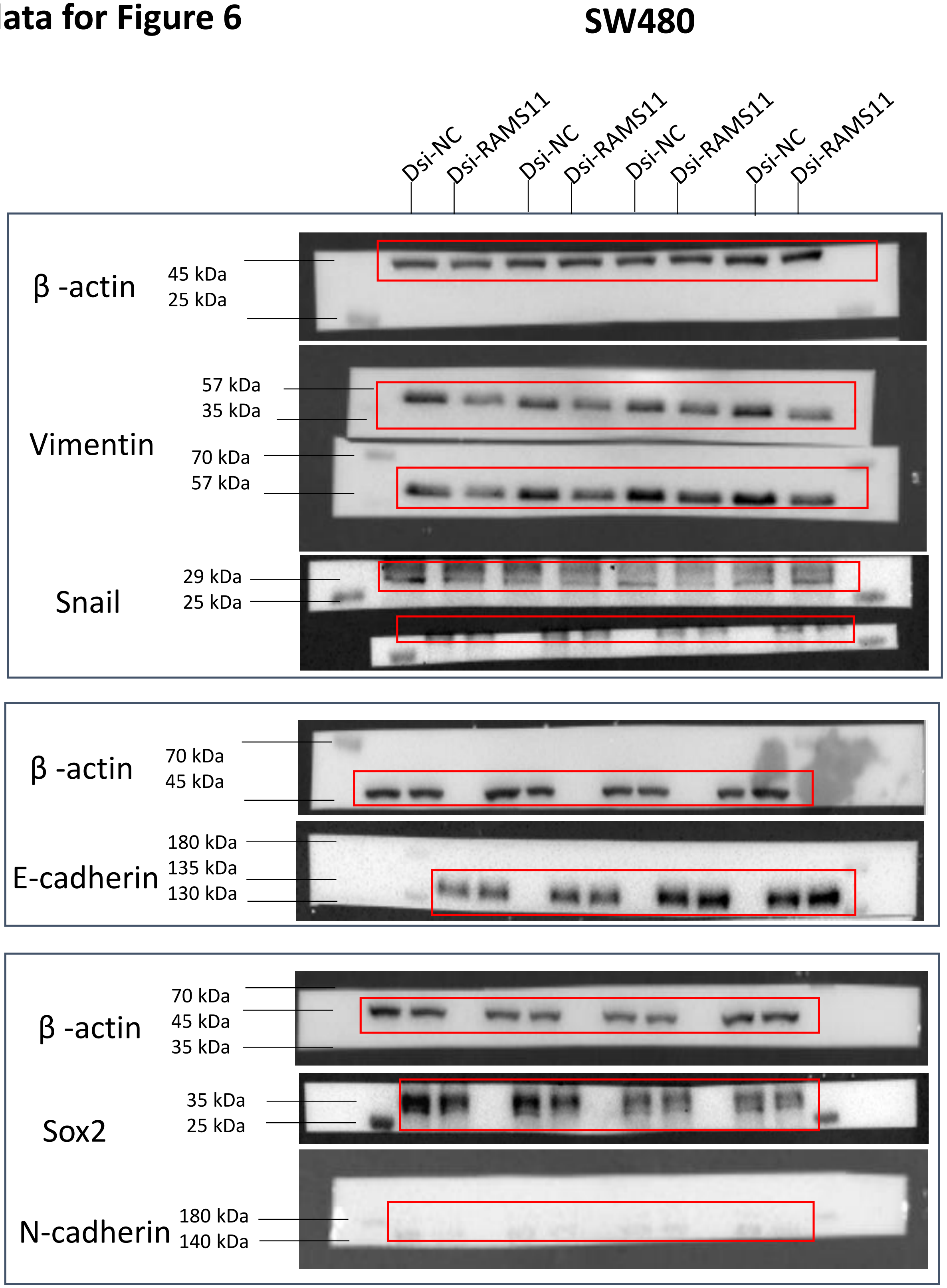

Original Western blot images

HCT-116

HCT-116

Original Western blot image for Figure 3

GAPDH

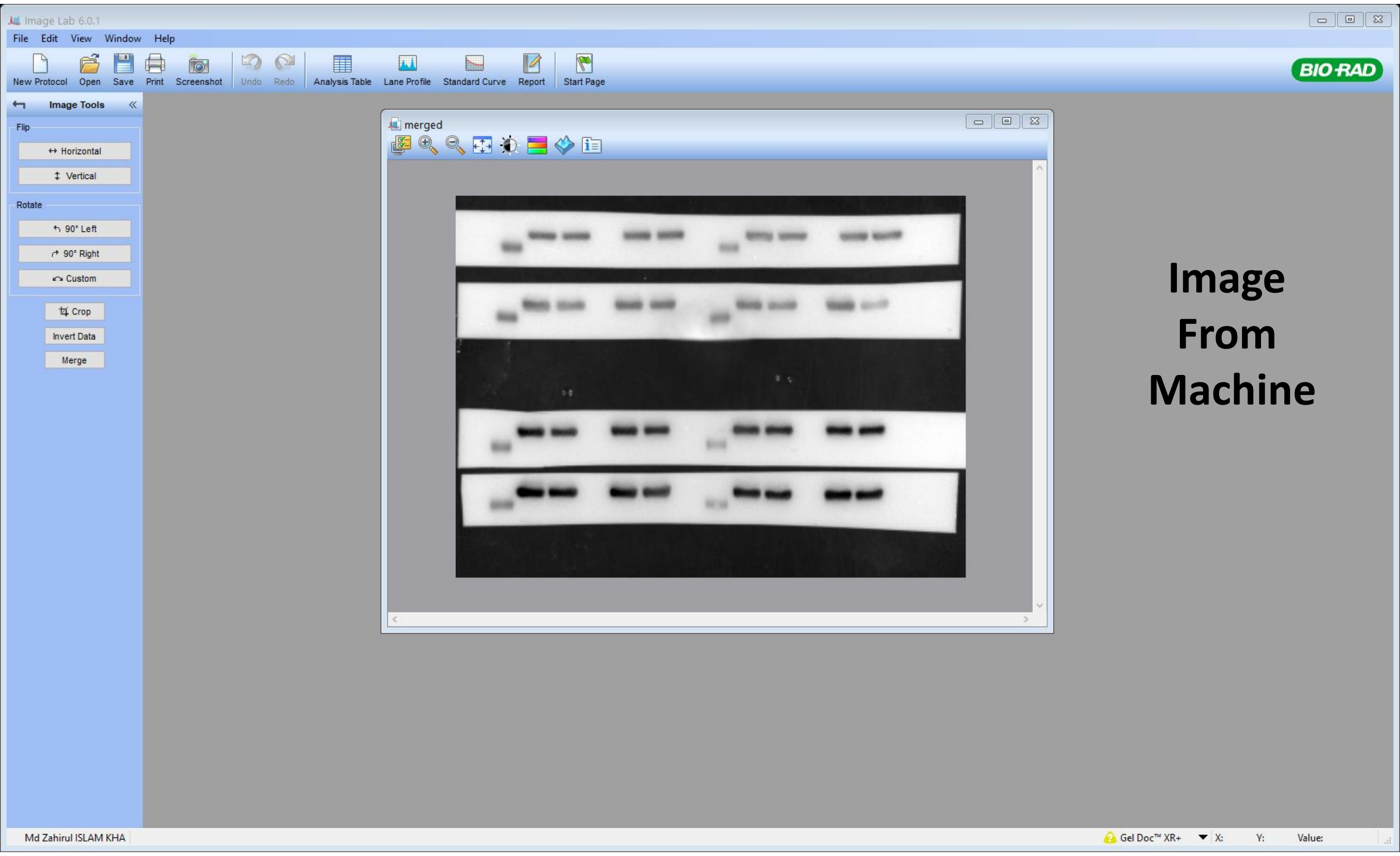

Image From Machine

Cropped Image

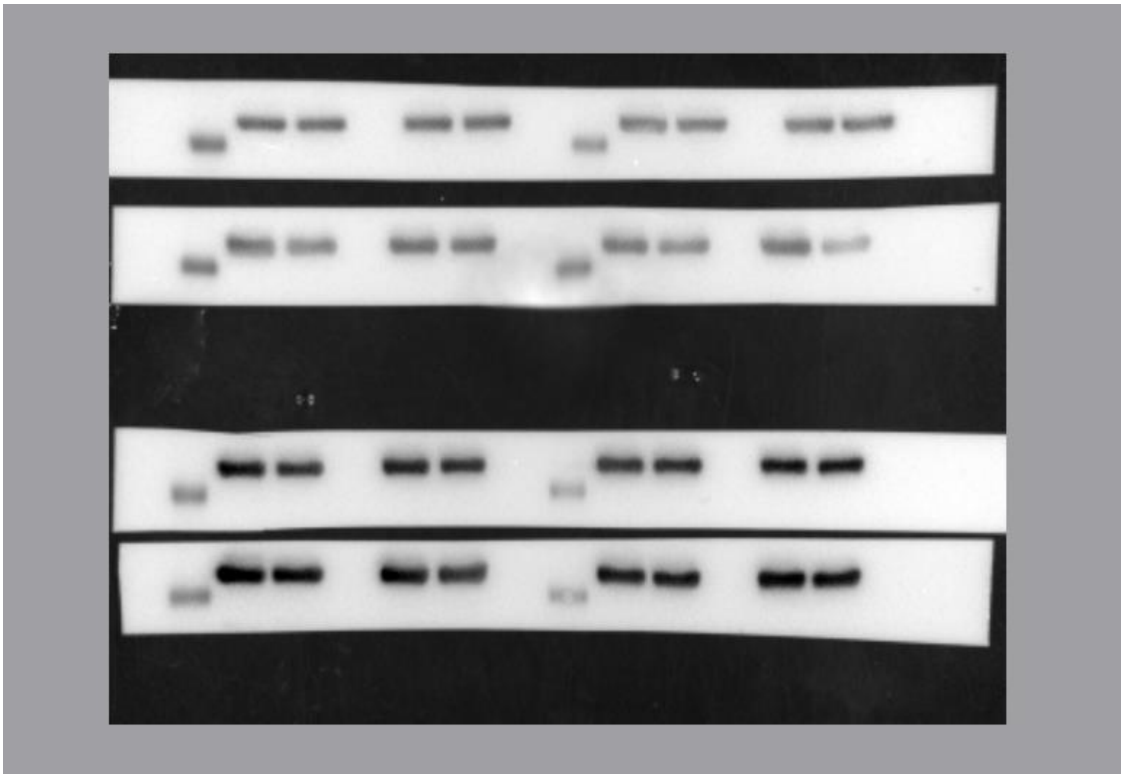

GAPDH-1

GAPDH-2

GAPDH-3

GAPDH-4

HCT-116

Original Western blot image for Figure 3

LC3B

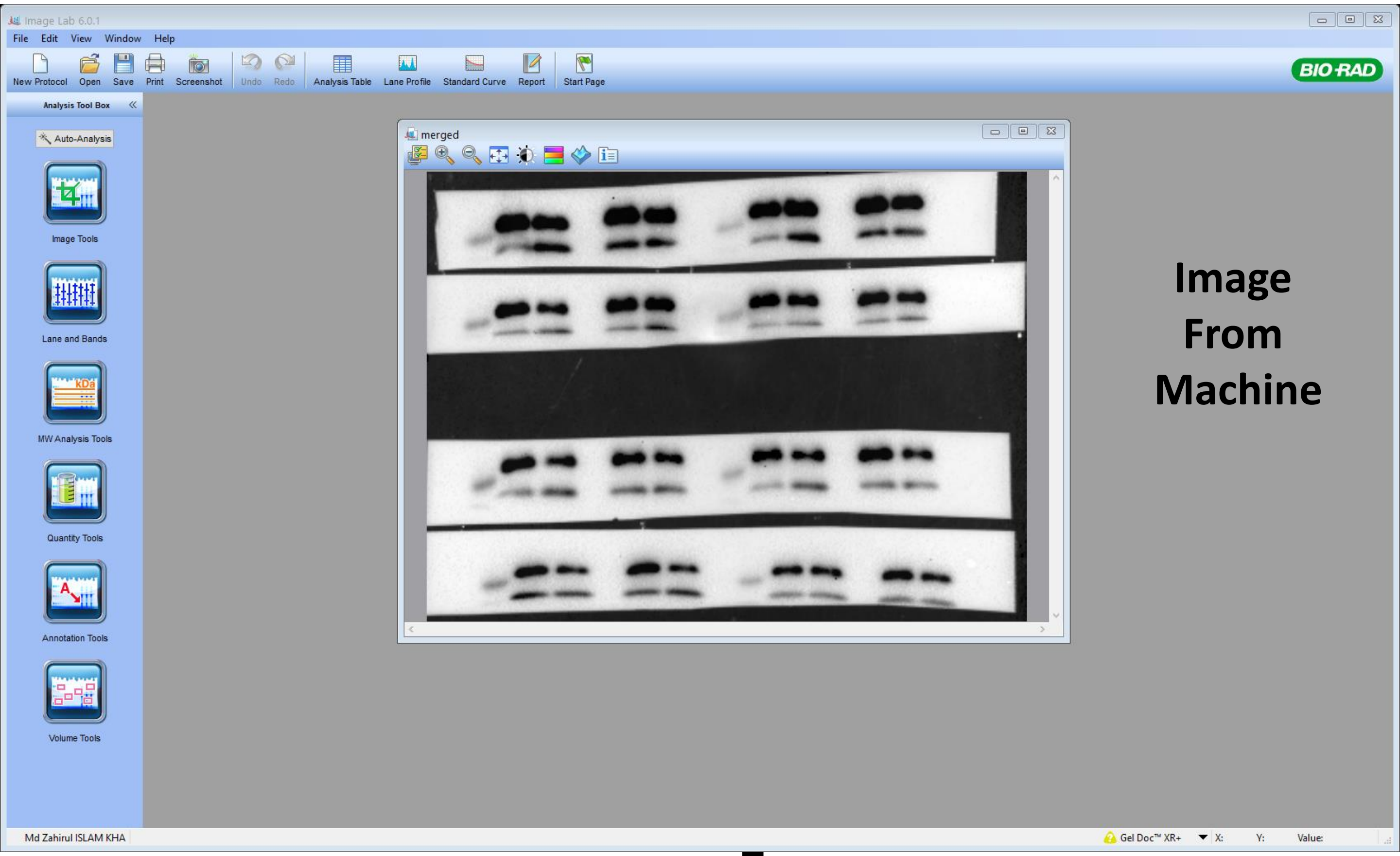

Cropped Image

LC3B-1

LC3B-2

LC3B-3

LC3B-4

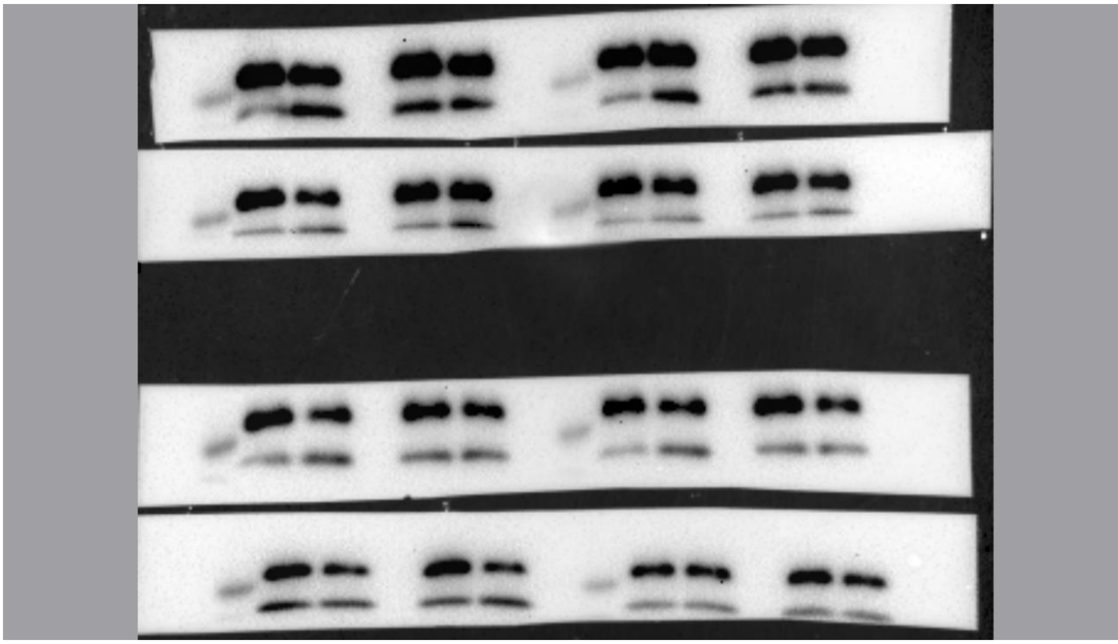

HCT-116

Original Western blot image for Figure 3

p62

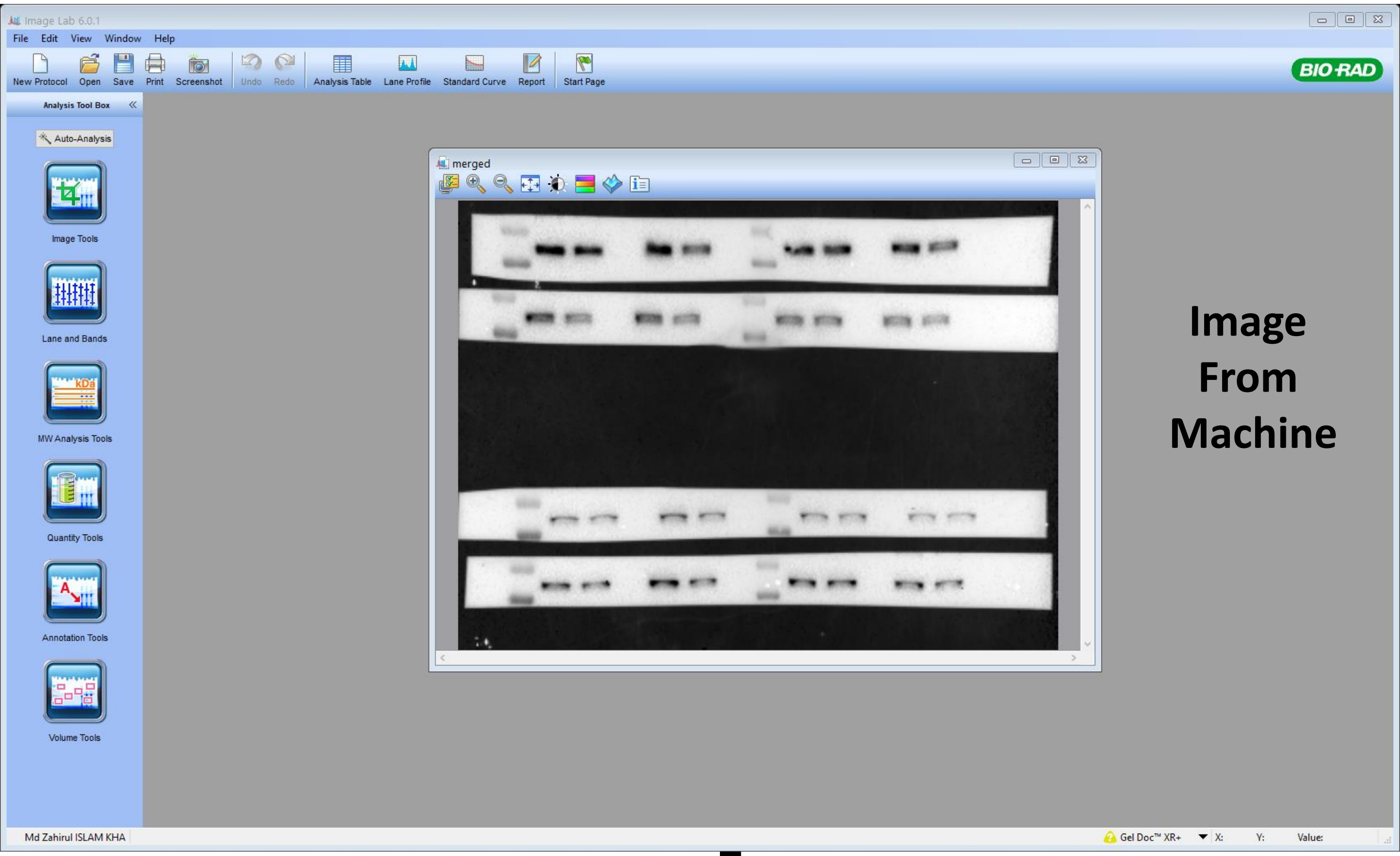

Cropped Image

p62-1

p62-2

p62-3

p62-4

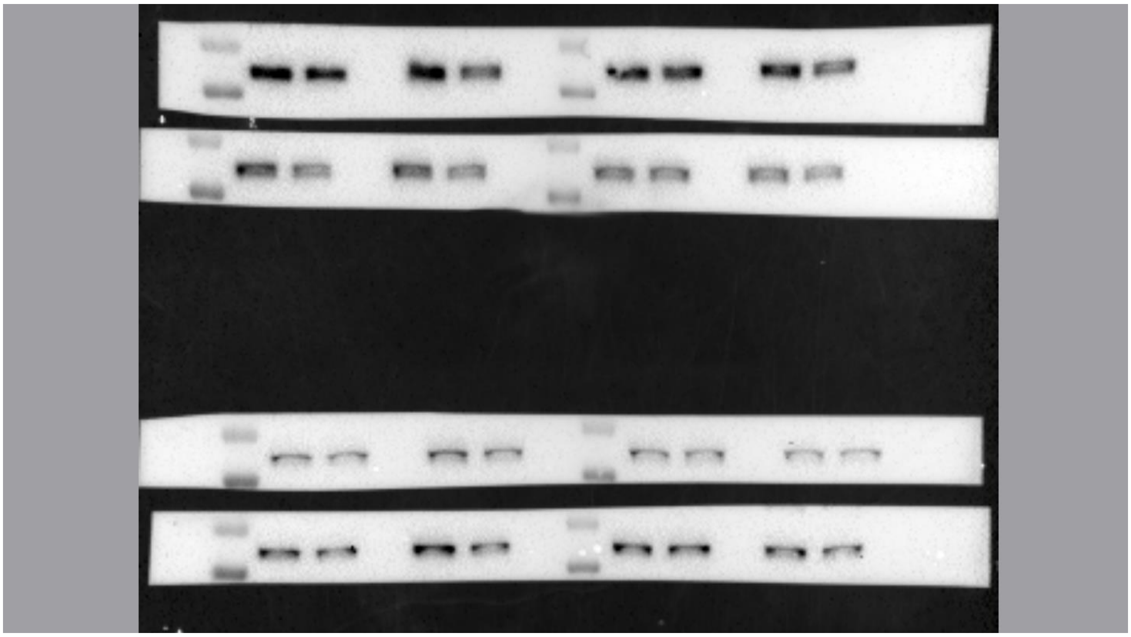

HCT-116
Original Western blot image for Figure 3
Beclin-1

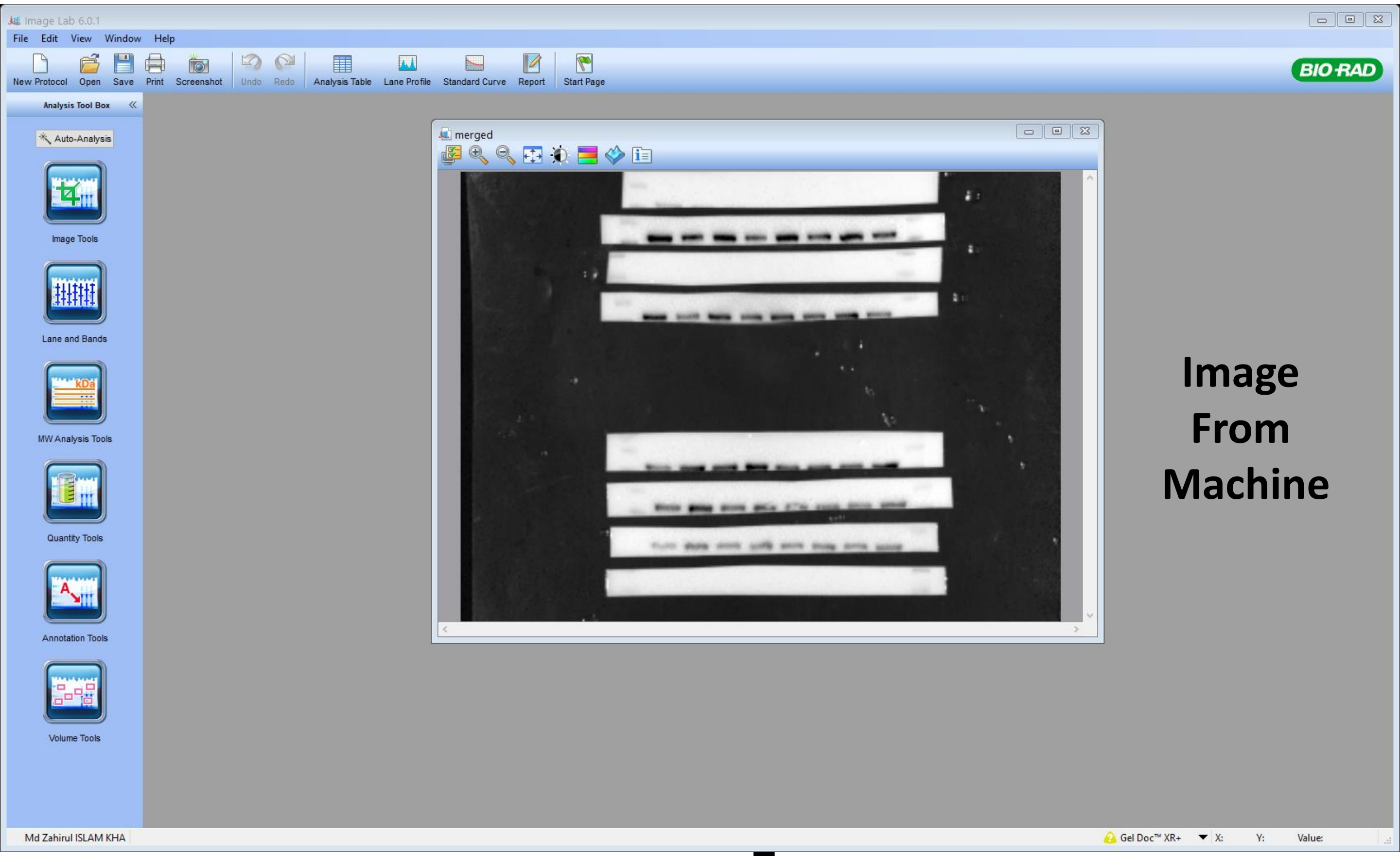

Image From Machine

Cropped Image

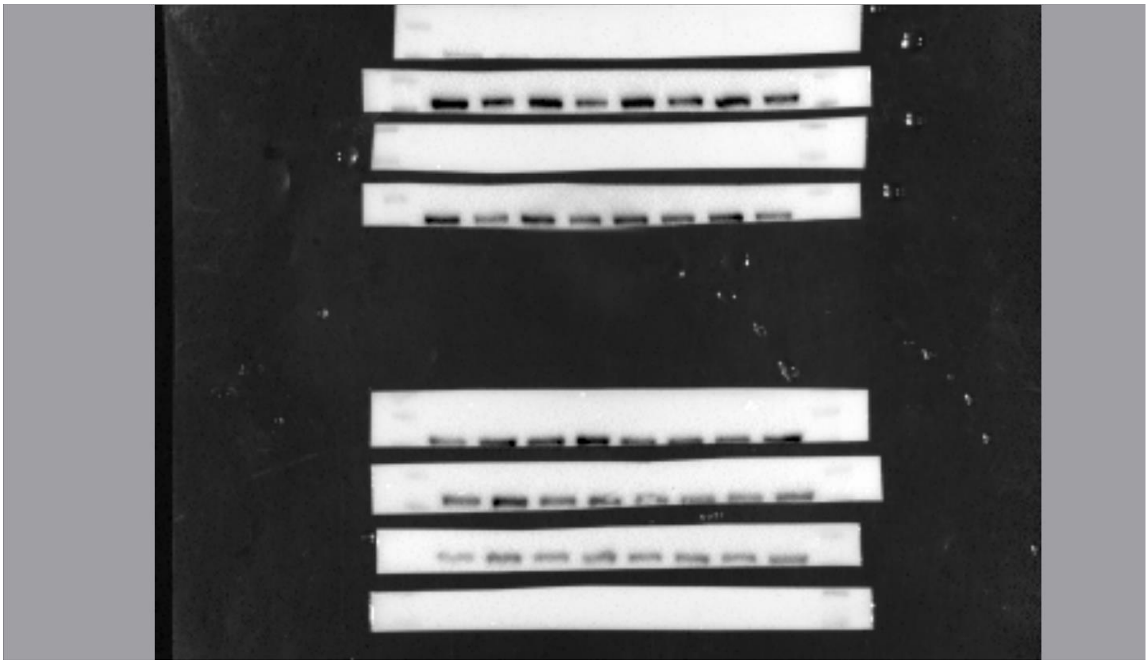

Beclin-1
Beclin-2
Beclin-3
Beclin-4

HCT-116

Original Western blot image for Figure 4

β-actin and Bxl-XL

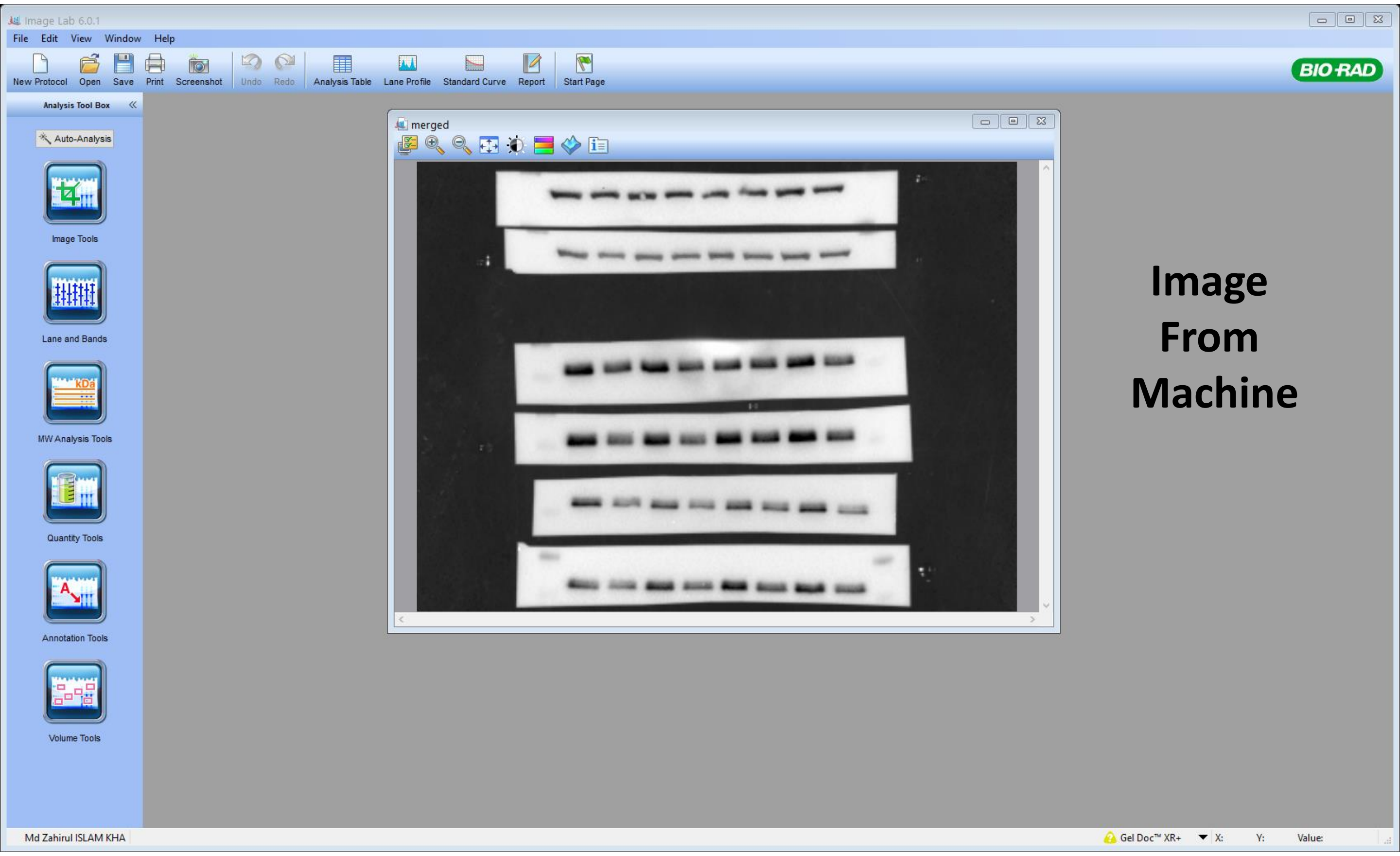

Image From Machine

Cropped Image

β-actin-1

β-actin-2

Bcl-xL-1

Bcl-xL-2

Bcl-xL-3

Bcl-xL-4

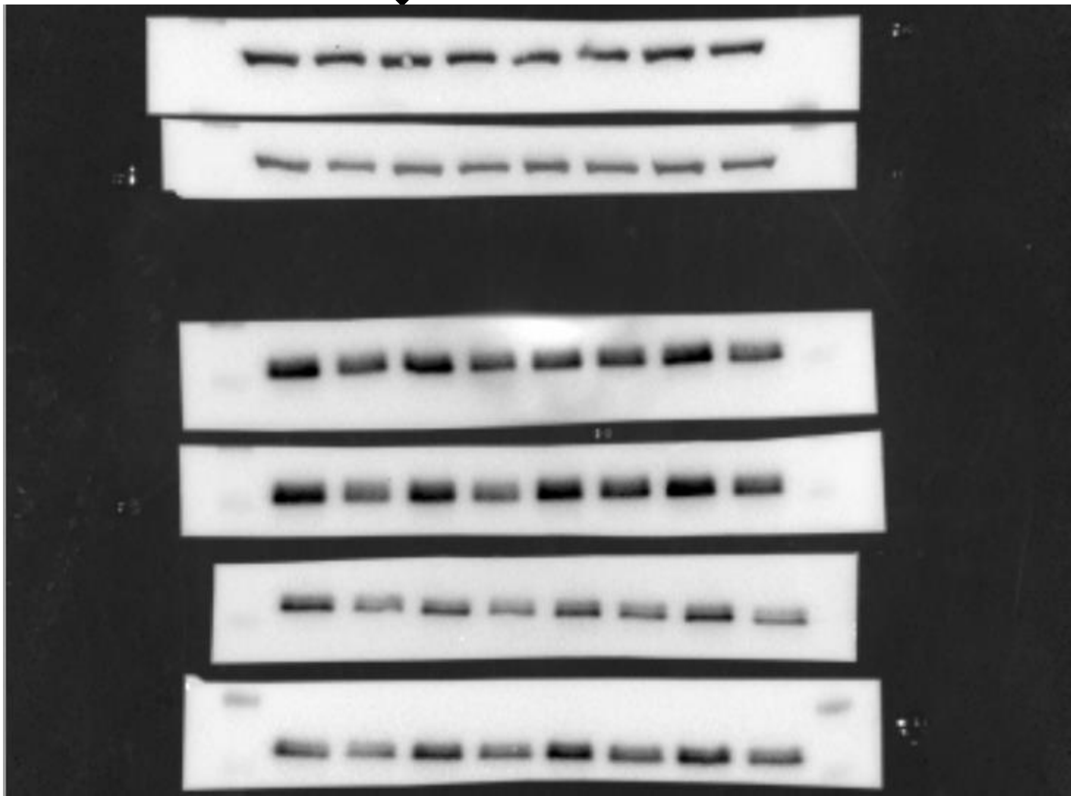

HCT-116

Original Western blot image for Figure 4

Bcl-2

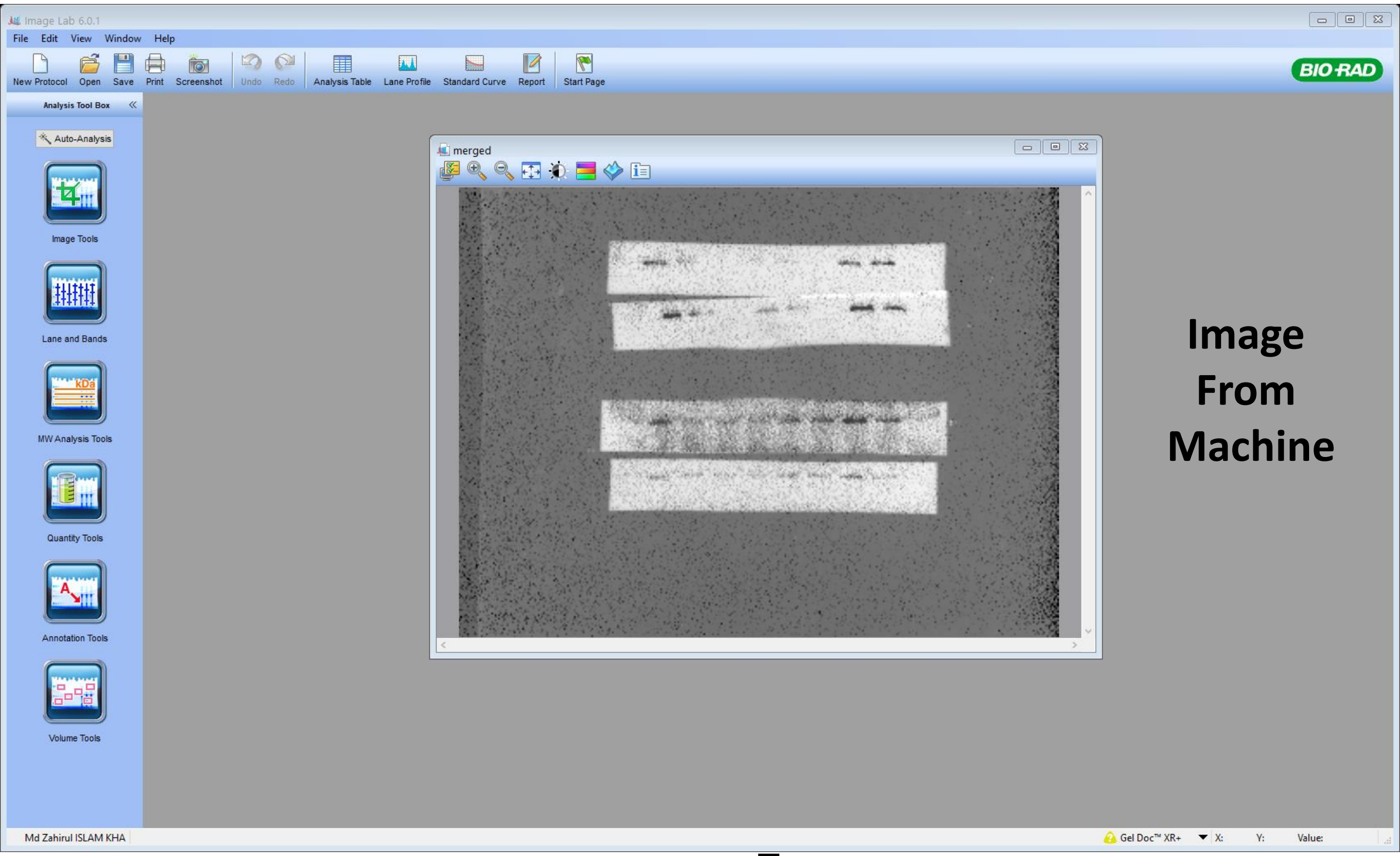

Image From Machine

Cropped Image

Bcl-2-1

Bcl-2-2

Bcl-2-3

Bcl-2-4

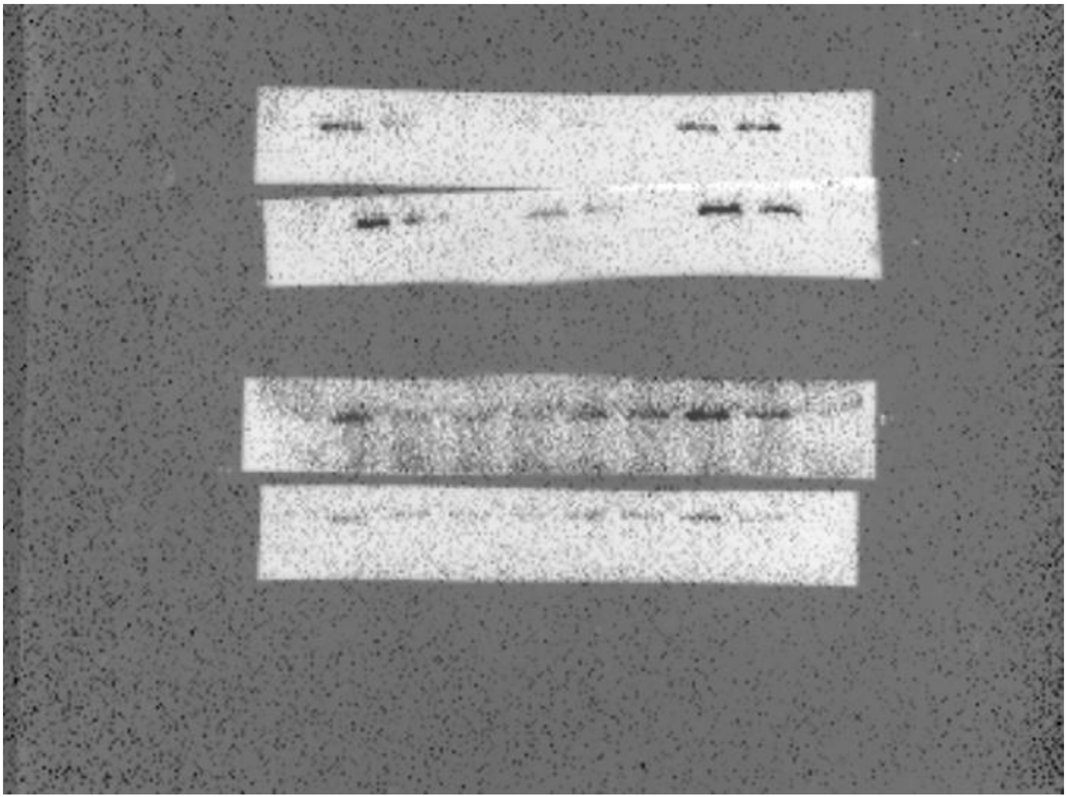

HCT-116

Original Western blot image for Figure 4
Bcl-2 and Procasepase-9

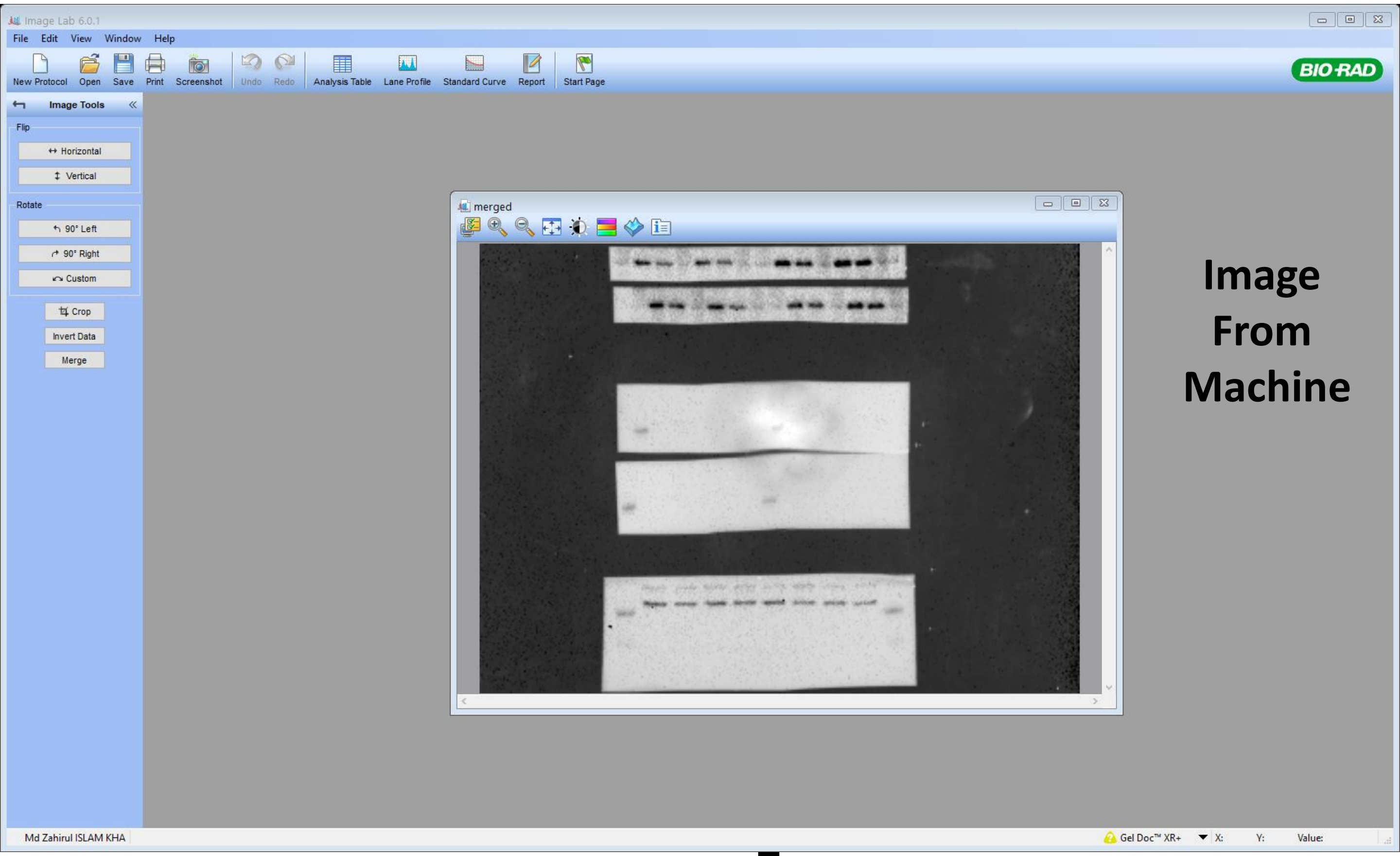

Image From Machine

Cropped Image

Bcl-2-3

Bcl-2-4

Procasepase-9-1

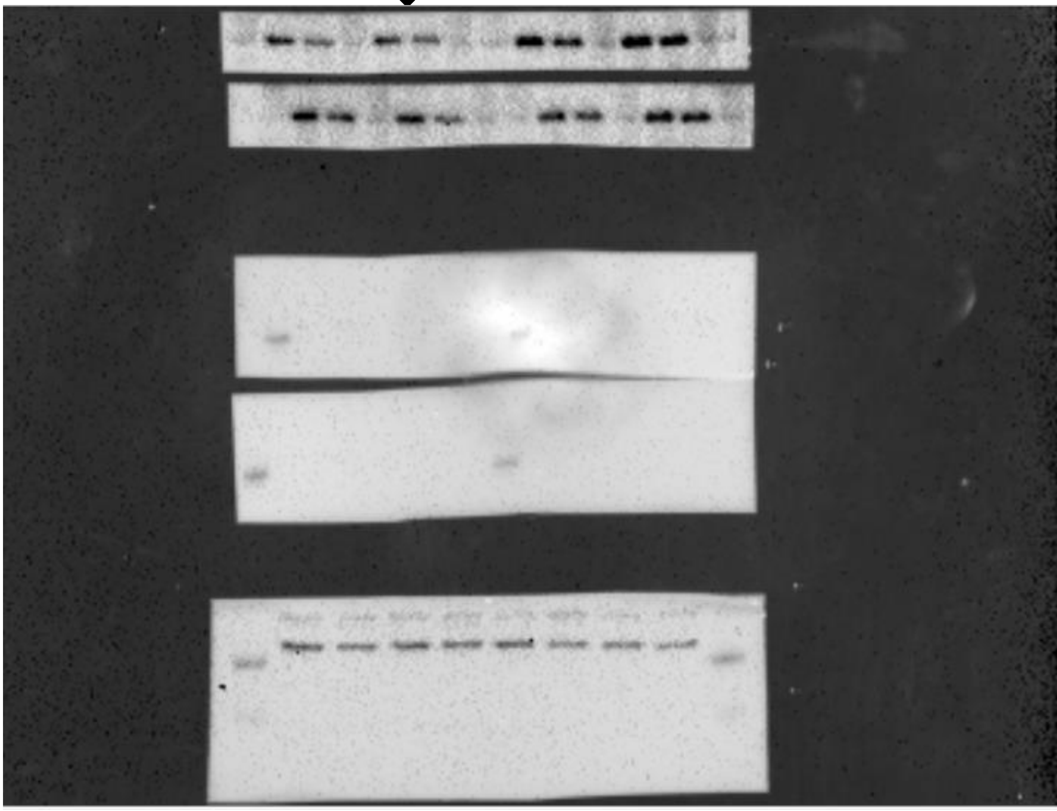

HCT-116
Original Western blot image for Figure 4
Procaspase-9

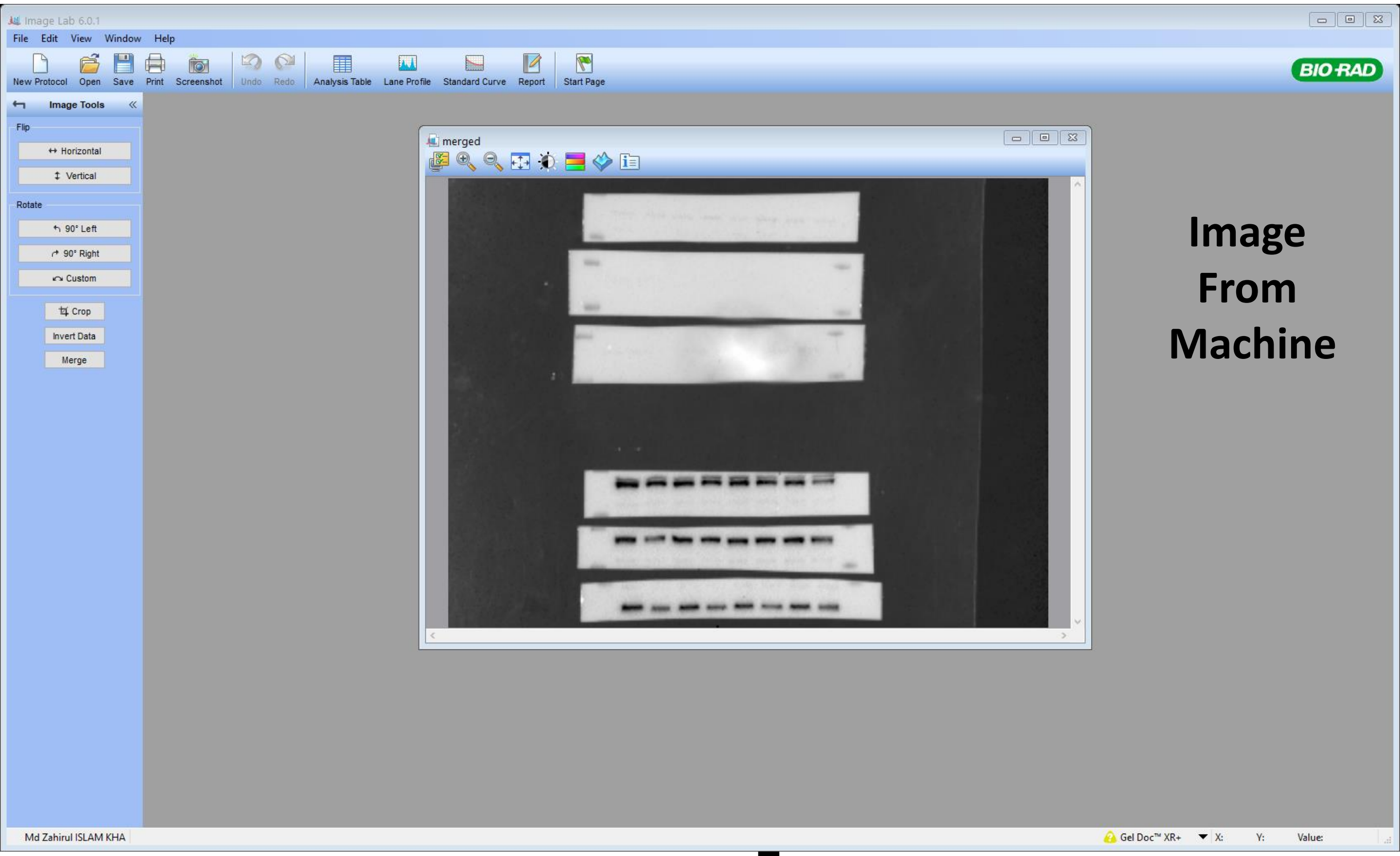

Image From Machine

Cropped Image

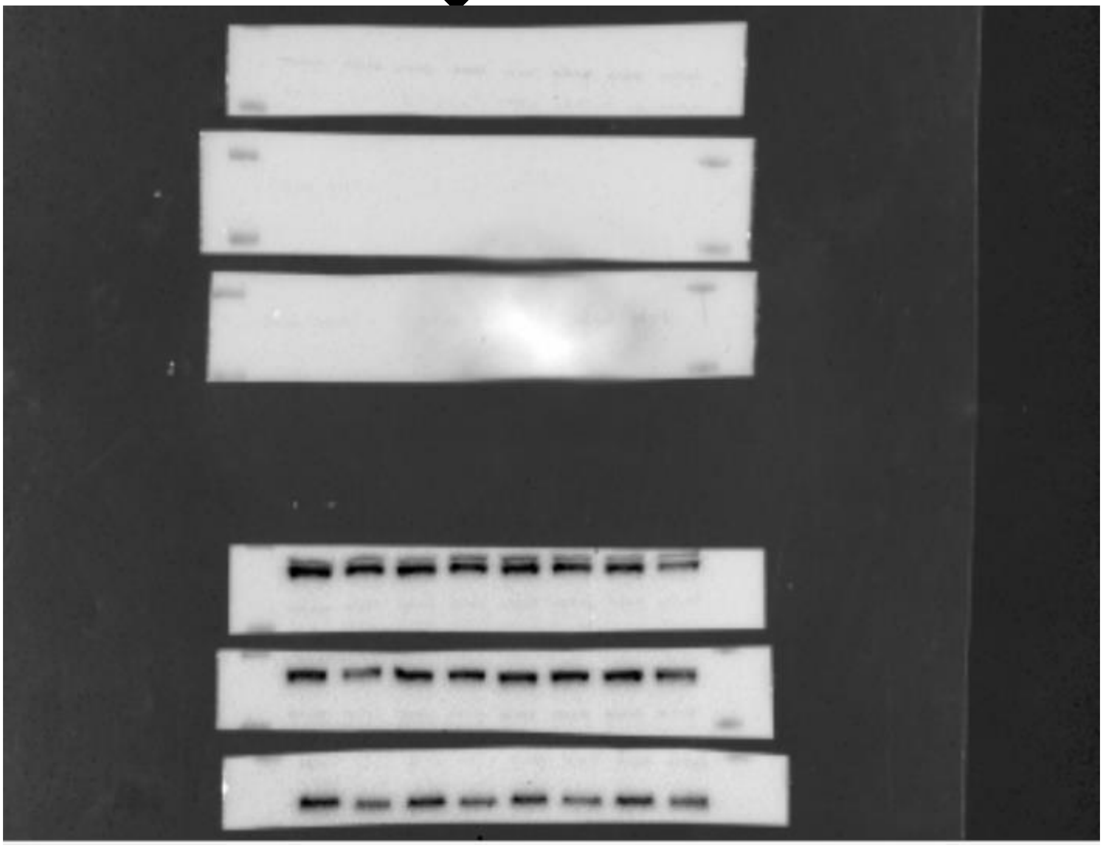

Procaspase-9-2

Procaspase-9-3

Procaspase-9-3

HCT-116
Original Western blot image for Figure 5
β-actin

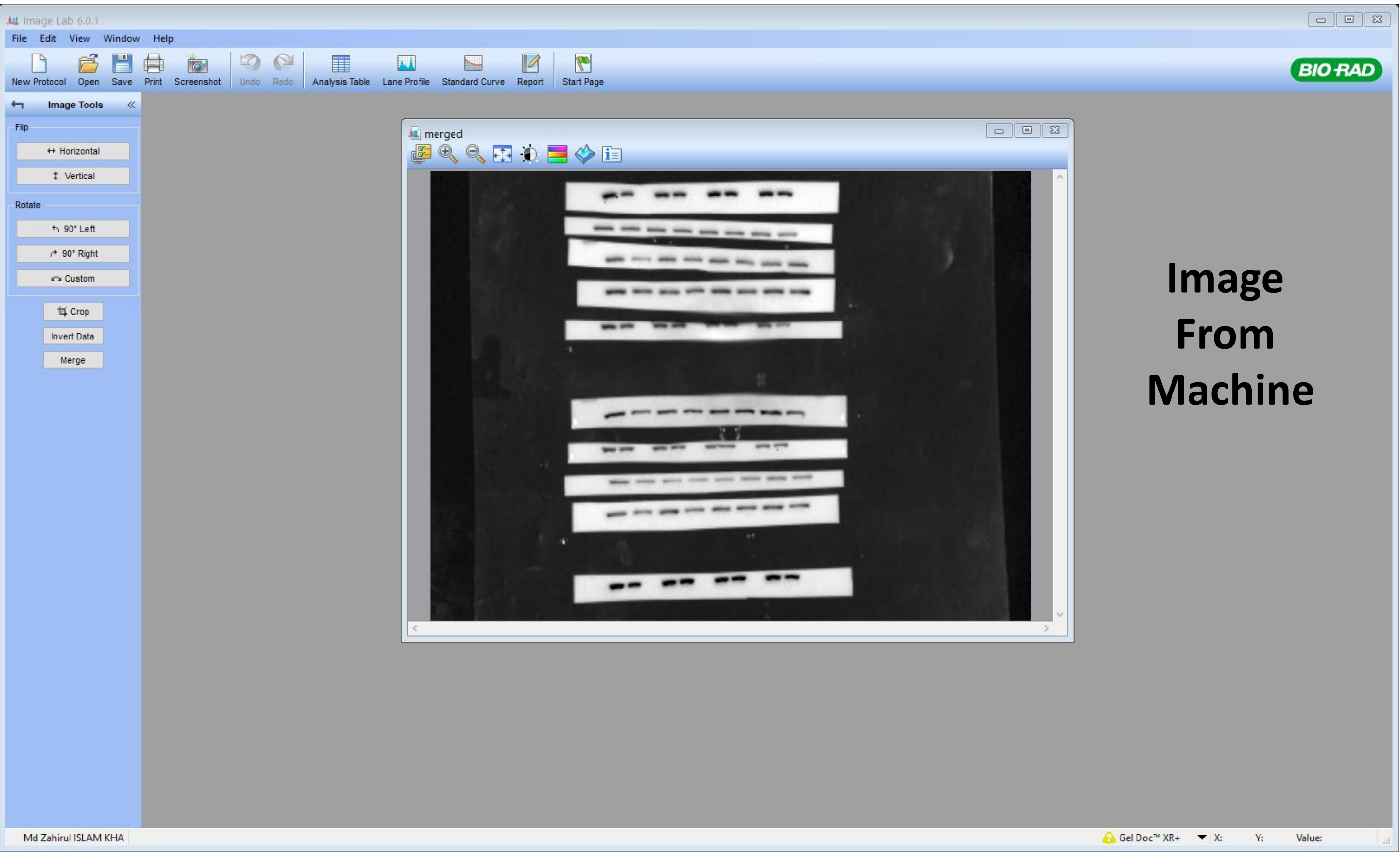

Image From Machine

Cropped Image

β-actin-1
β-actin-2
β-actin-3
β-actin-4
β-actin-5

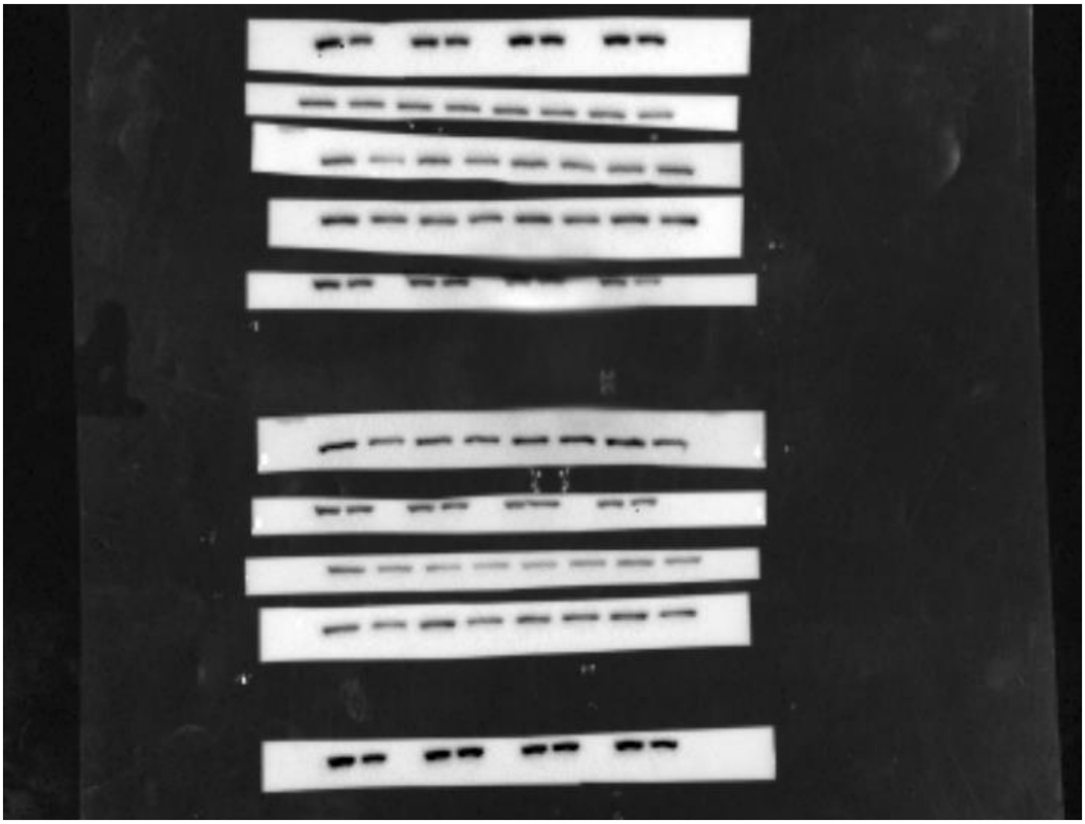

HCT-116
Original Western blot image for Figure 5
p-AKT,AKT, p-AMPK,AMPK

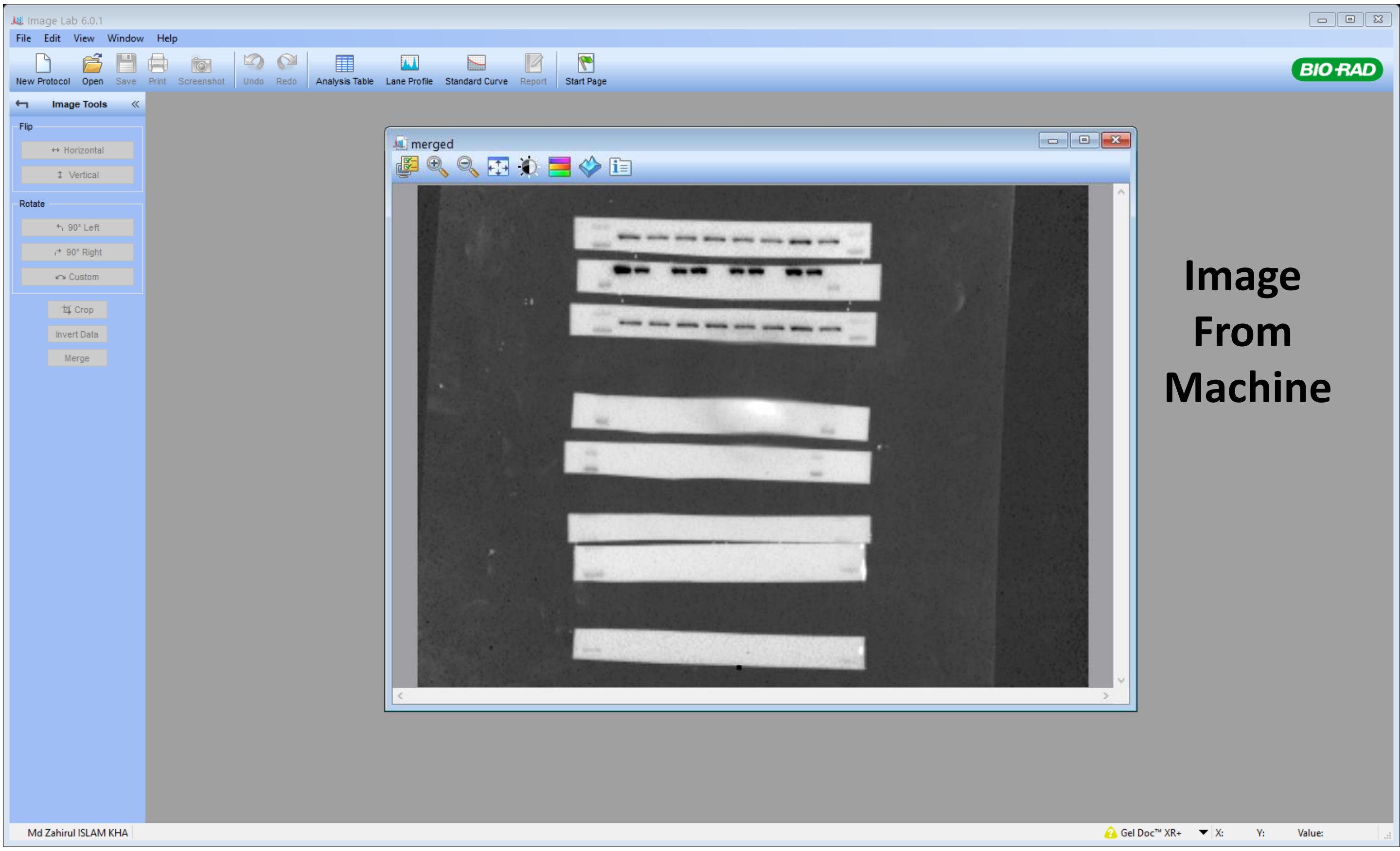

Cropped
Image

p-AKT-1
p-AMPK-1
AMPK-1
p-AKT-2
p-AMPK-2
AKT-1
AKT-2
AMPK-2

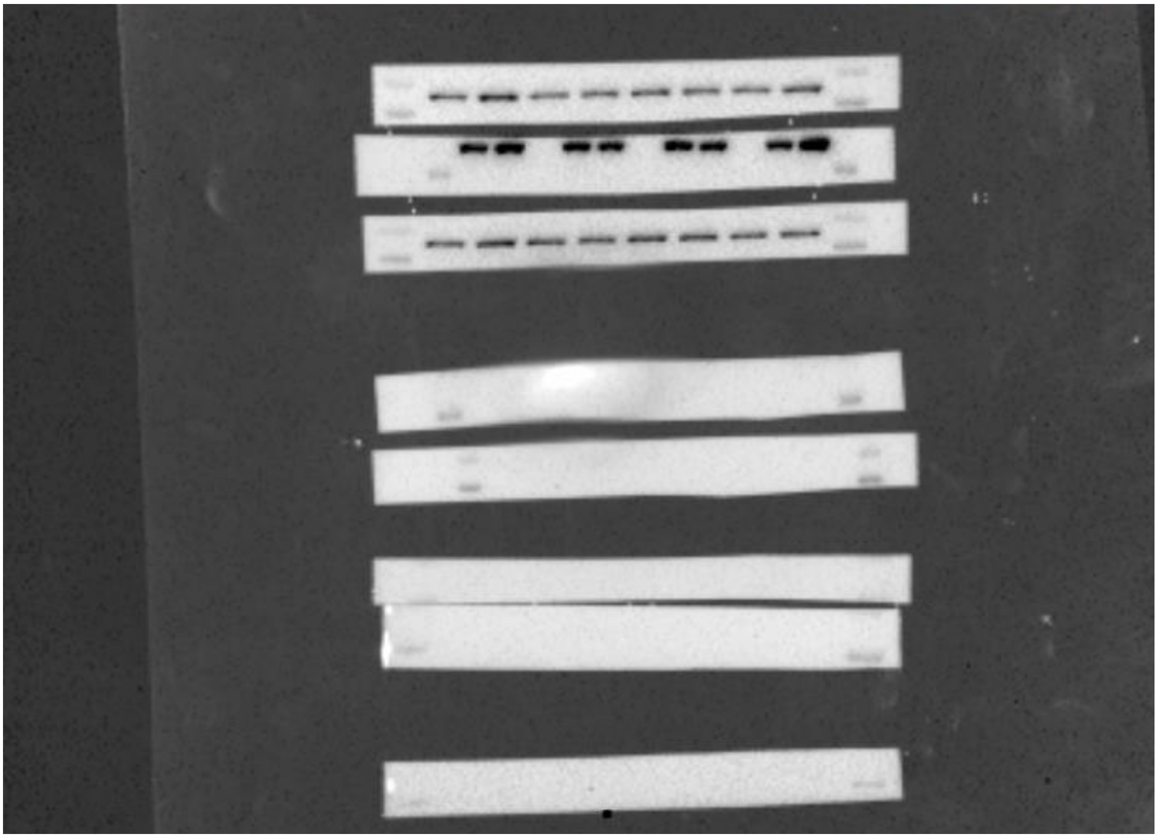

HCT-116
Original Western blot image for Figure 5
p-AKT, AKT, p-AMPK

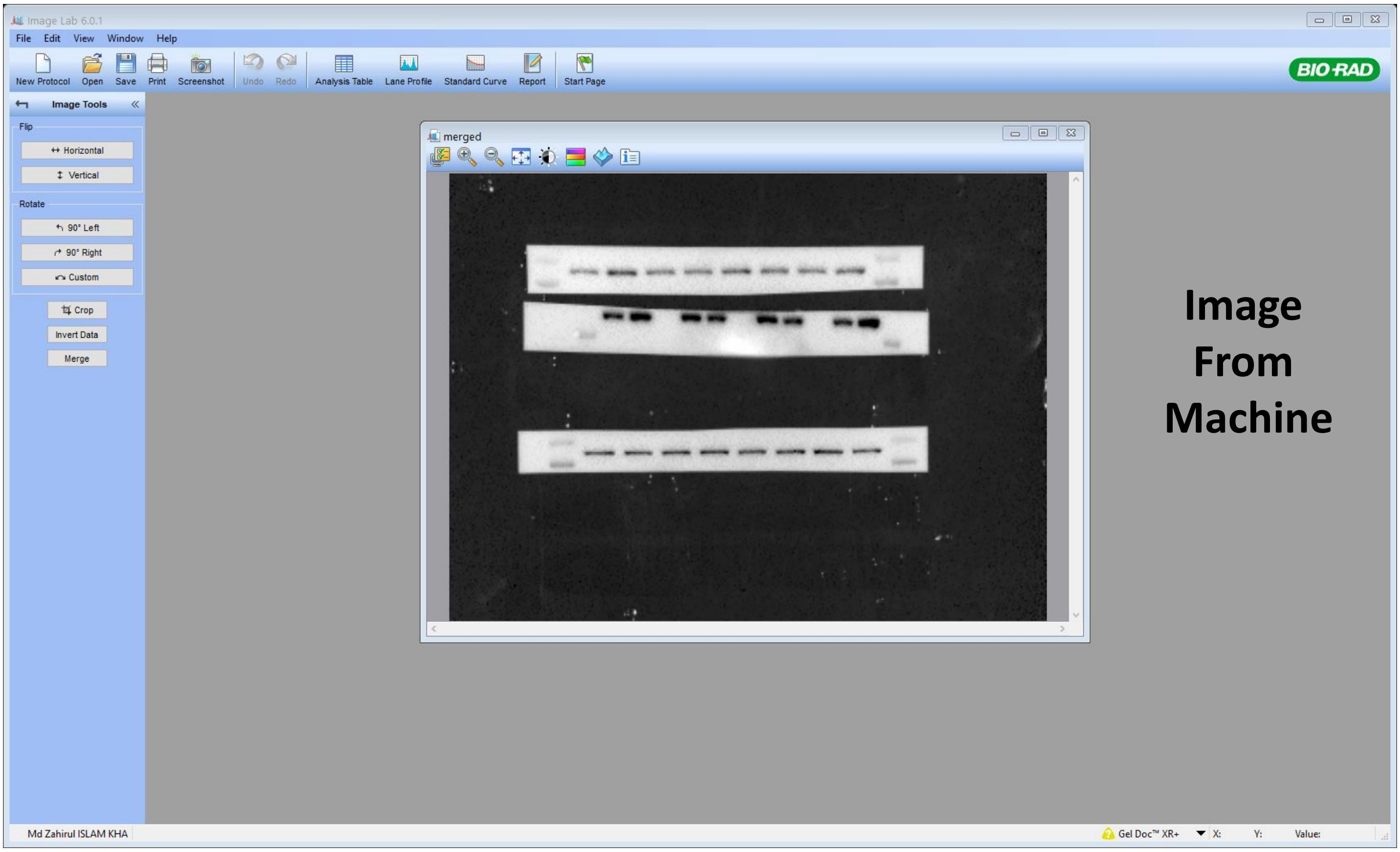

Image From Machine

Cropped Image

p-AKT-3

p-AMPK-3

AKT-3

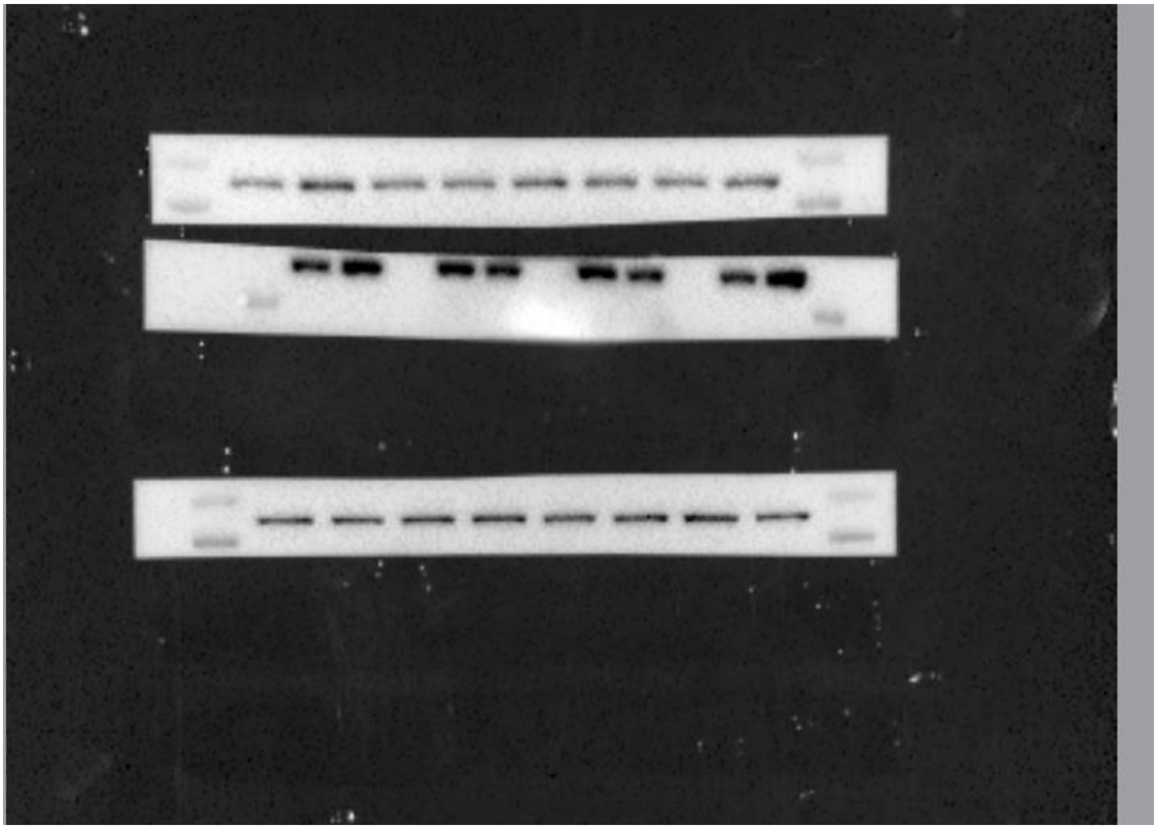

HCT-116

Original Western blot image for Figure 5

p-mTOR, mTOR

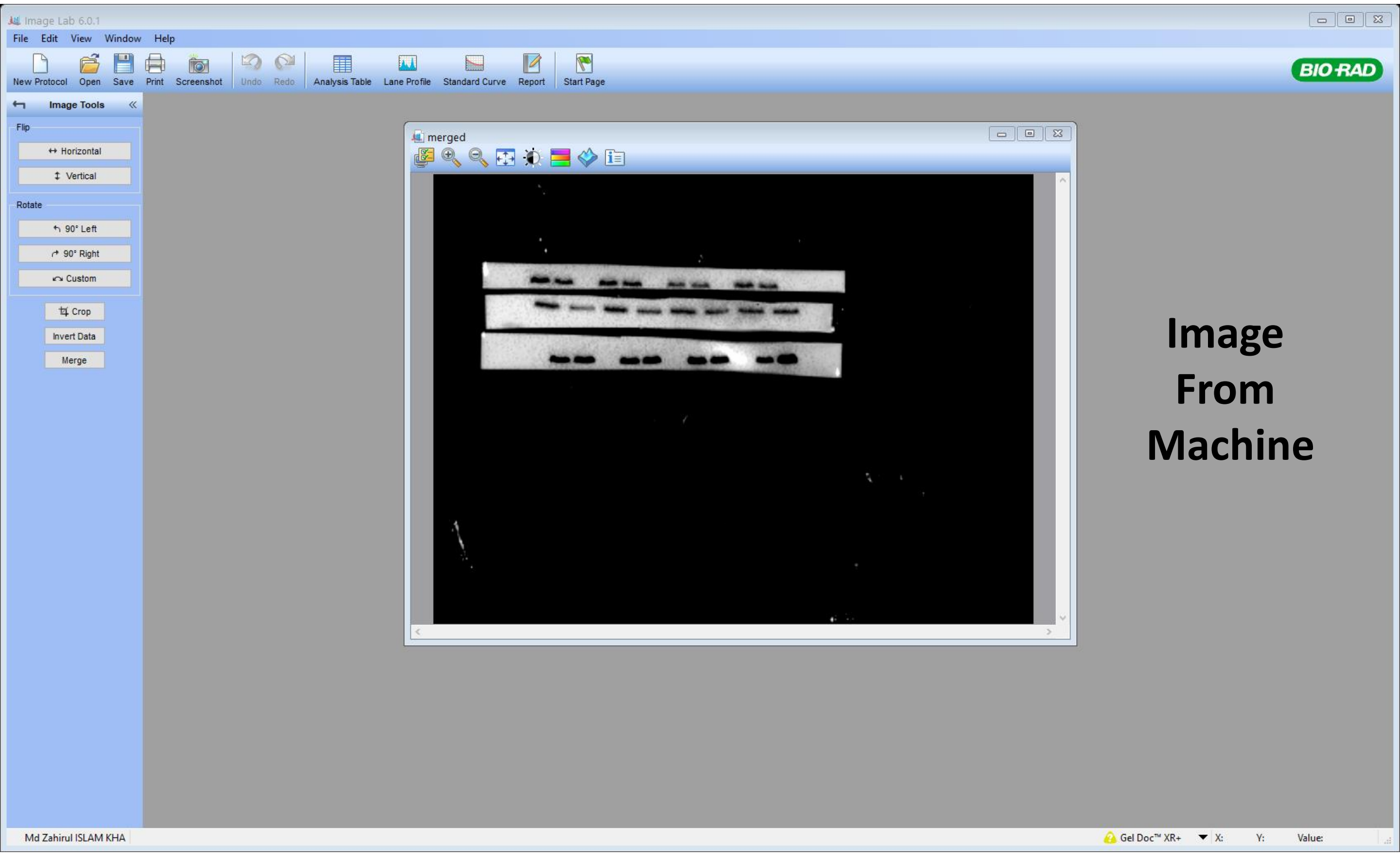

Image From Machine

Cropped Image

mTOR-1

p-mTOR-1

mTOR-2

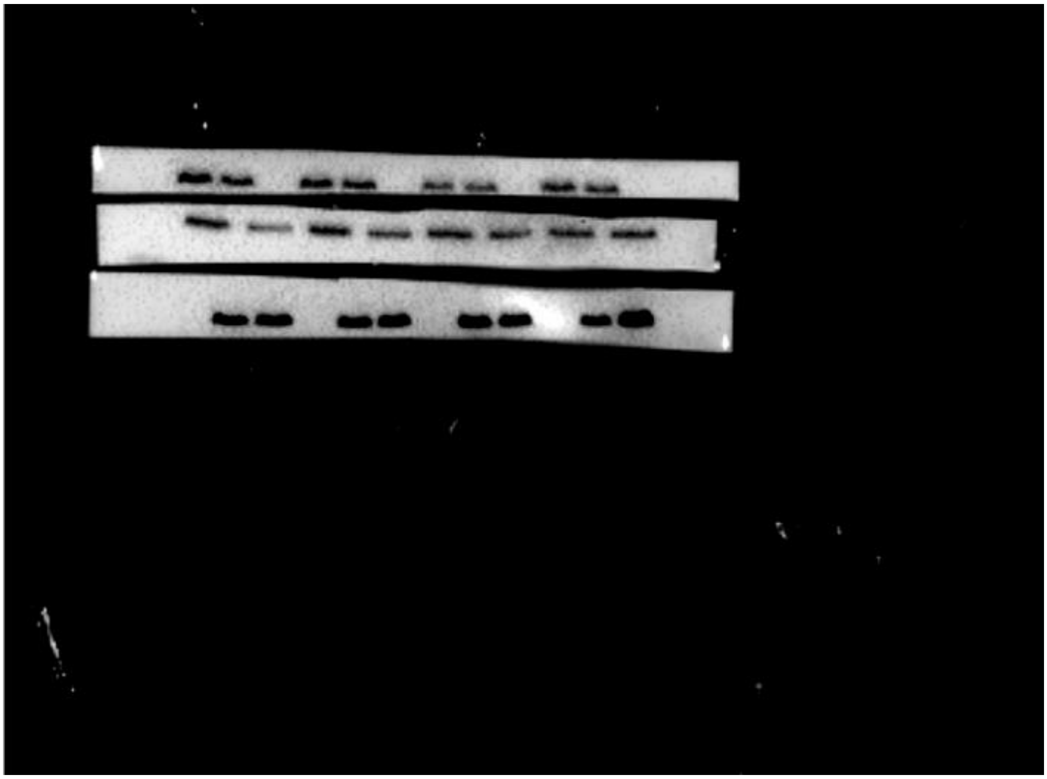

HCT-116
Original Western blot image for Figure 5
p-mTOR, mTOR

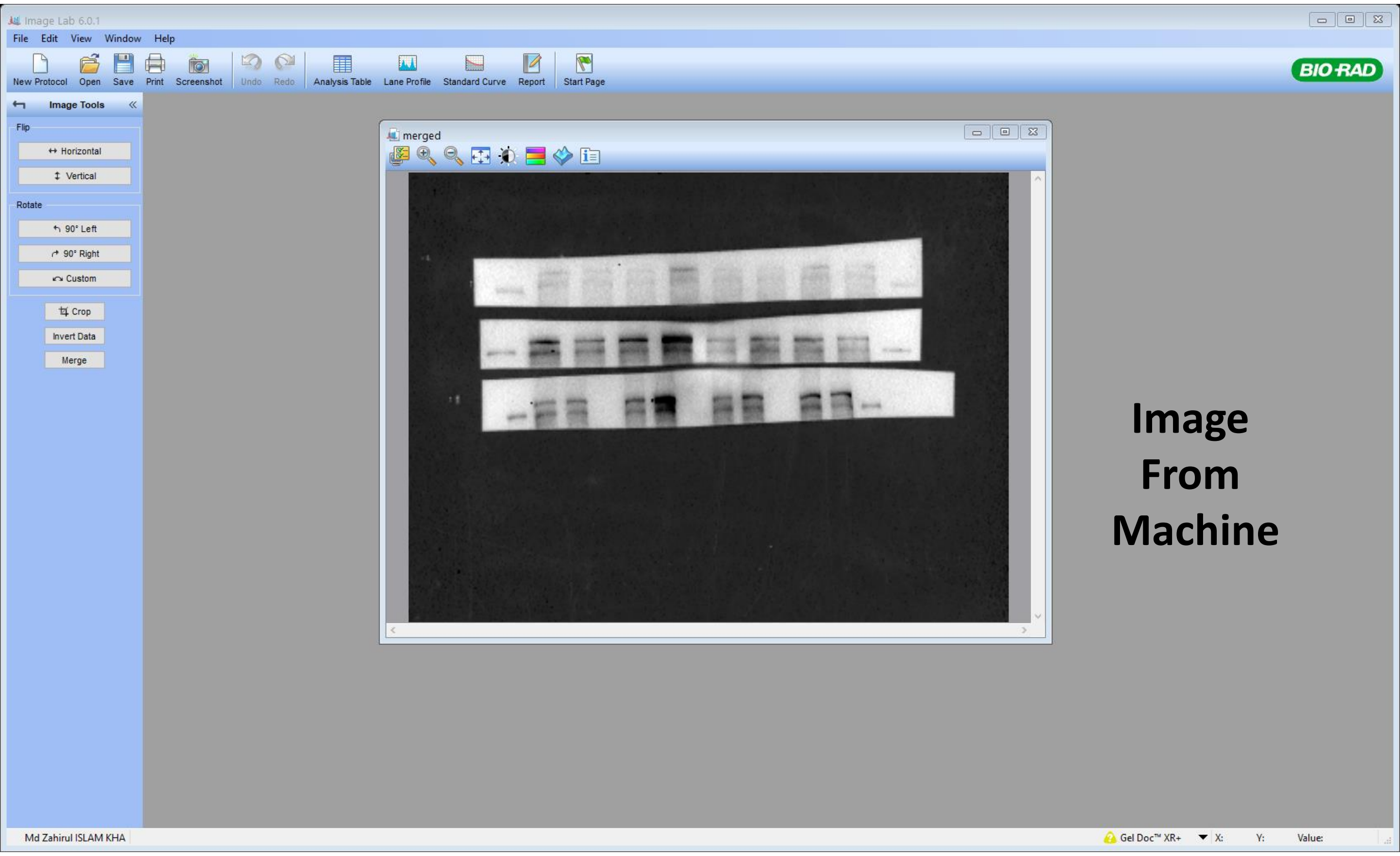

Image From Machine

Cropped Image

mTOR-3

p-mTOR-2

p-mTOR-3

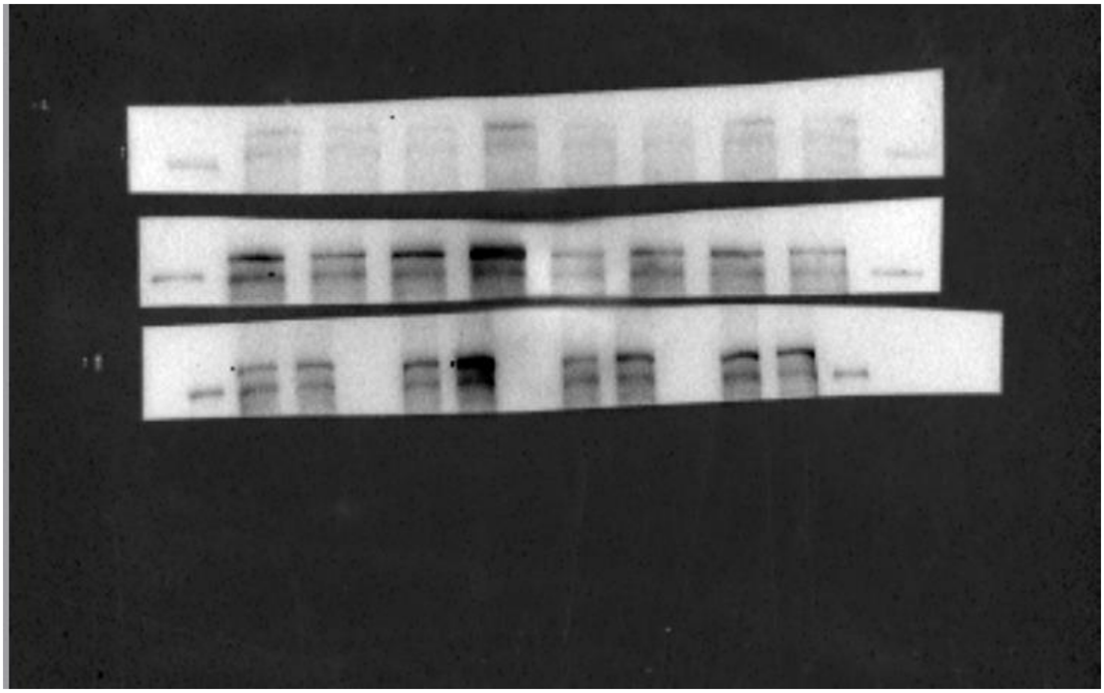

HCT-116
Original Western blot image for Figure 6
β-actin

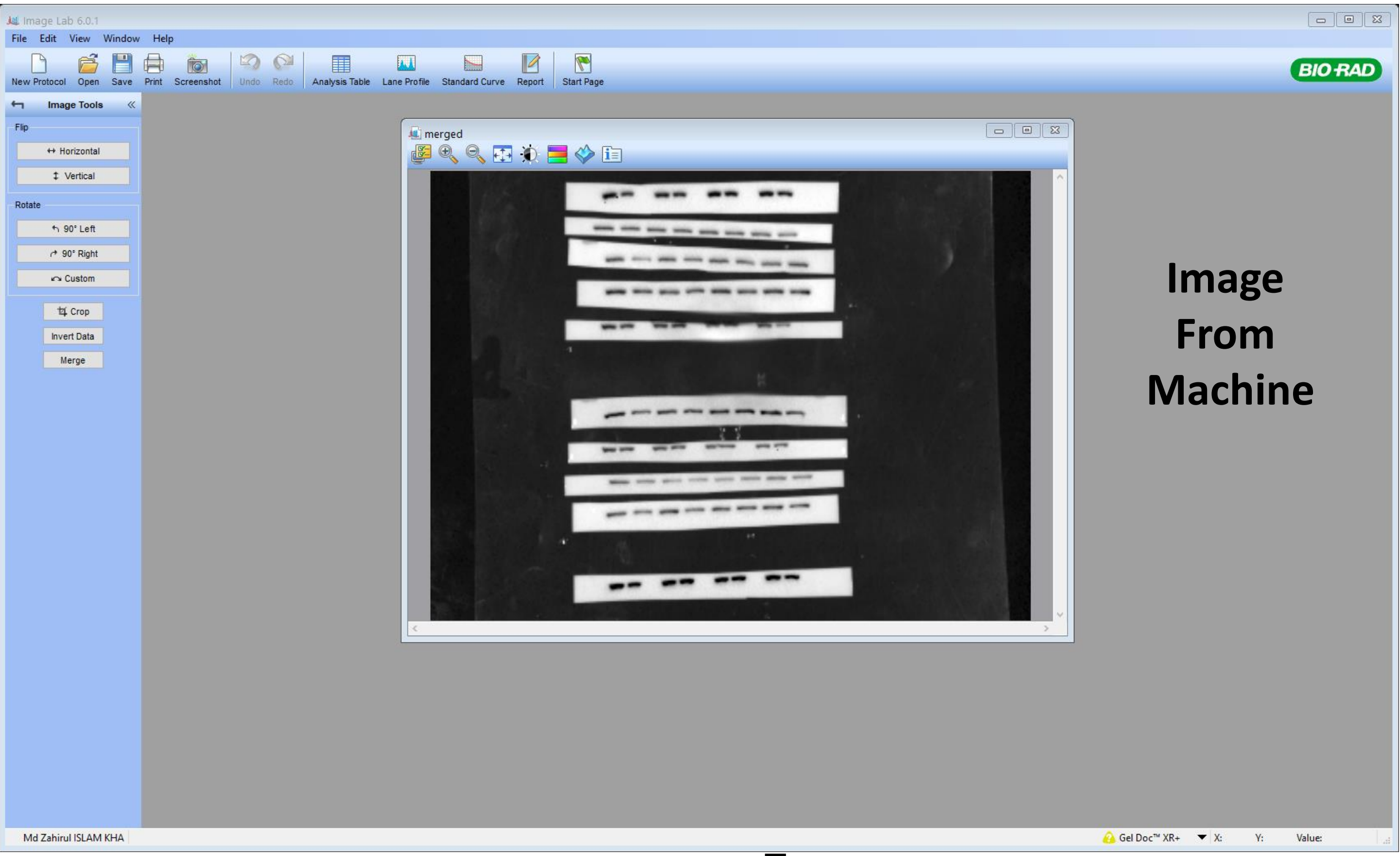

Image From Machine

Cropped Image

β-actin-1
β-actin-2
β-actin-3
β-actin-4

β-actin-5

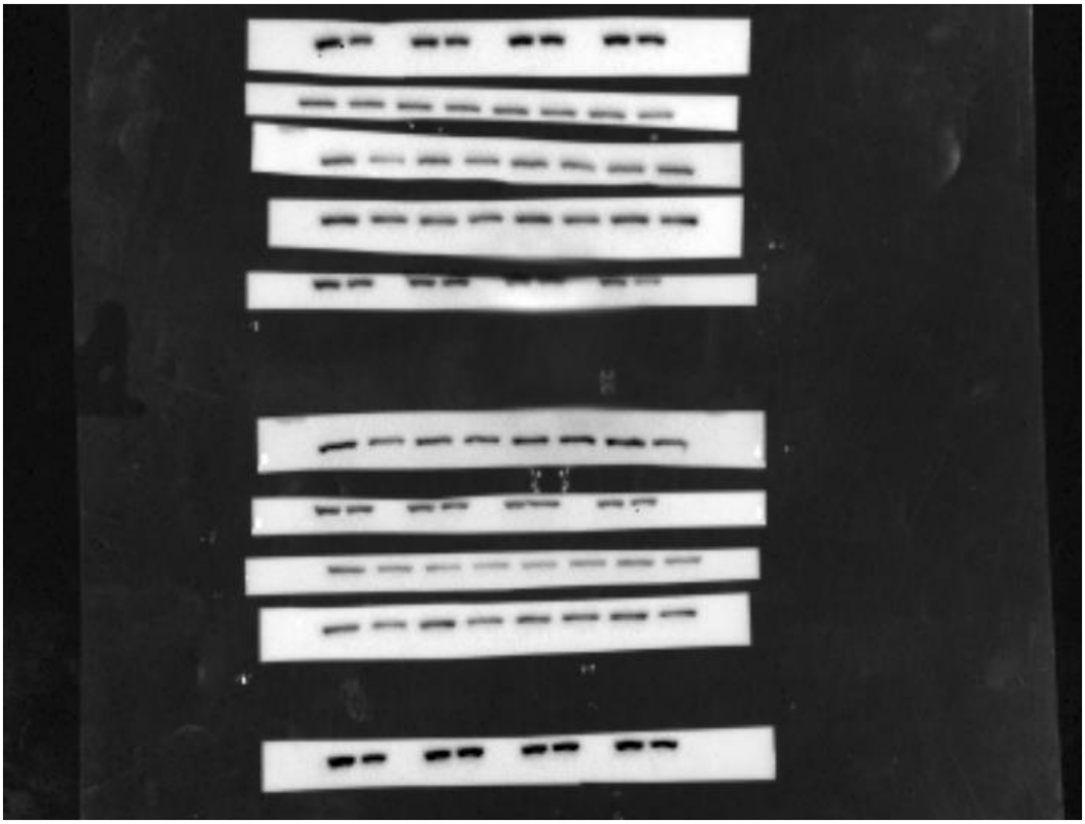

HCT-116
Original Western blot image for Figure 6
N-cadherin, E-cadherin

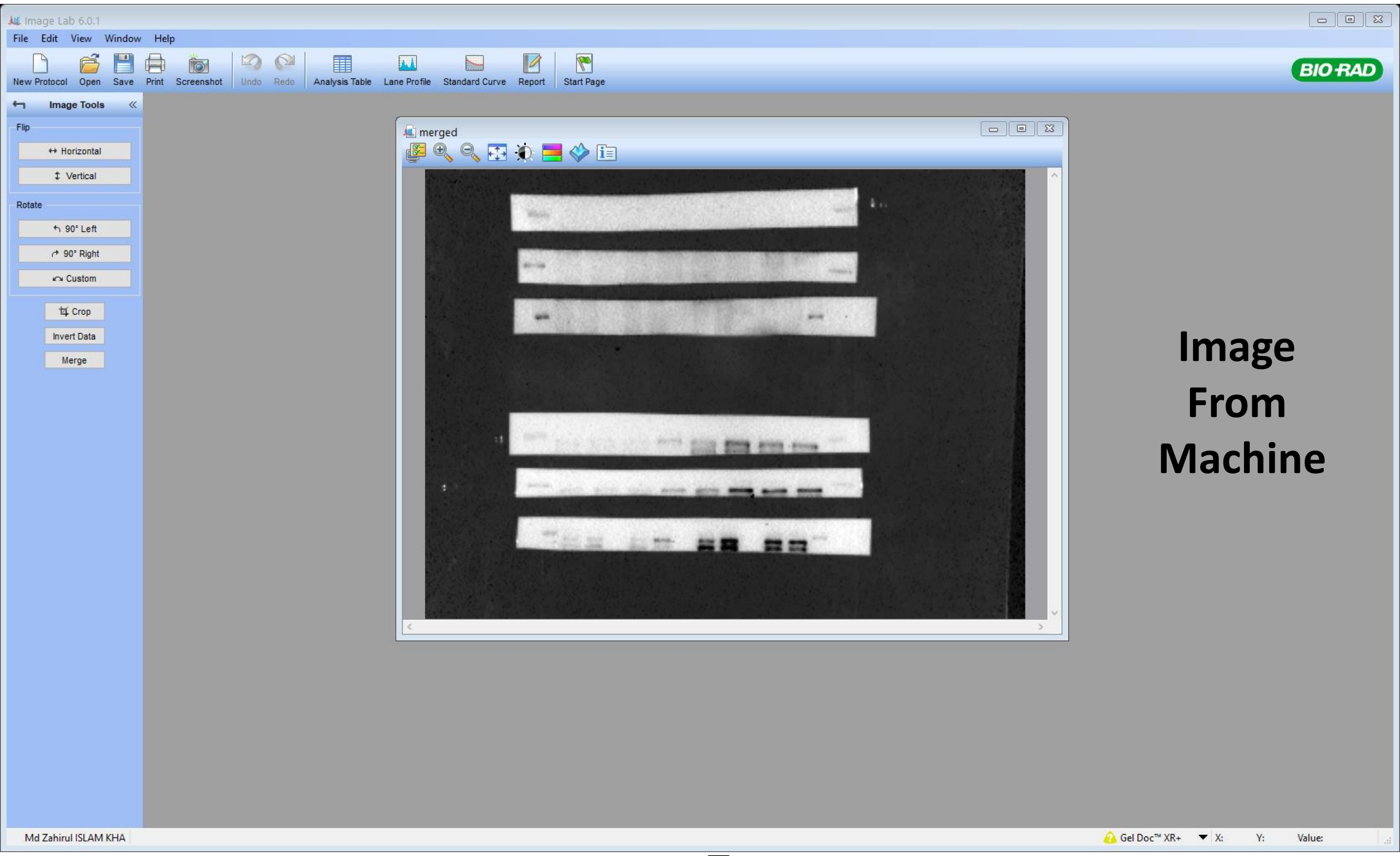

Image From Machine

Cropped Image

N-cadherin-1

N-cadherin-2

N-cadherin-3

E-cadherin-1

E-cadherin-2

E-cadherin-3

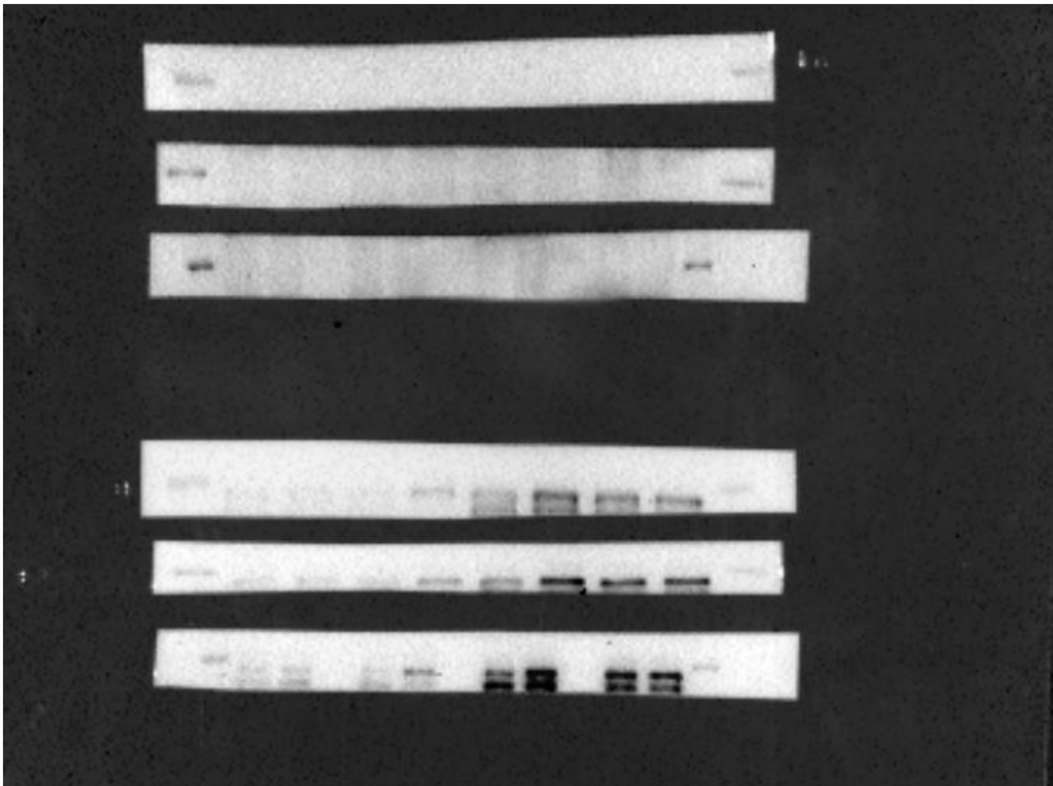

HCT-116
Original Western blot image for Figure 6
Vimentin

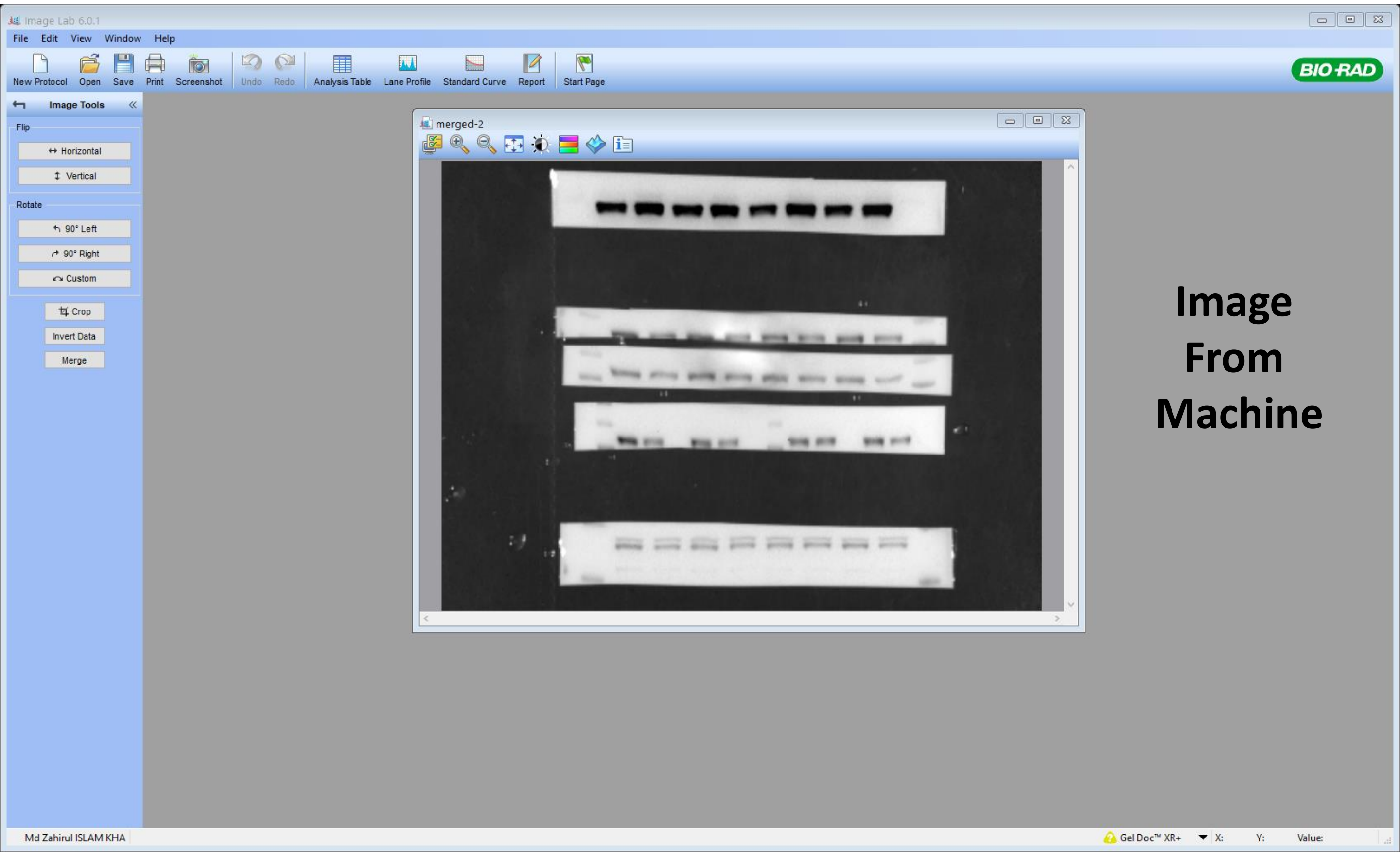

Cropped Image

Vimentin-1

Vimentin-2

Vimentin-3

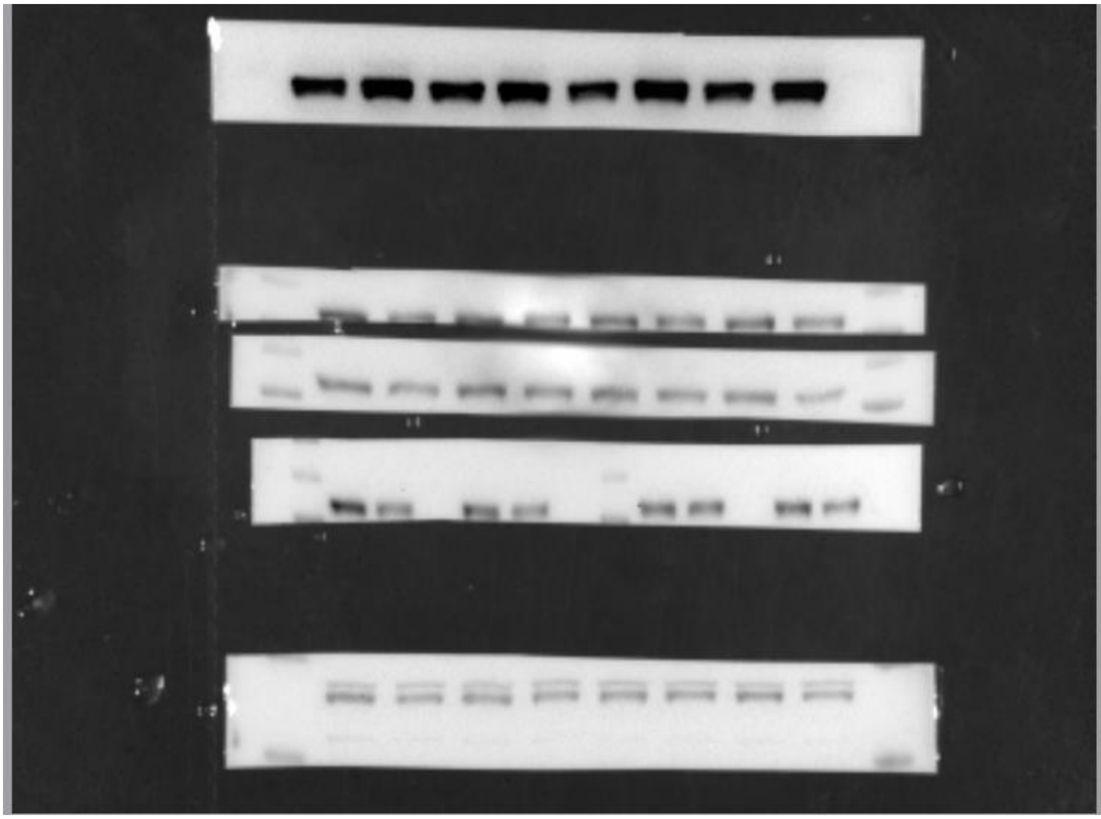

HCT-116
Original Western blot image for Figure 6
N-cadherin

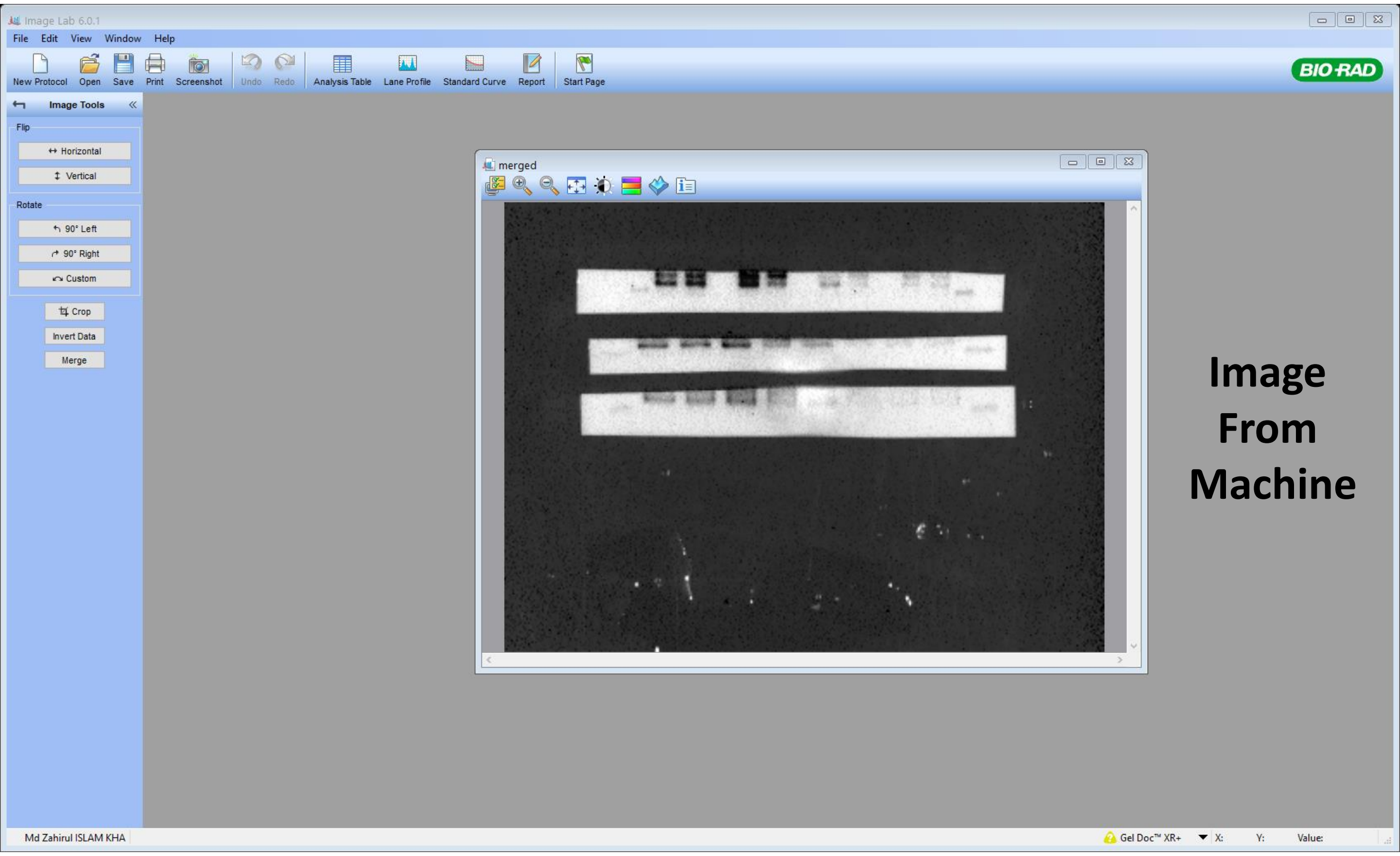

Image From Machine

Cropped Image

N-cadherin-4

N-cadherin-5

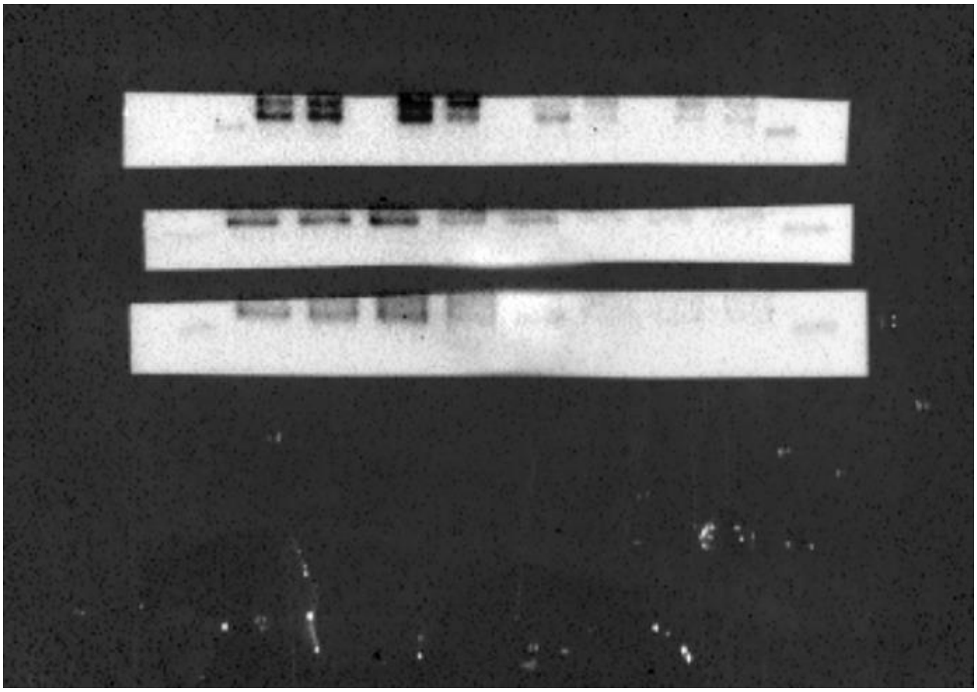

HCT-116
Original Western blot image for Figure 6
N-cadherin

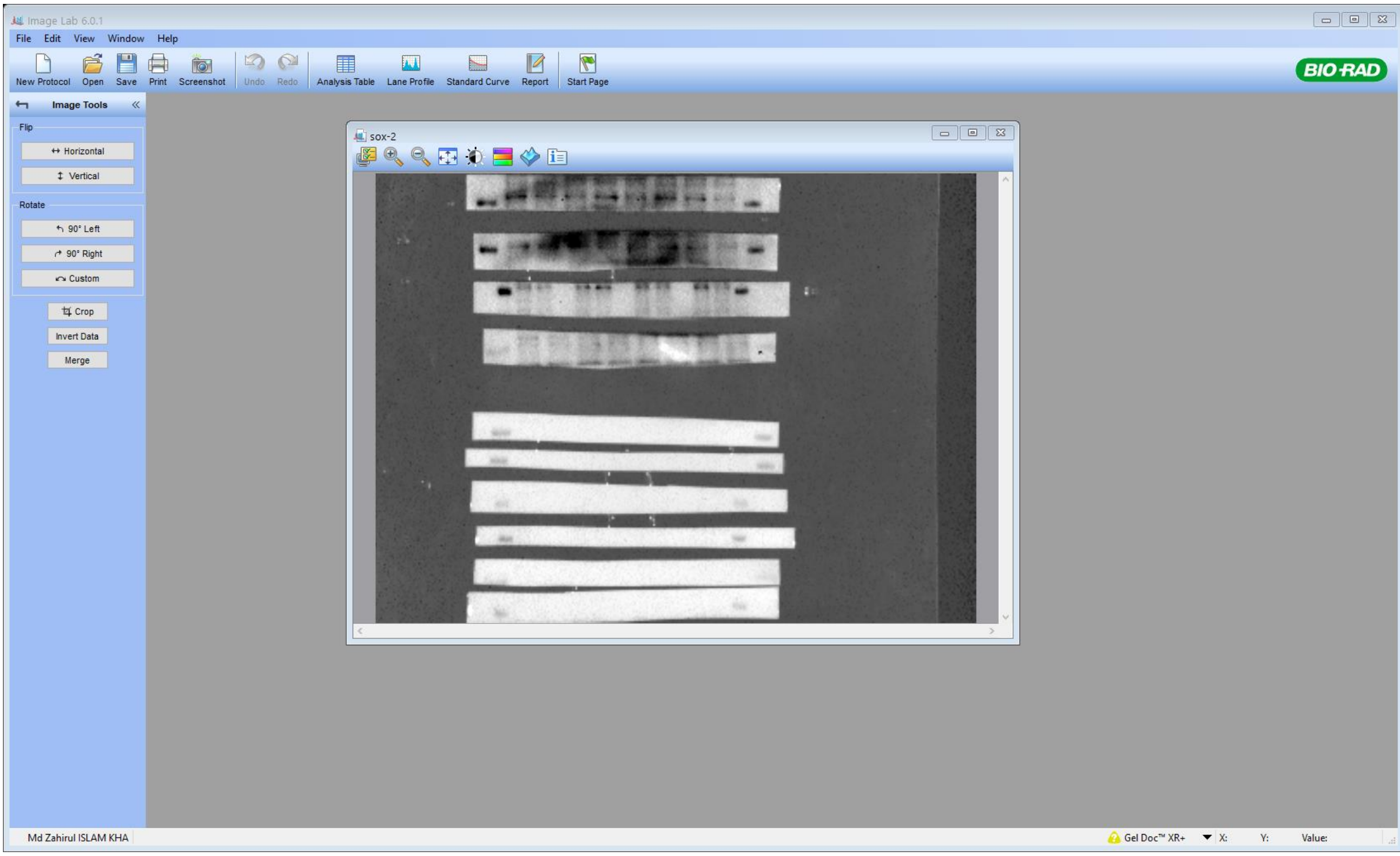

Cropped Image

Sox-2-1

Sox-2-2

Snail-1

Snail-2

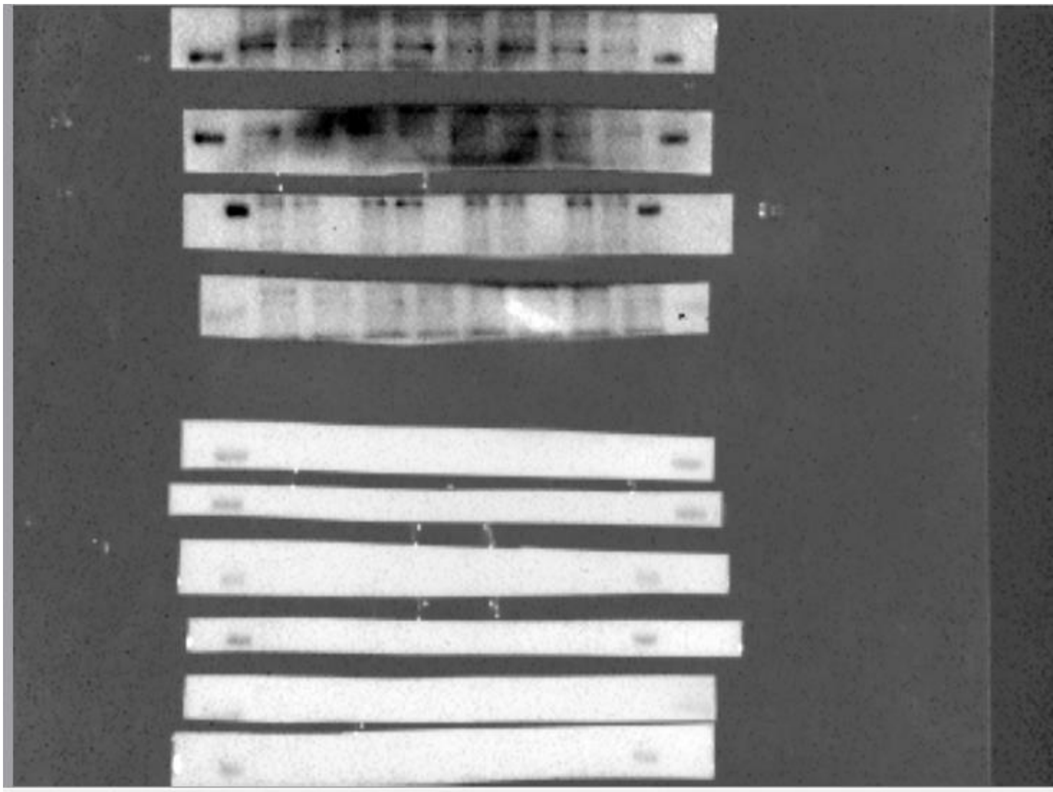

Original Western blot images

SW480

SW480

Original Western blot image for Figure 3

GAPDH

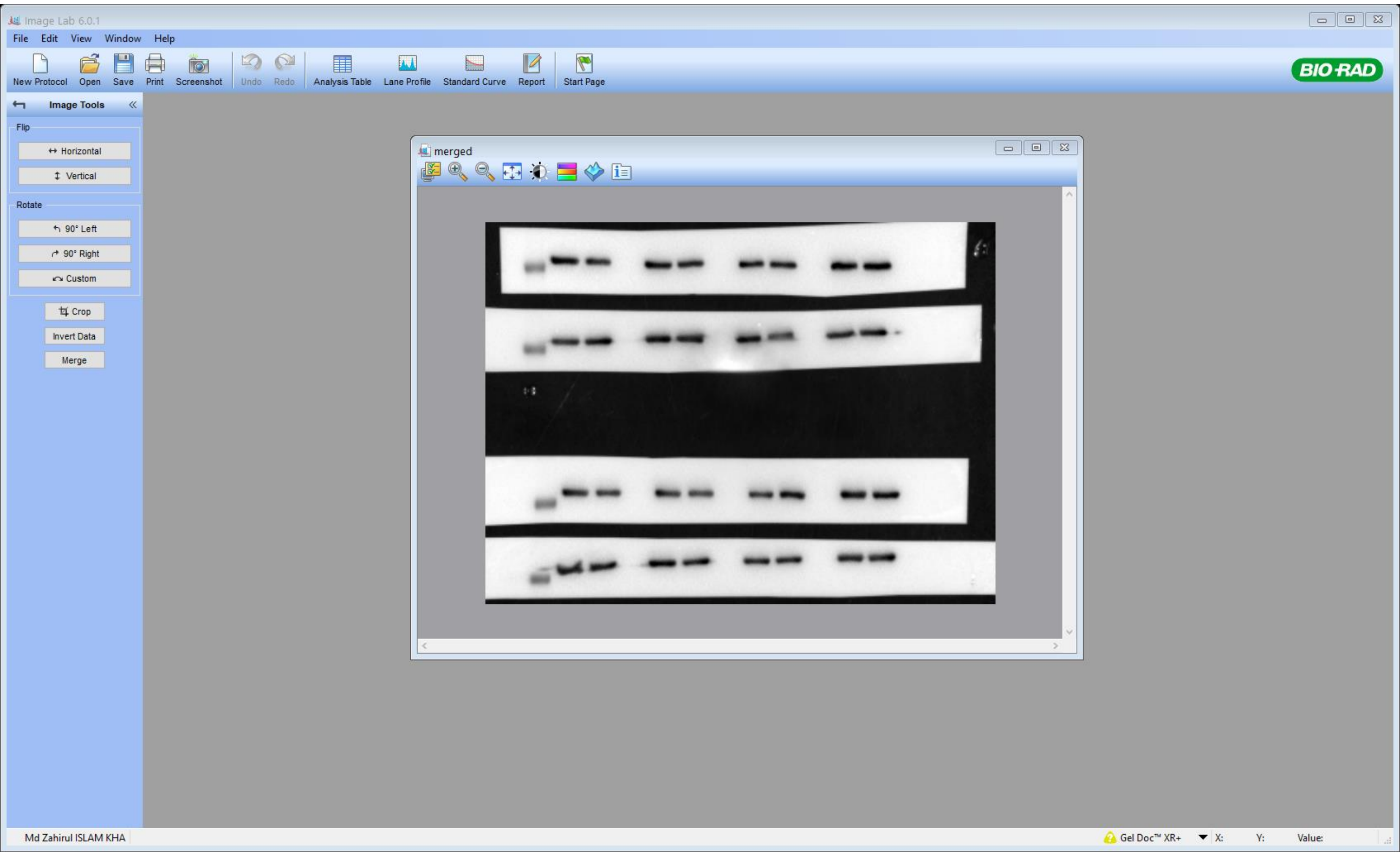

Cropped Image

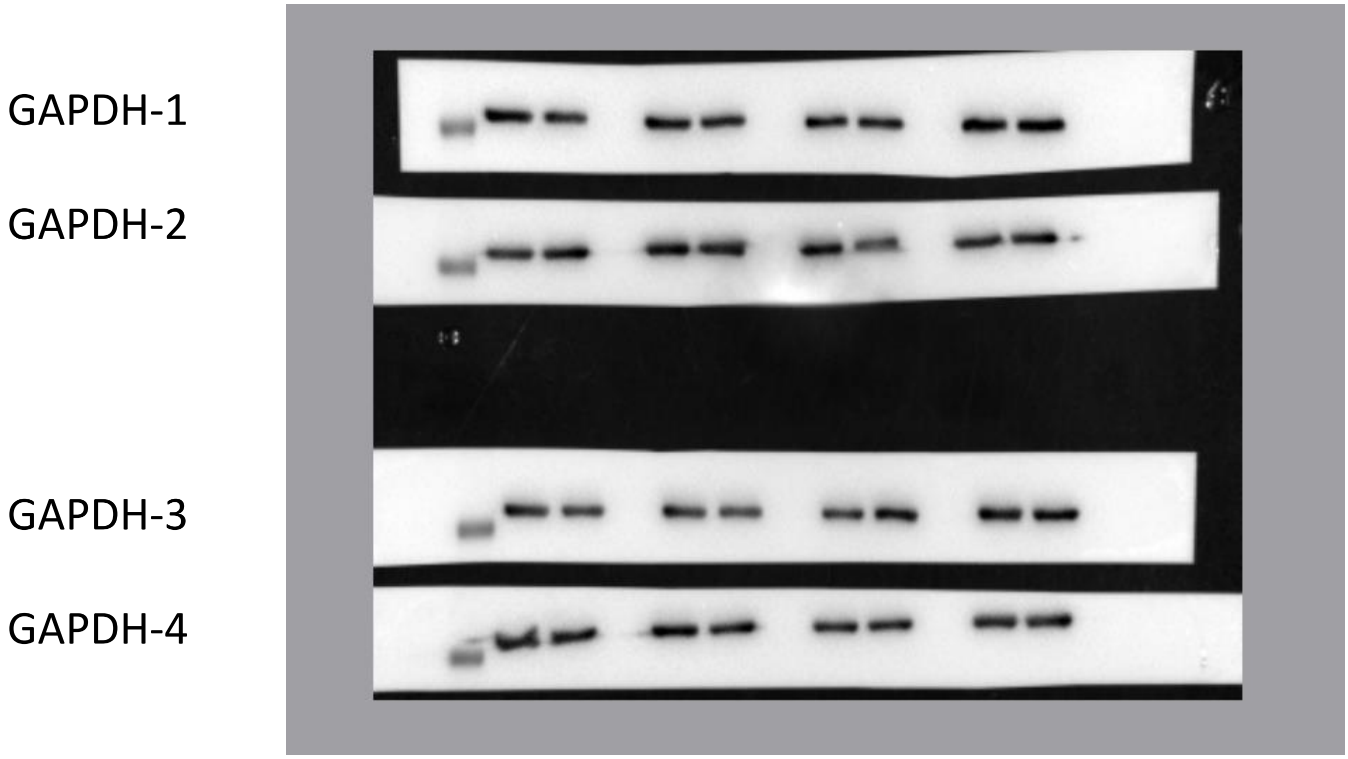

SW480

Original Western blot image for Figure 3

LC3B

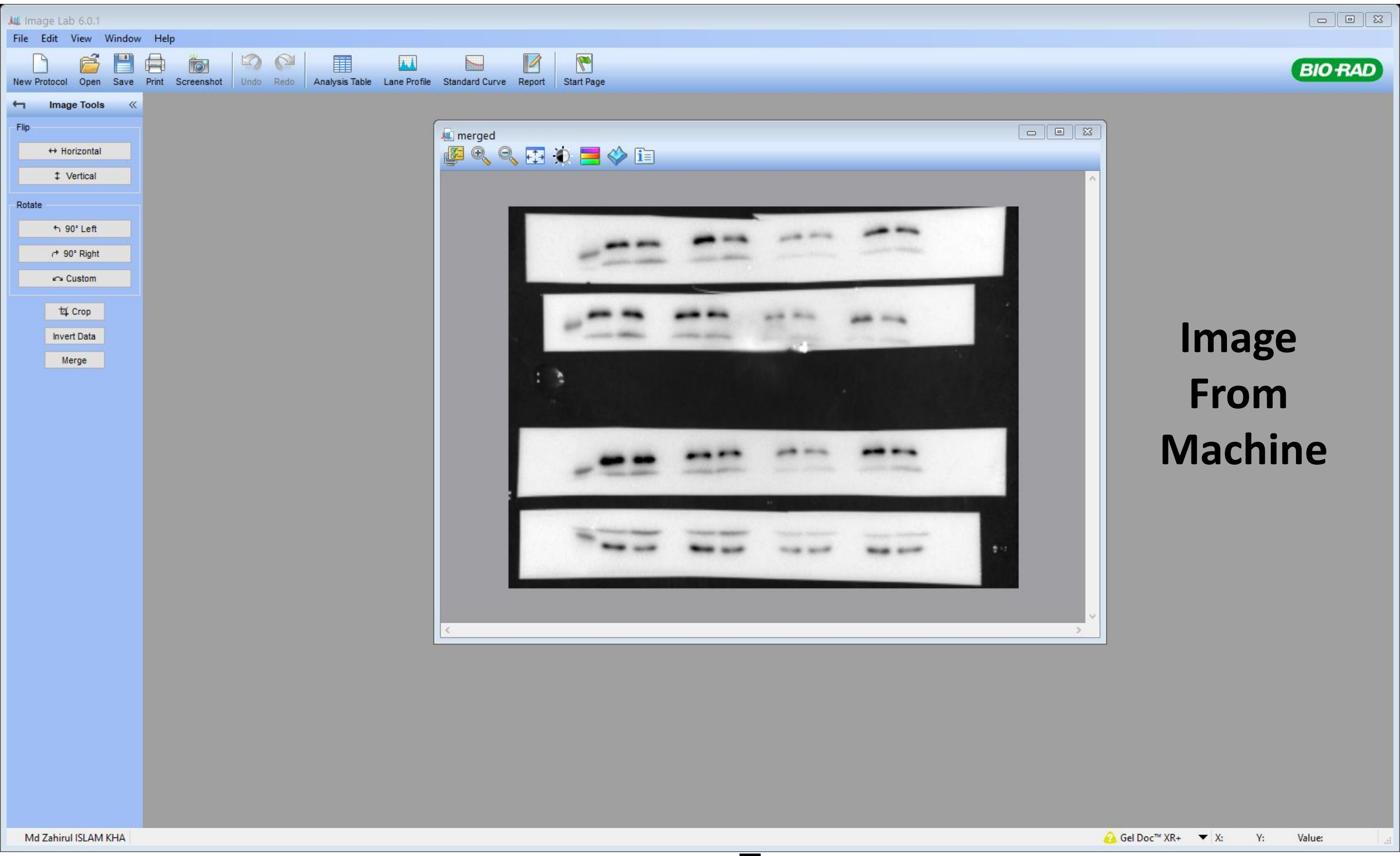

Image From Machine

Cropped Image

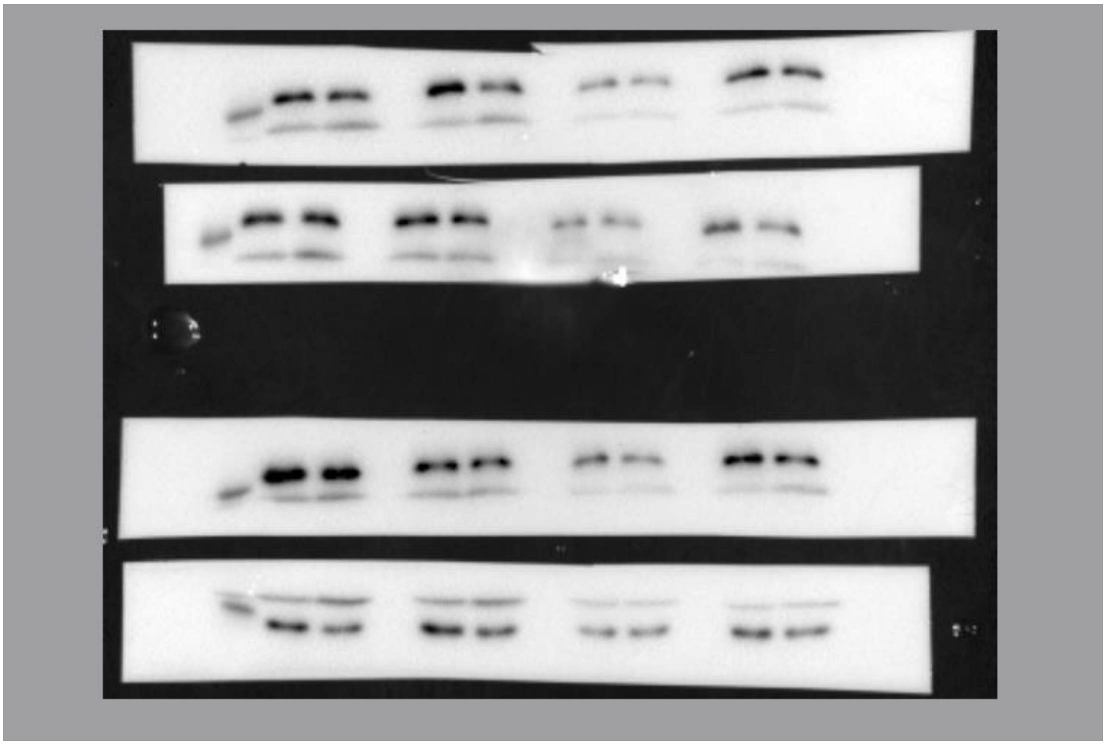

LC3B-1

LC3B-2

LC3B-3

LC3B-4

SW480

Original Western blot image for Figure 3

p-62

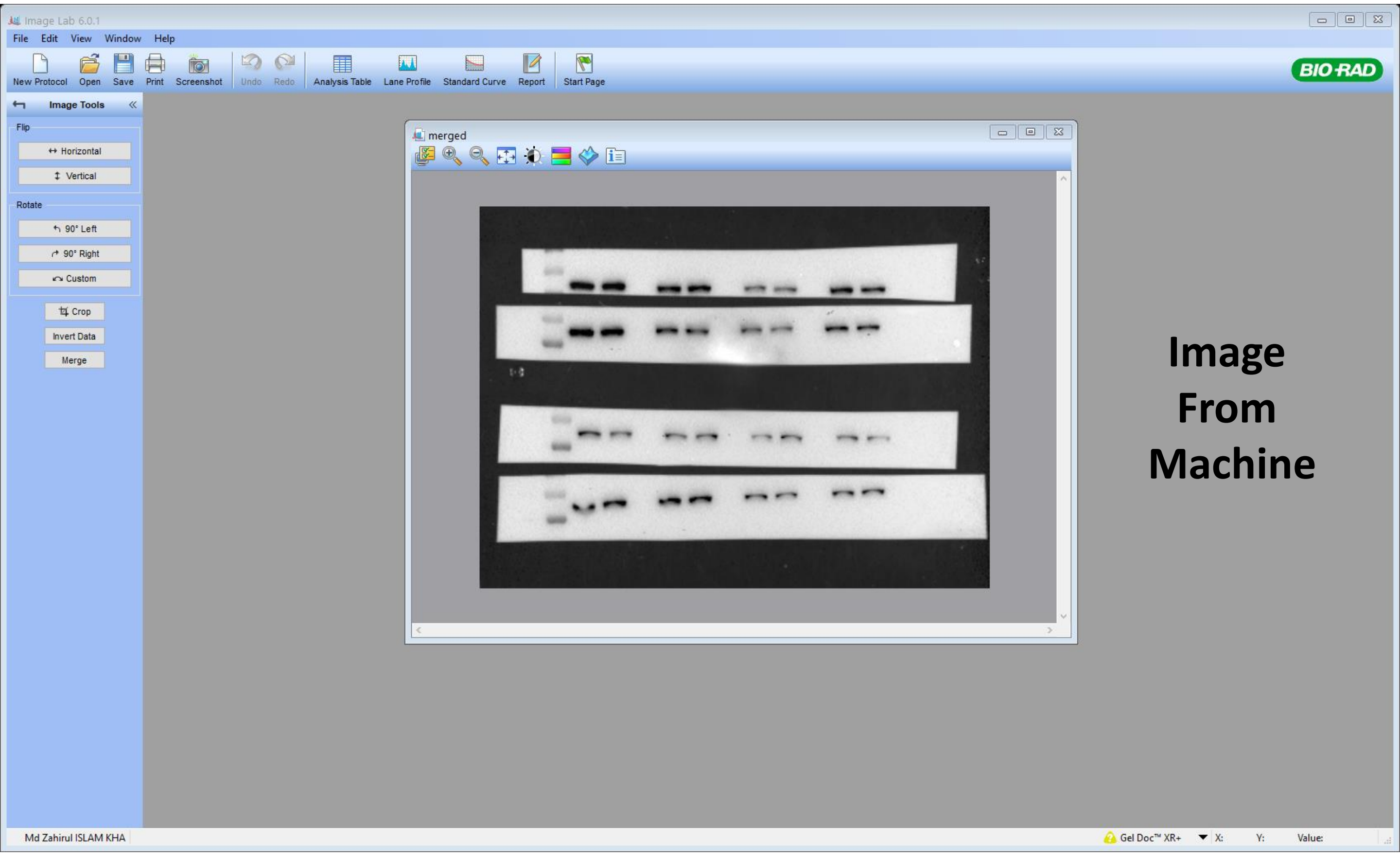

Image From Machine

Cropped Image

p-62-1

p-62-2

p-62-3

p-62-4

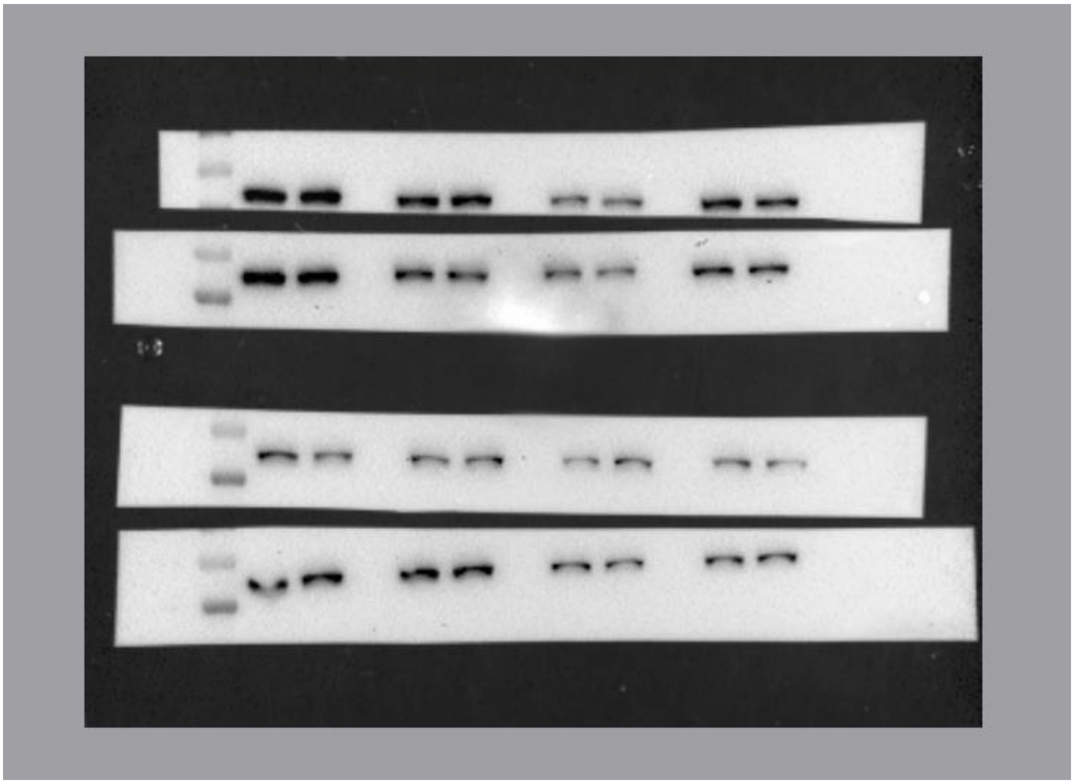

SW480

Original Western blot image for Figure 3

Beclin-1

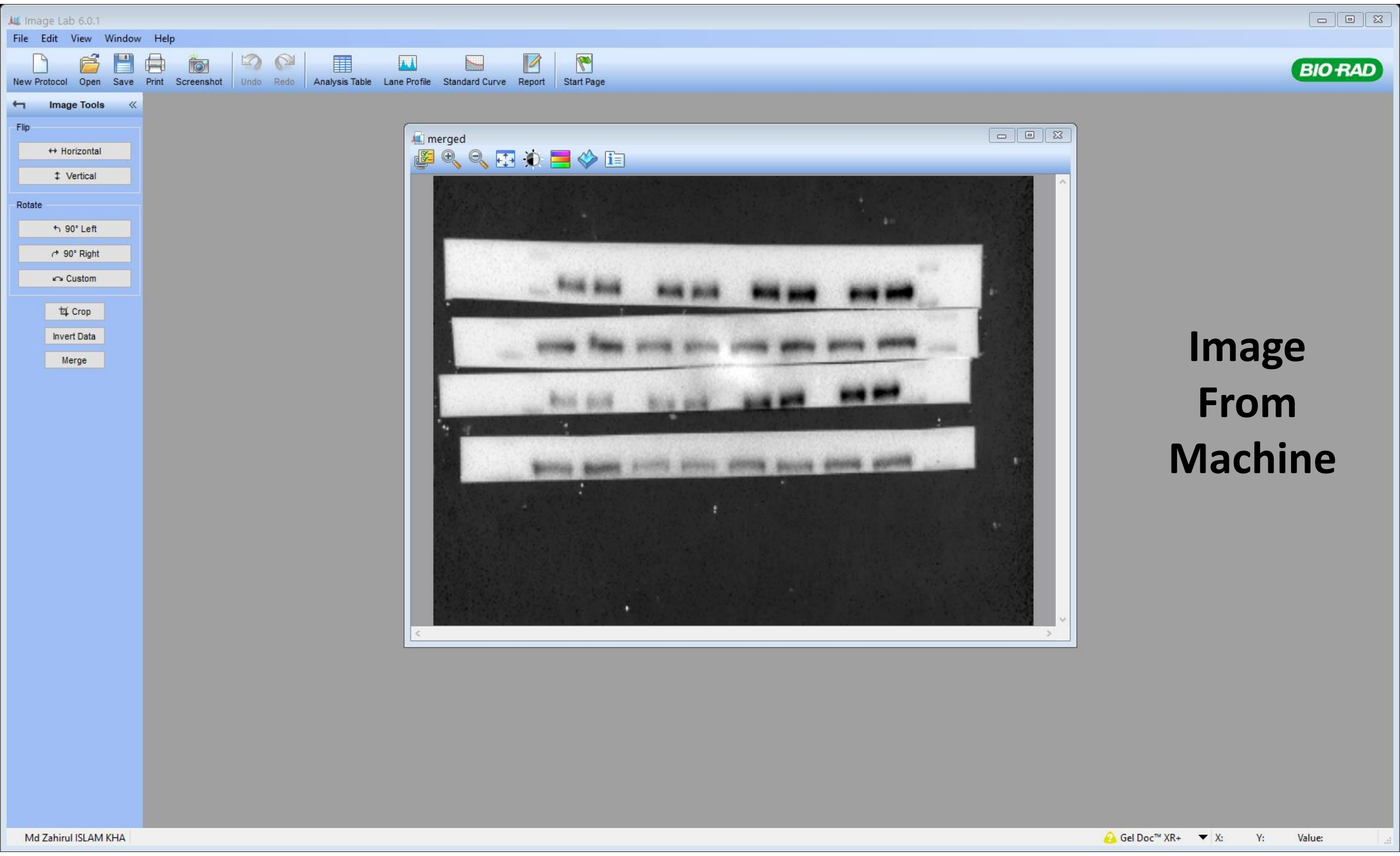

Image From Machine

Cropped Image

Beclin-1-1

Beclin-1-2

Beclin-1-3

Beclin-1-4

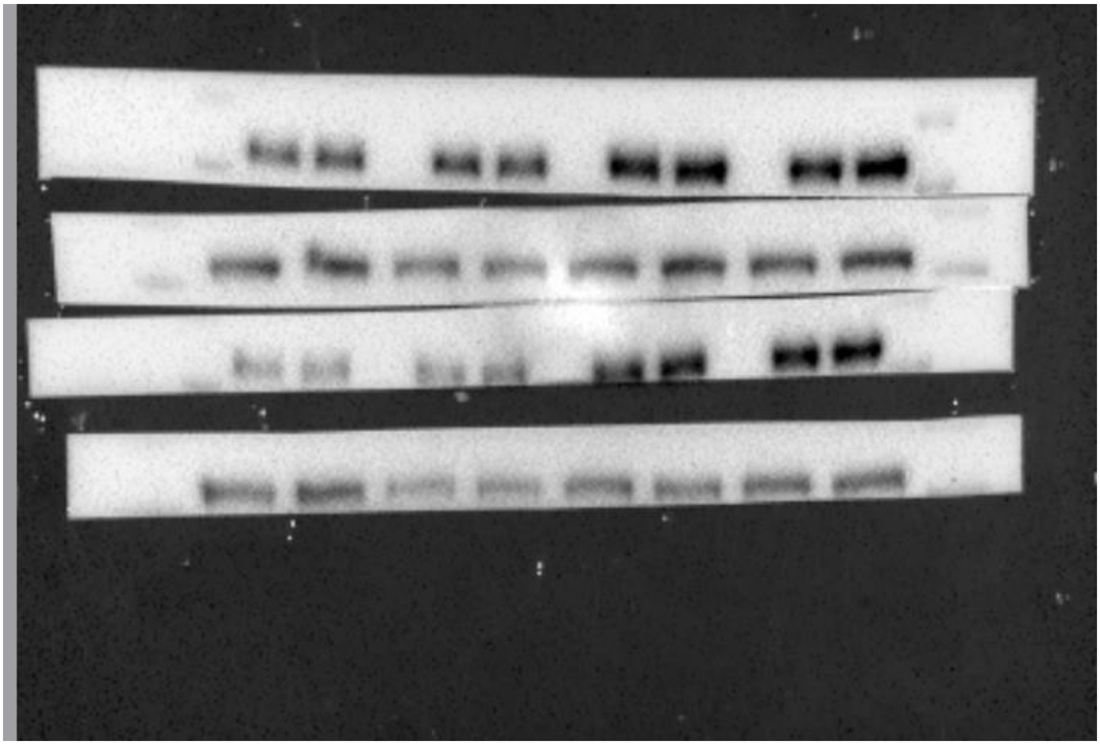

SW480

Original Western blot image for Figure 4

β-actin

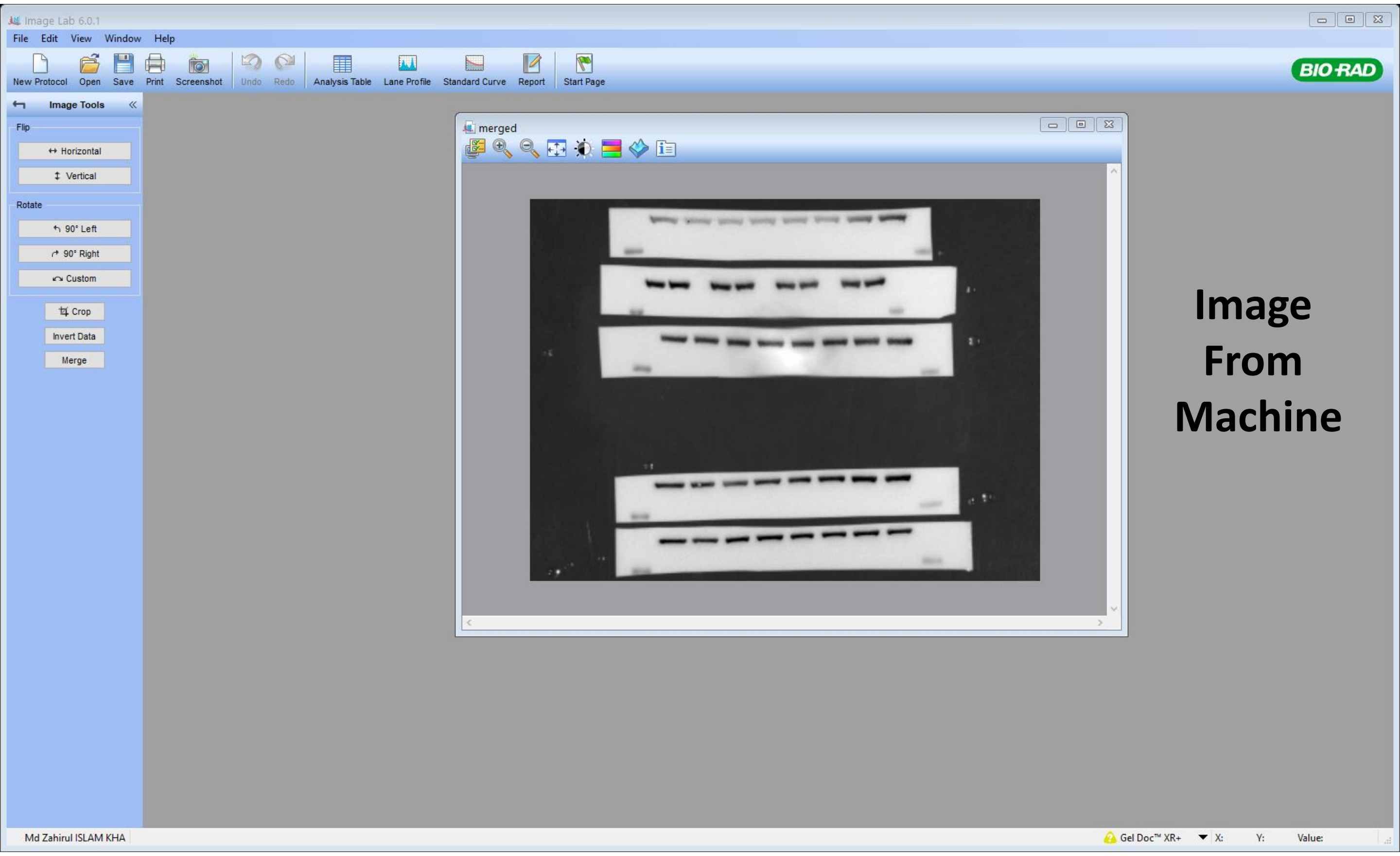

Image From Machine

Cropped Image

β-actin-1

β-actin-2

β-actin-3

β-actin-4

β-actin-5

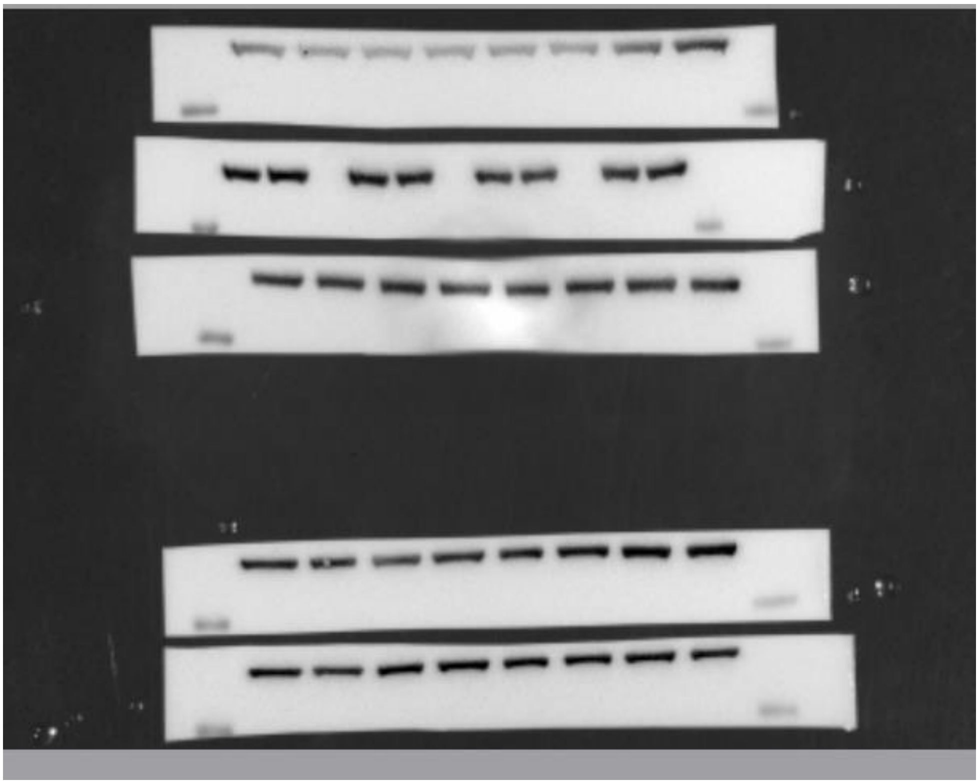

SW480

Original Western blot image for Figure 4

Bcl-2

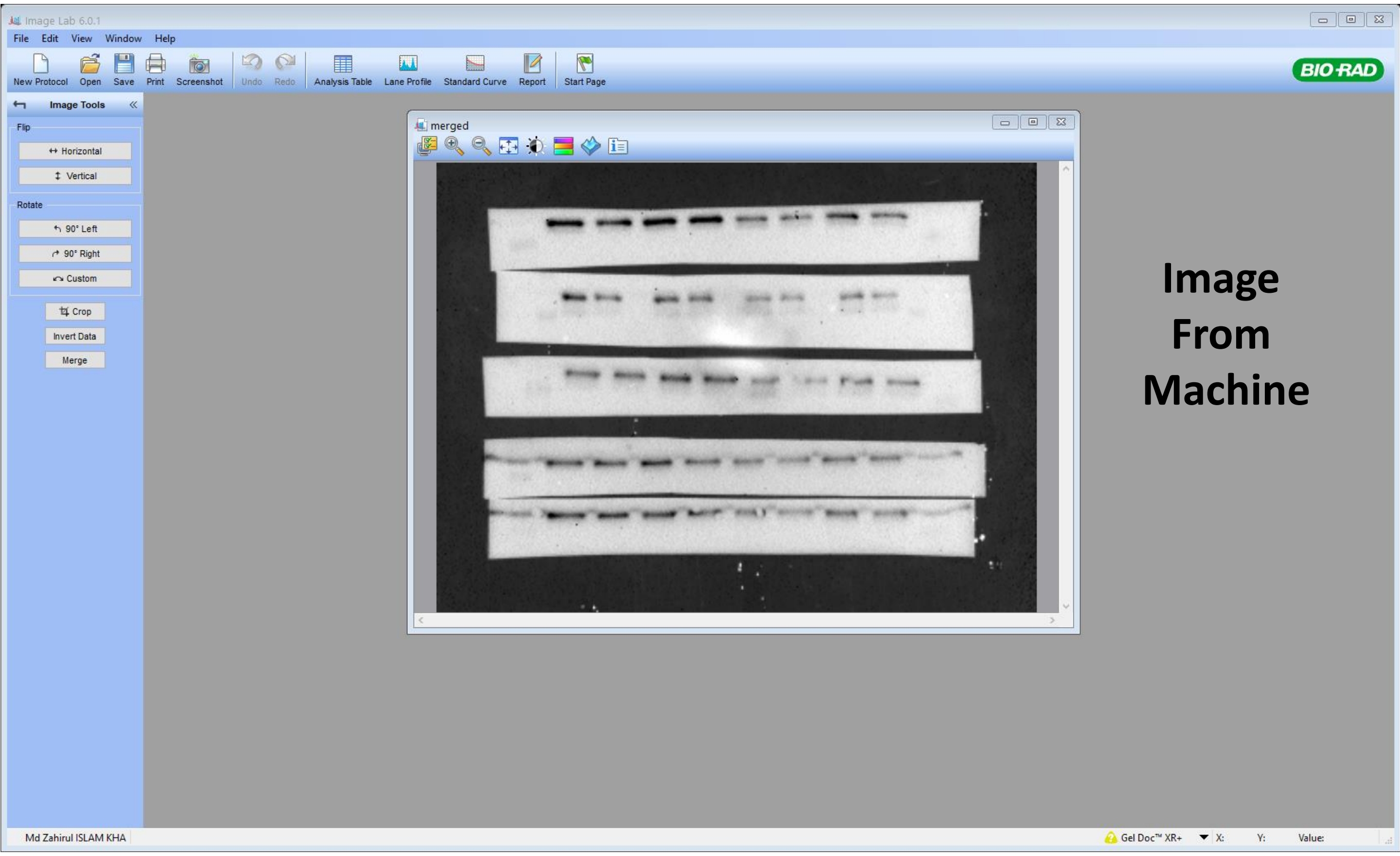

Image From Machine

Cropped Image

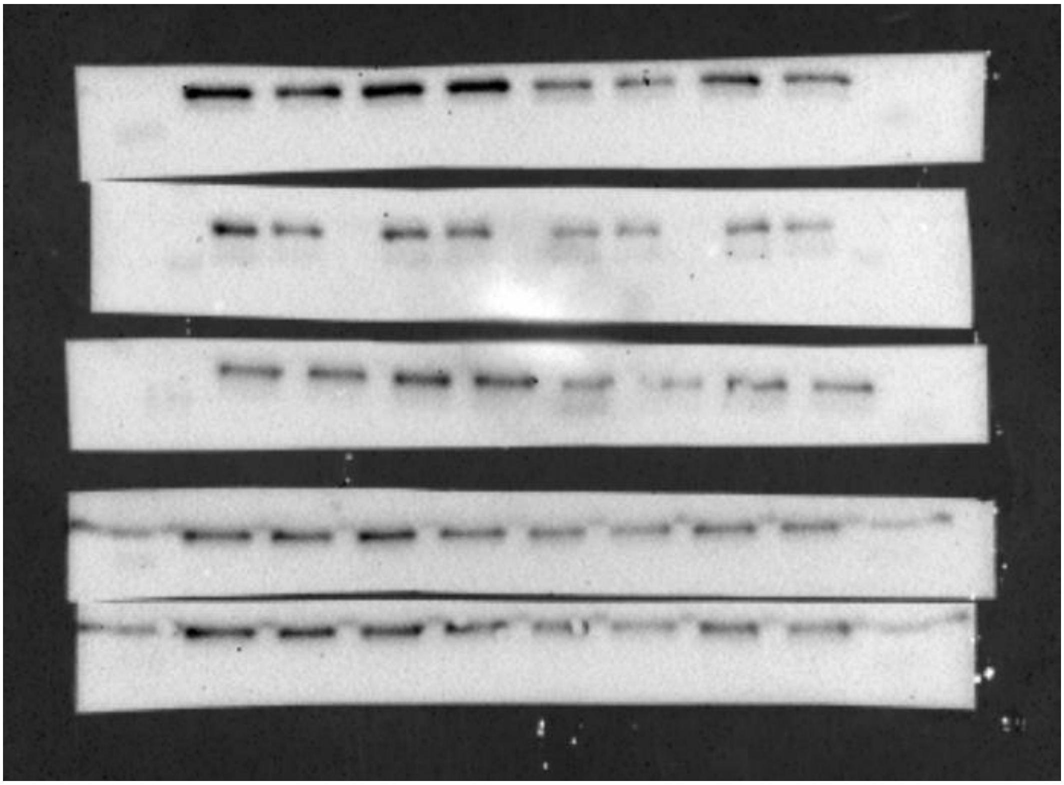

Bcl-2-1

Bcl-2-2

Bcl-2-3

Bcl-2-4

Bcl-2-5

SW480

Original Western blot image for Figure 4

Bcl-xL

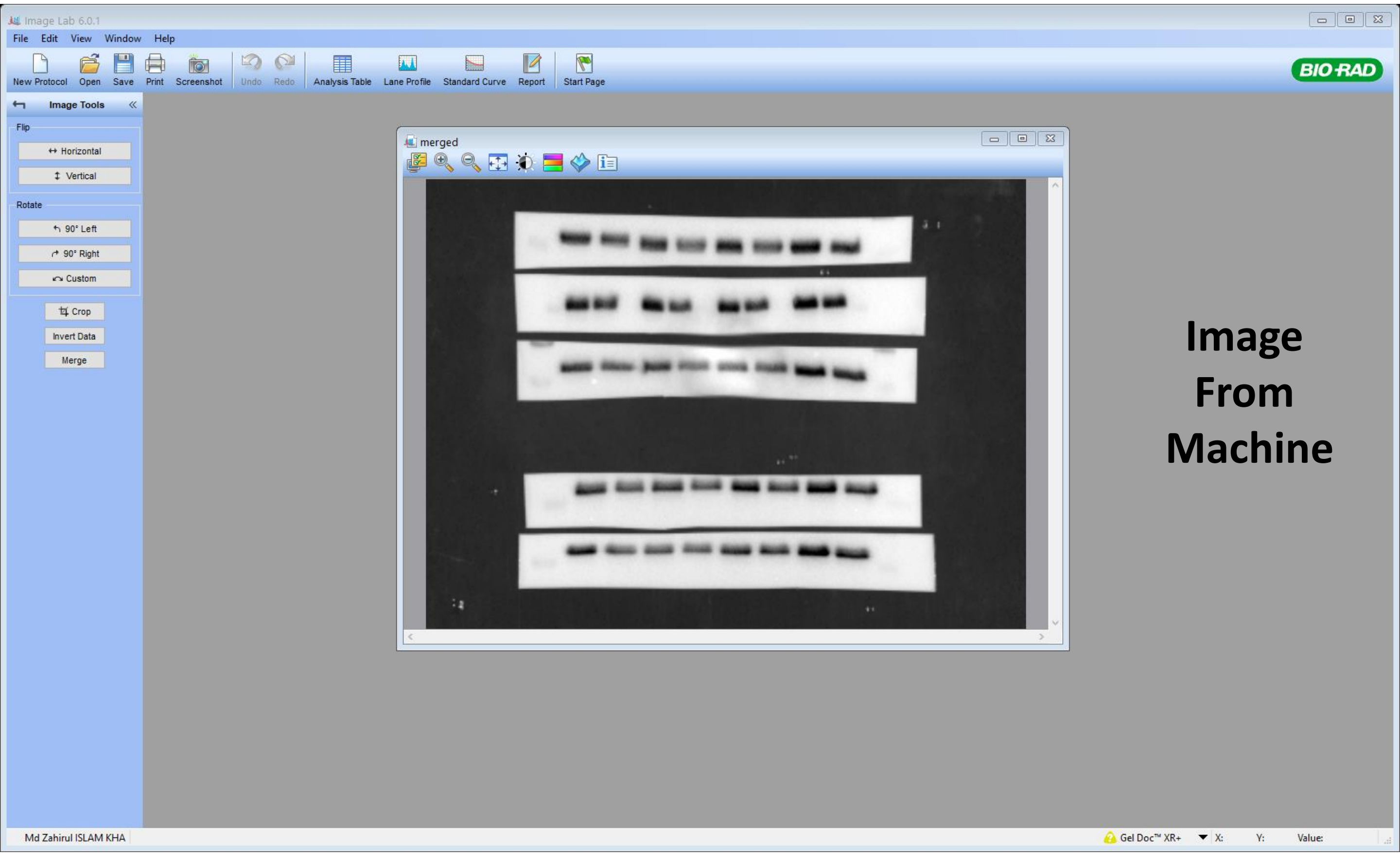

Cropped Image

Bcl-xL-1

Bcl-xL-2

Bcl-xL-3

Bcl-xL-4

Bcl-xL-5

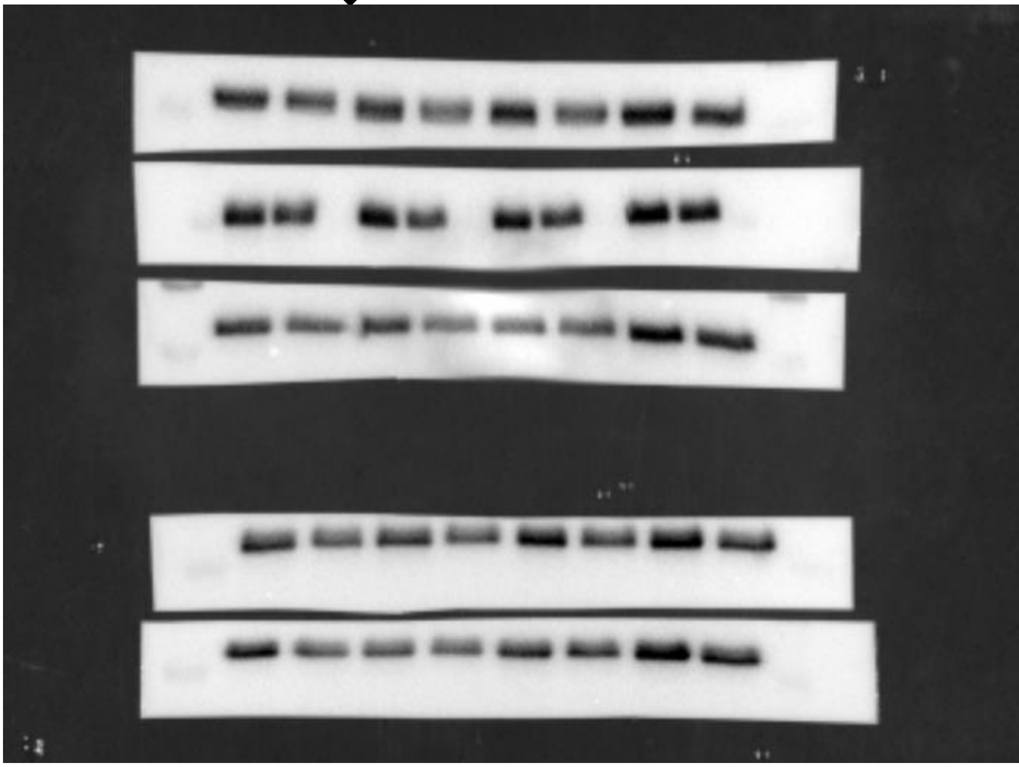

SW480

Original Western blot image for Figure 4

Procaspase-9

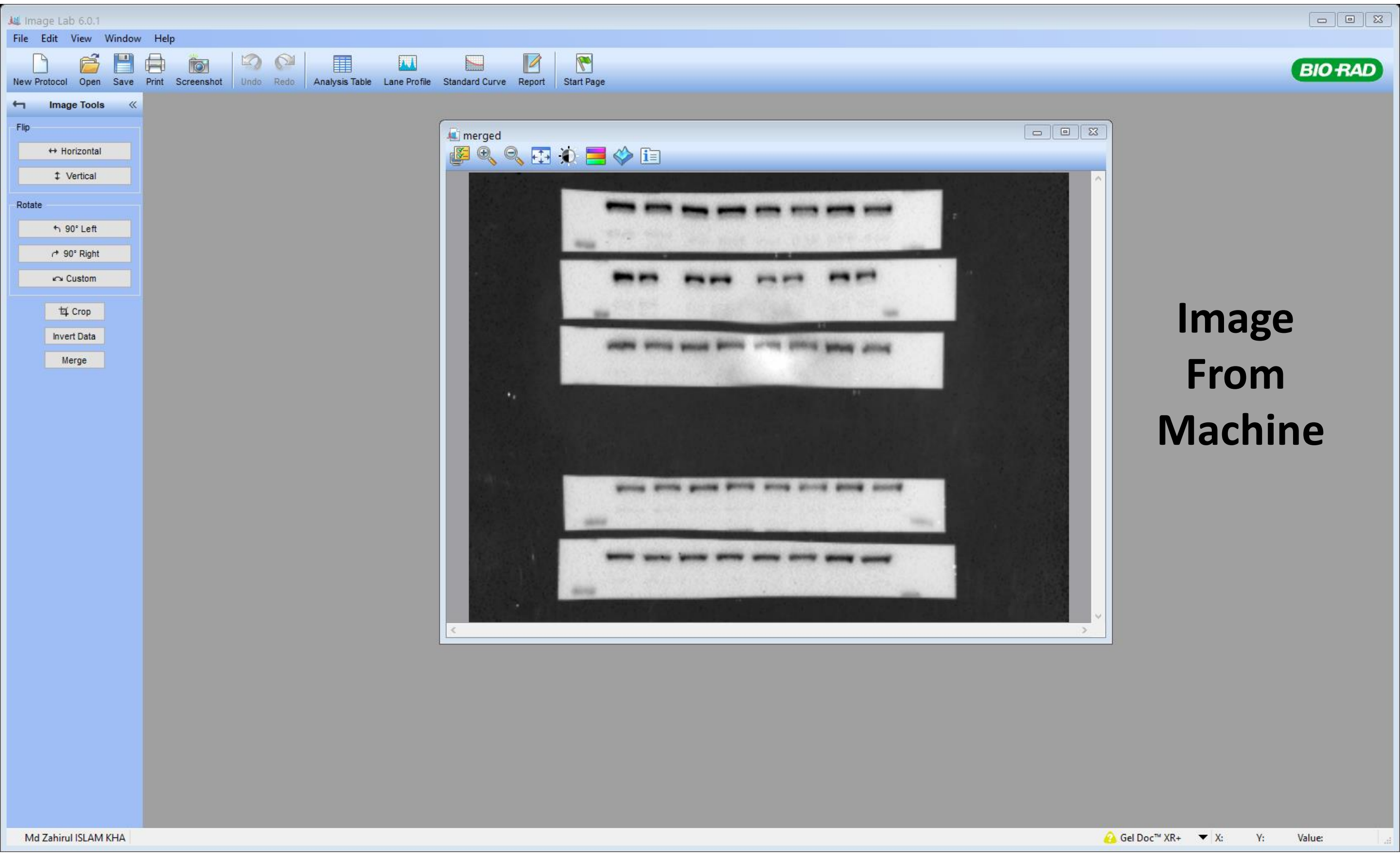

Image From Machine

Cropped Image

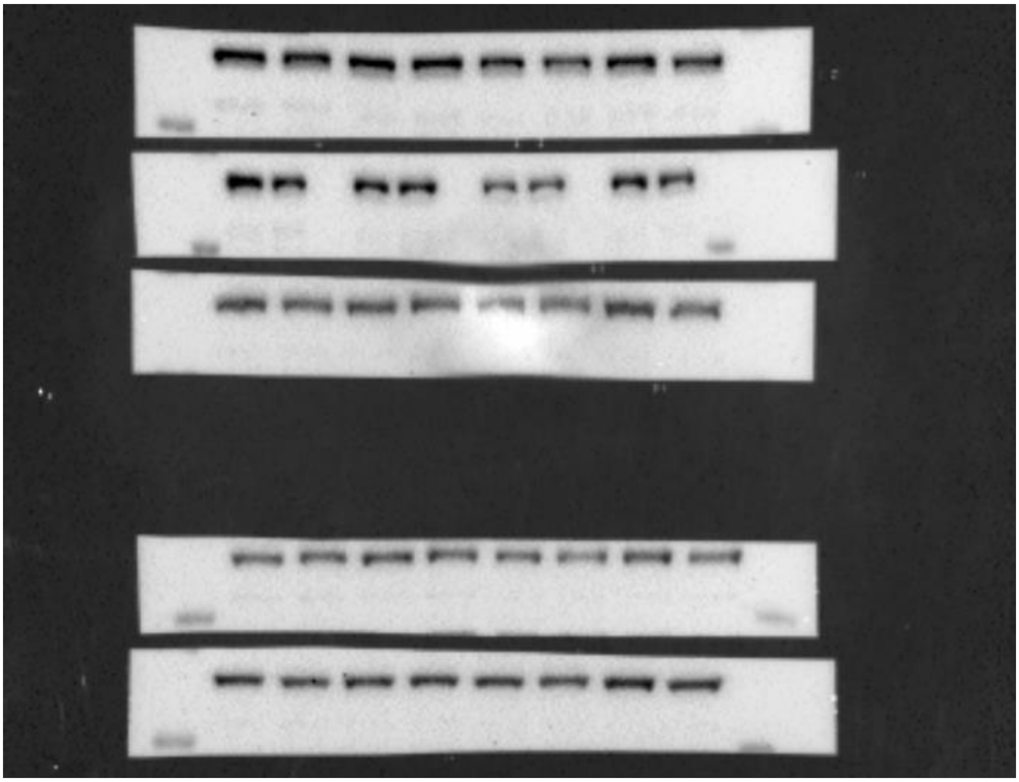

Procaspase-9-1

Procaspase-9-2

Procaspase-9-3

Procaspase-9-4

Procaspase-9-5

SW480

Original Western blot image for Figure 5-6

β-actin

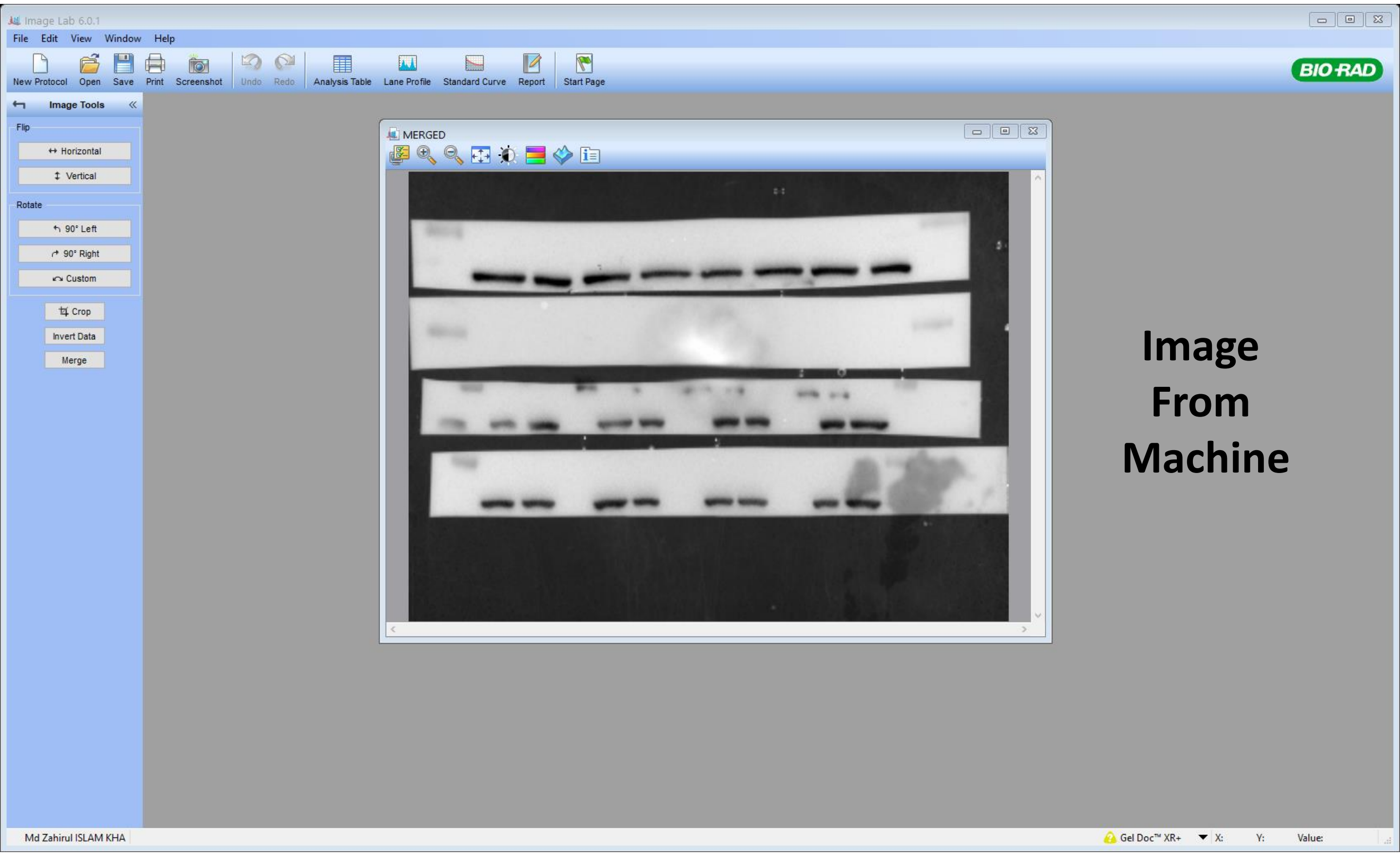

Image From Machine

Cropped Image

β-actin-1

β-actin-2

β-actin-3

β-actin-4

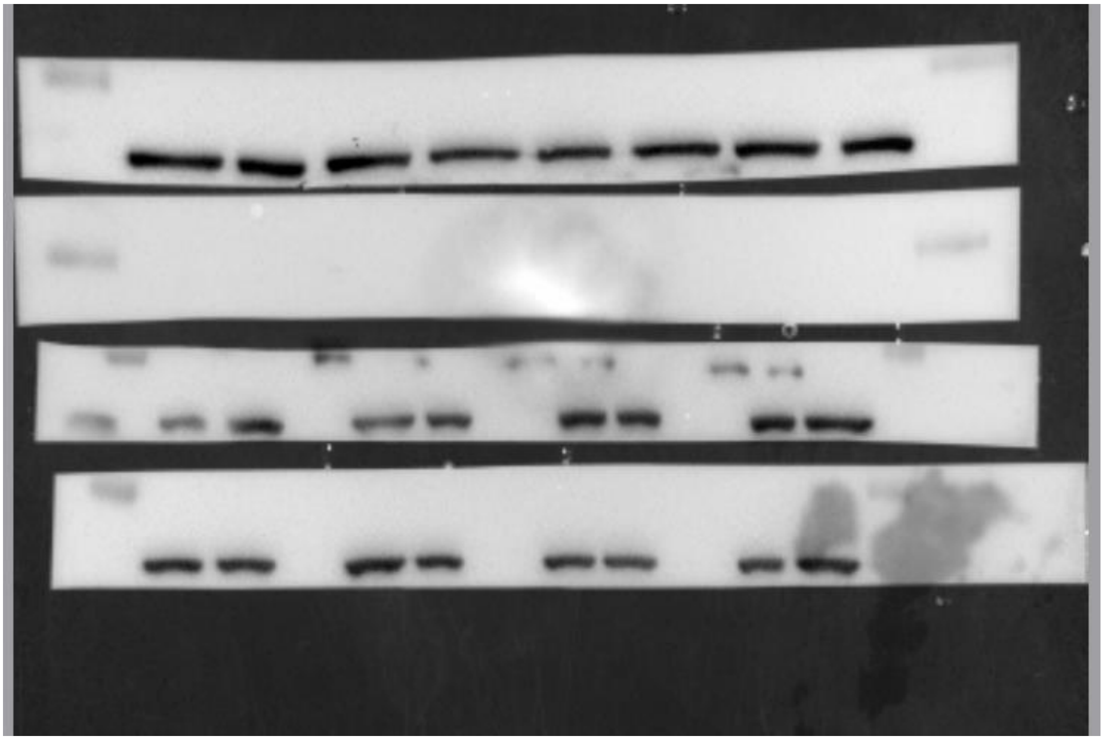

SW480

Original Western blot image for Figure 5-6

β-actin

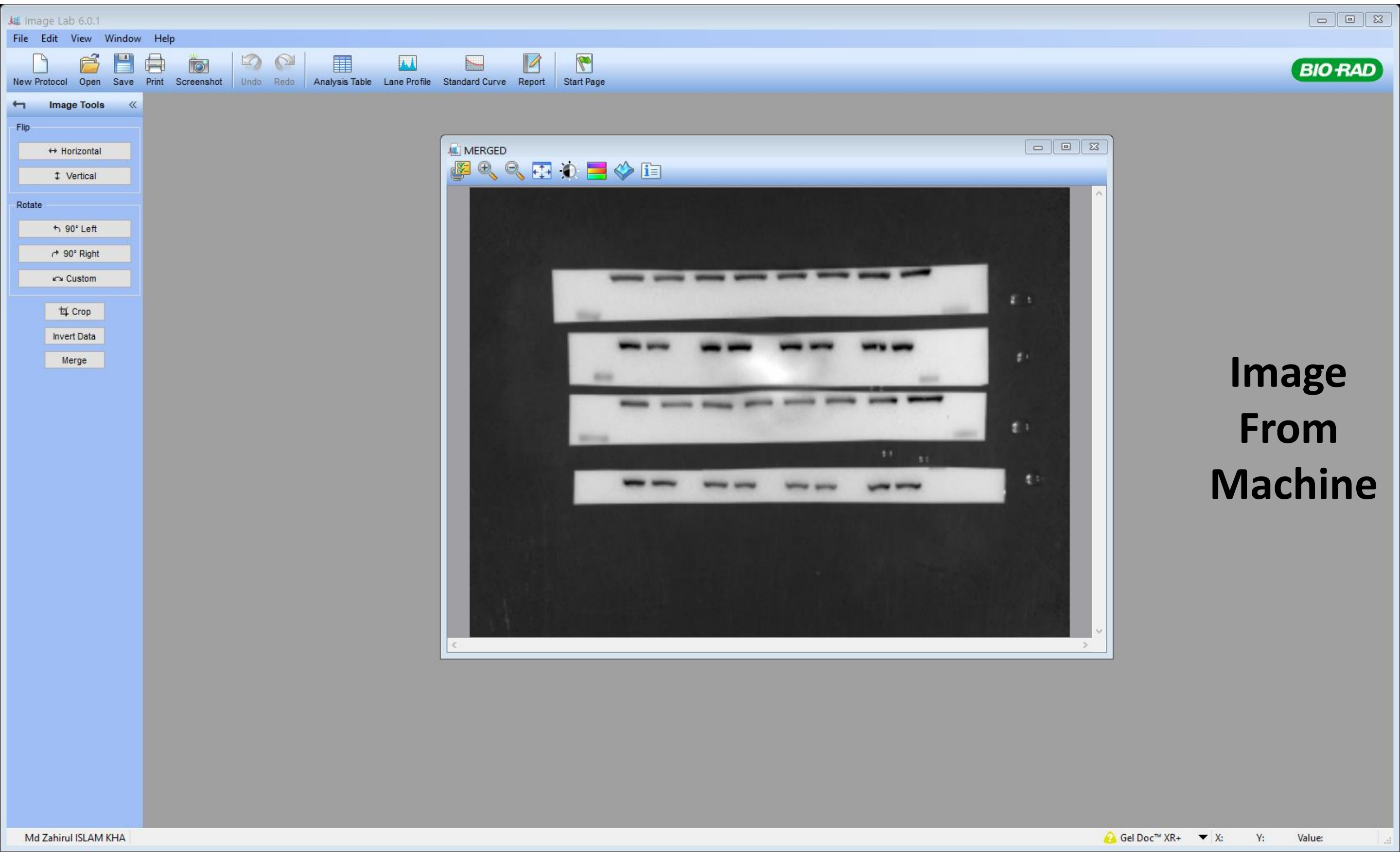

Image From Machine

Cropped Image

β-actin-5

β-actin-6

β-actin-7

β-actin-8

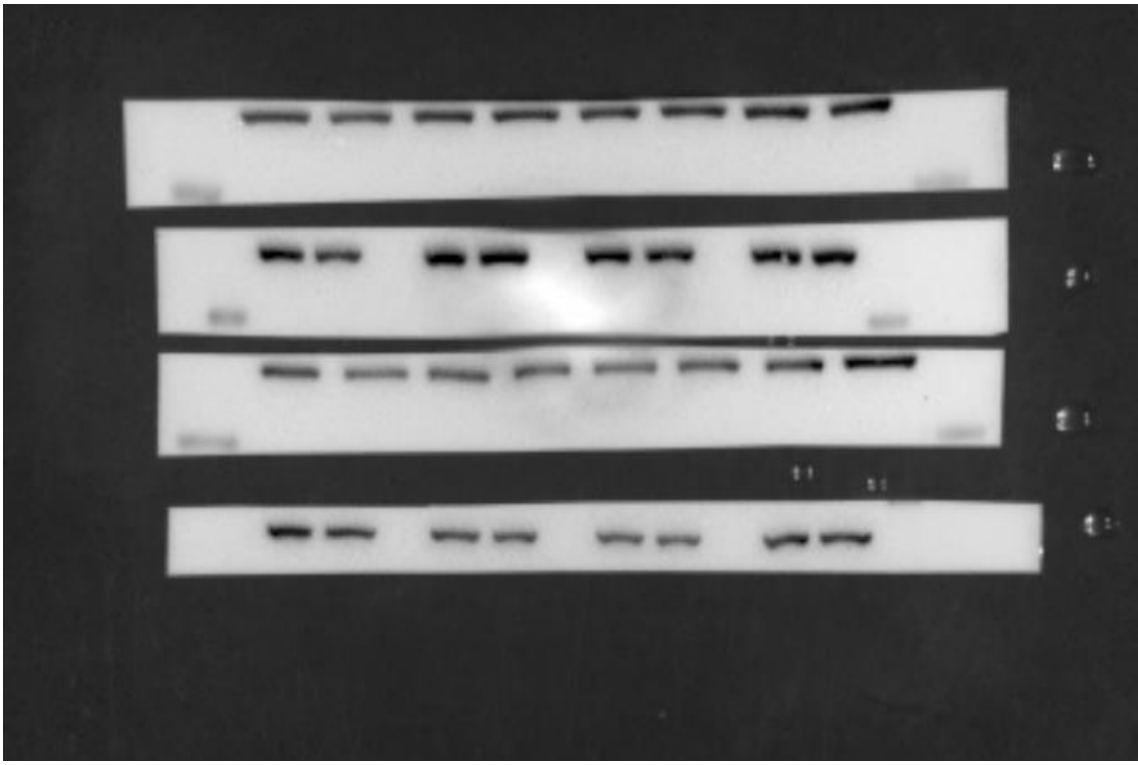

SW480

Original Western blot image for Figure 5-6

β-actin

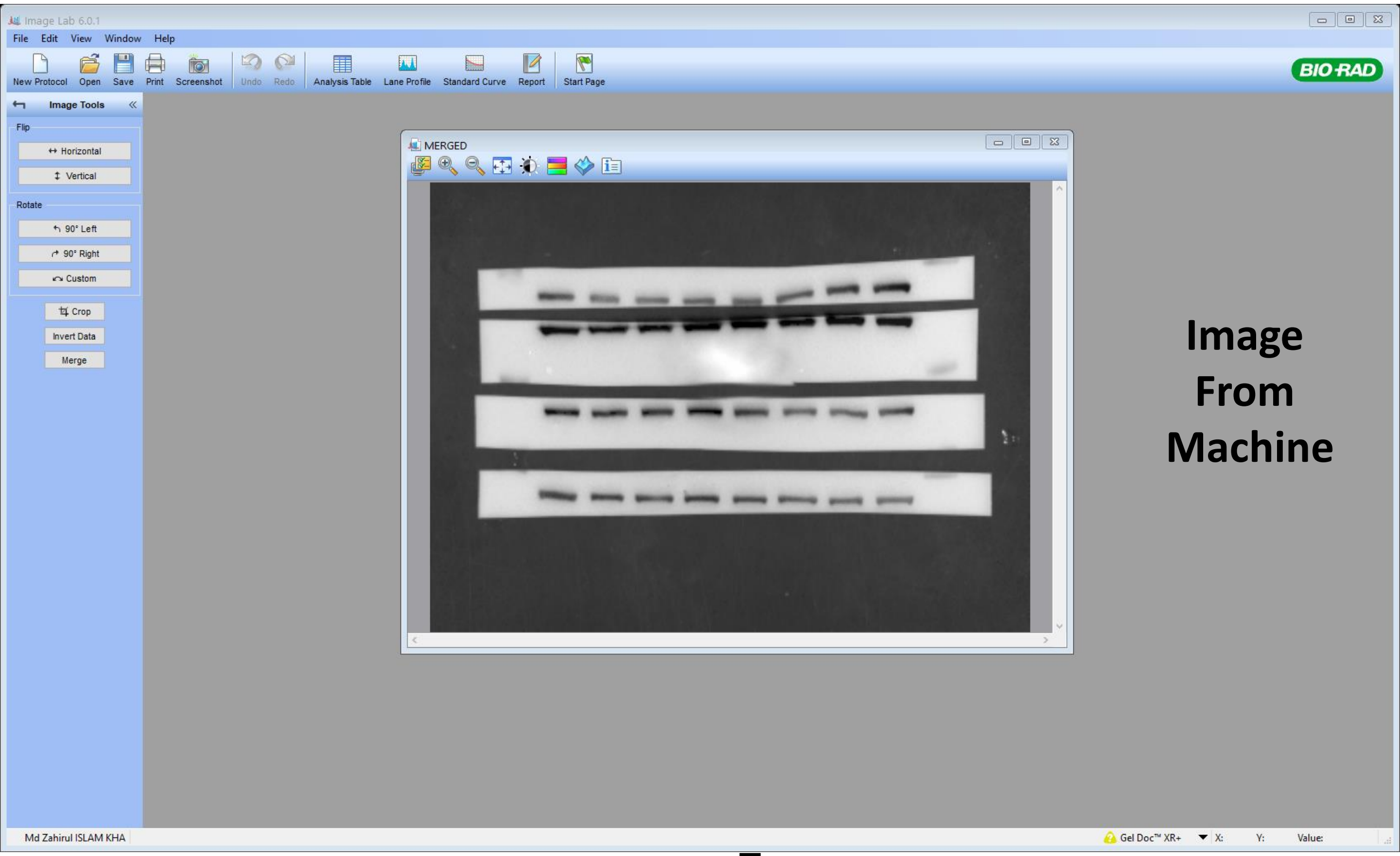

Cropped Image

β-actin-9

β-actin-10

β-actin-11

β-actin-12

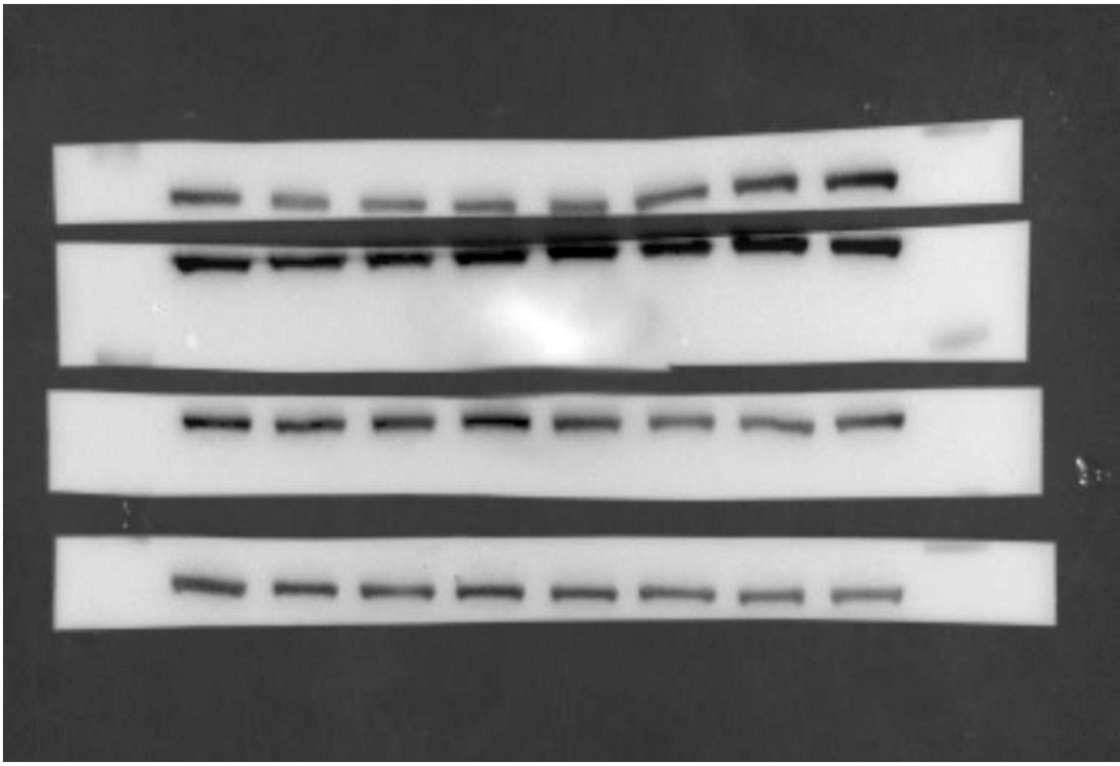

SW480

Original Western blot image for Figure 5-6

β-actin

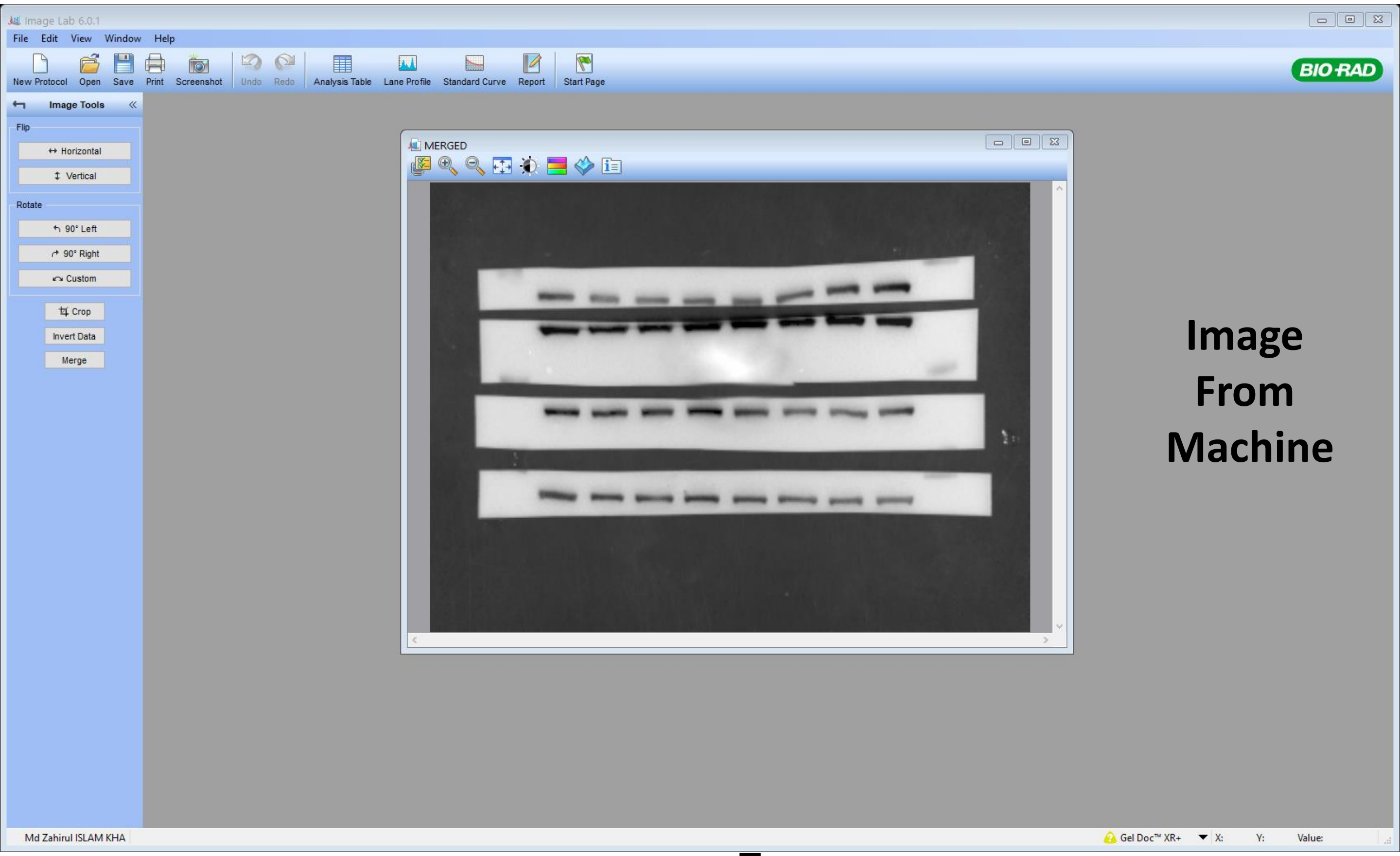

Image From Machine

Cropped Image

β-actin-9

β-actin-10

β-actin-11

β-actin-12

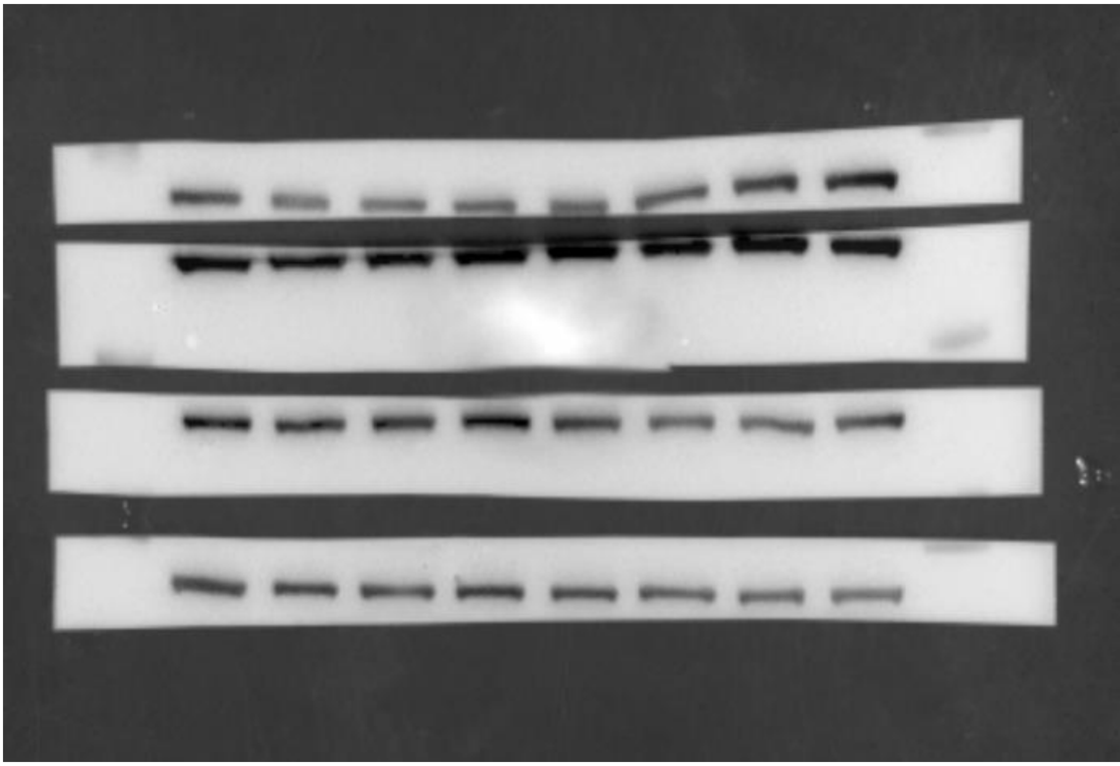

SW480

Original Western blot image for Figure 5-6

AKT, AMPK, Vimentin

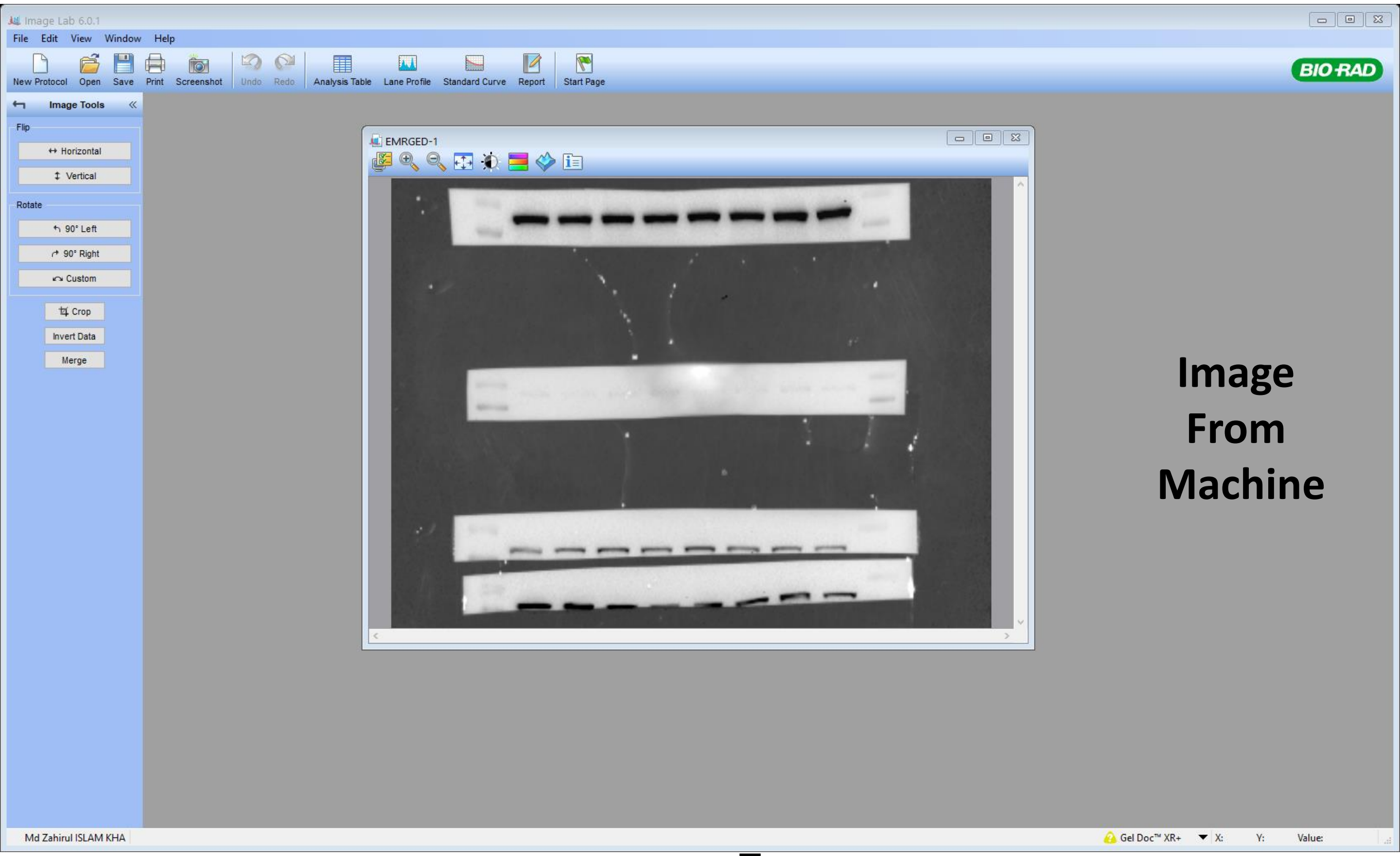

Image From Machine

Cropped Image

AMPK-1

AKT-1

AKT-2

Vimentin-1

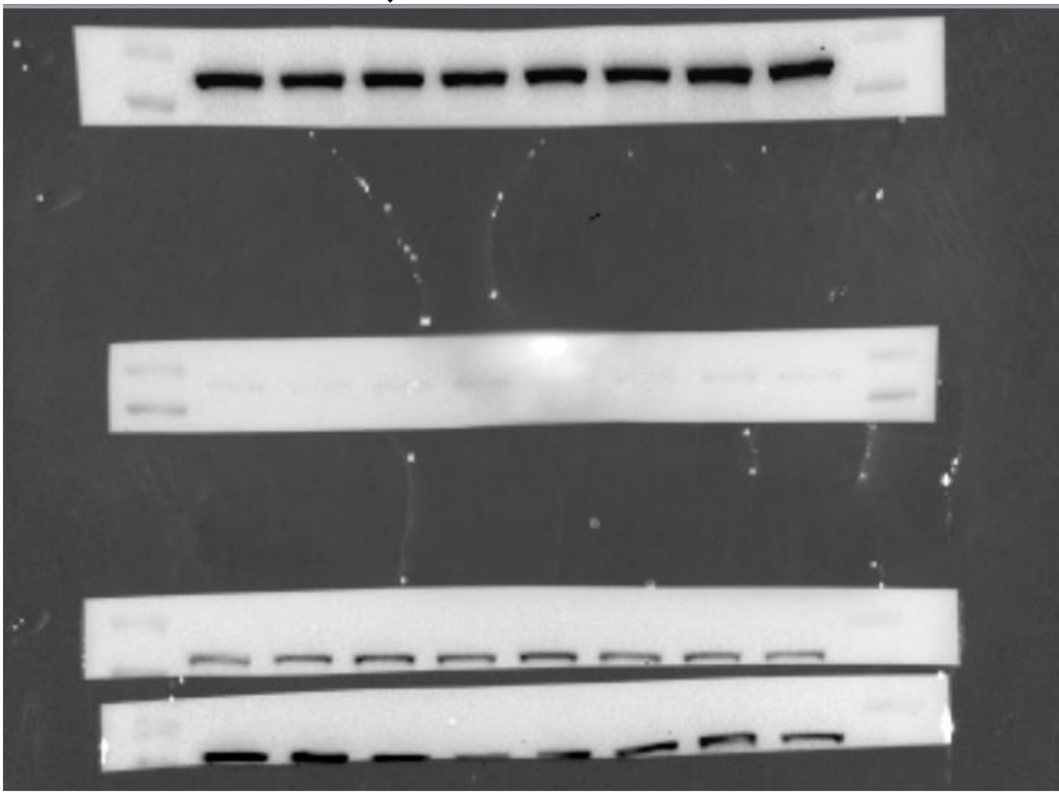

SW480

Original Western blot image for Figure 5-6

p-AMPK

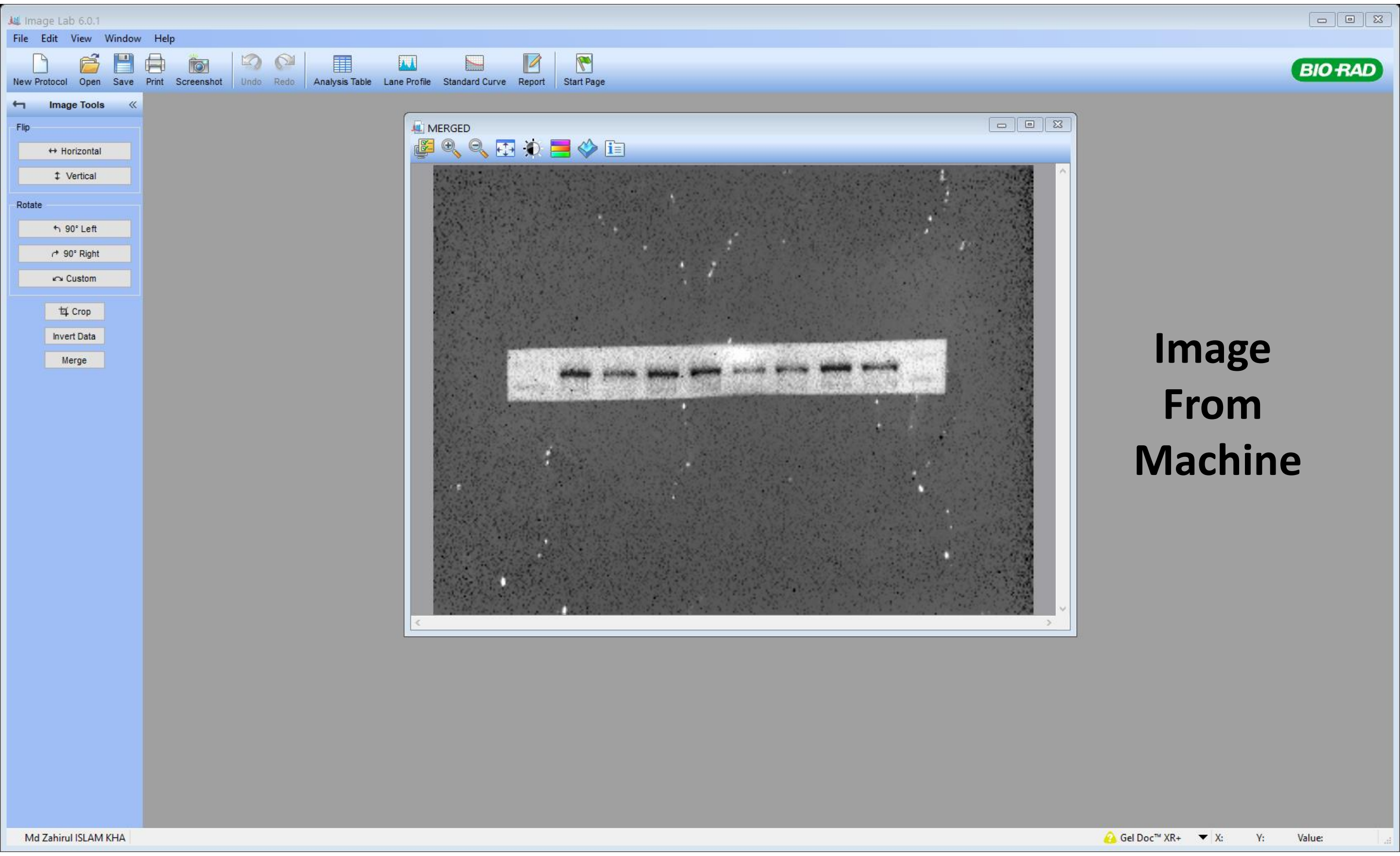

Cropped Image

p-AMPK-1

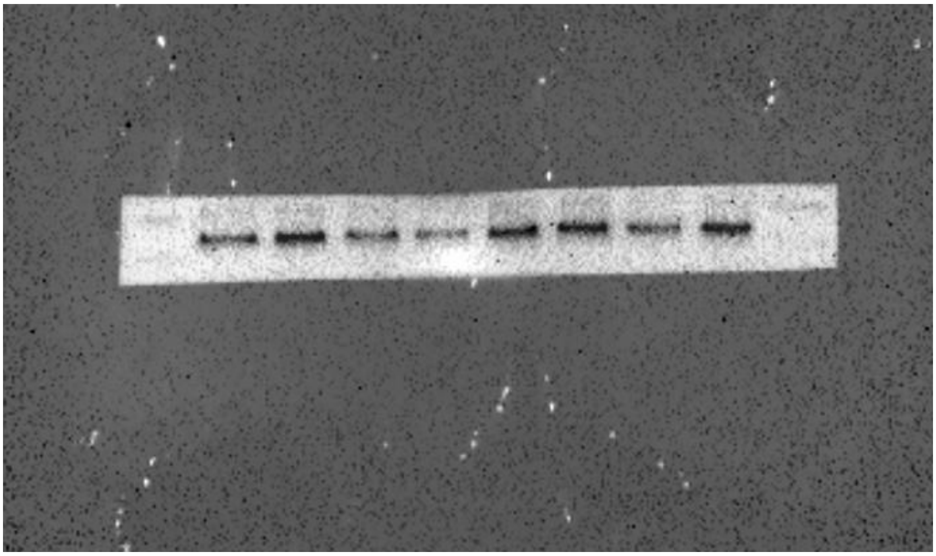

SW480

Original Western blot image for Figure 5-6

mTOR, p-mTOR, N-cadherin, E-cadherin

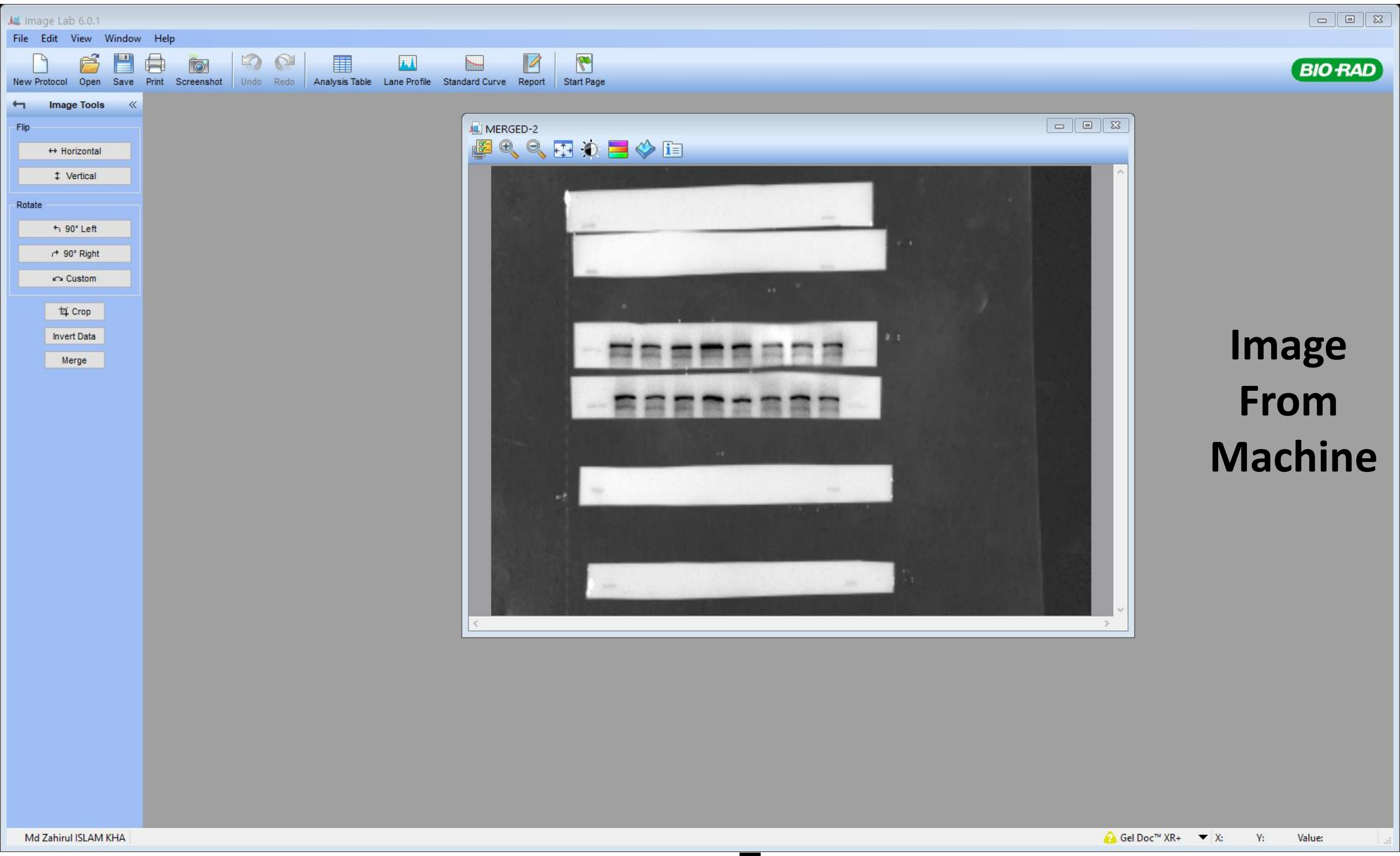

Image From Machine

Cropped Image

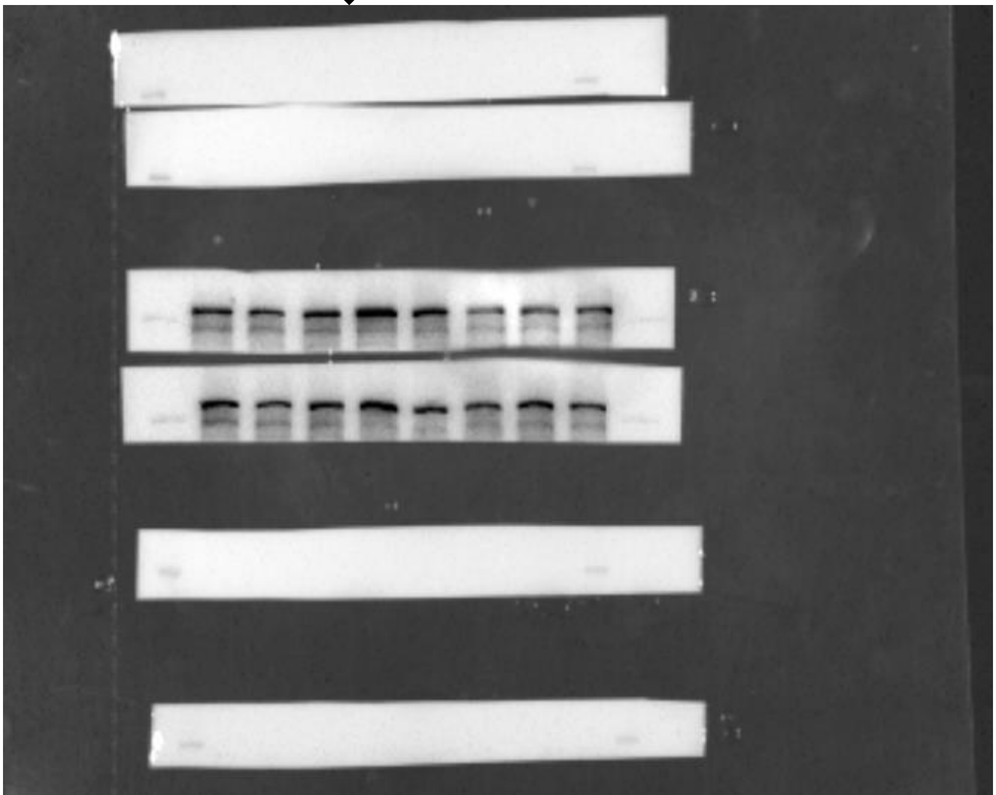

mTOR-1

mTOR-2

p-mTOR-1

N-cadherin-1

E-cadherin-1

SW480

Original Western blot image for Figure 5-6

p-AKT, p-AMPK

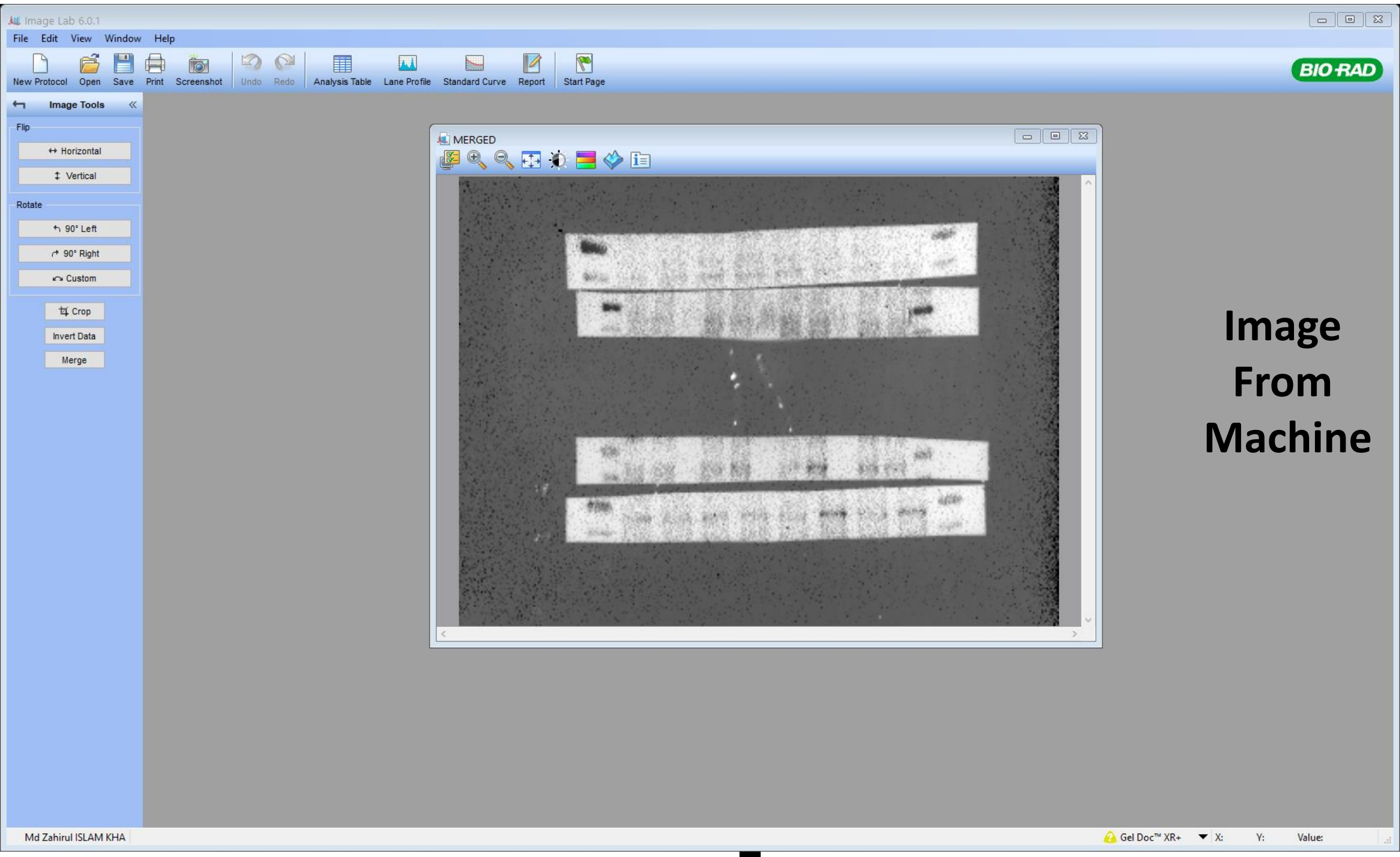

Image From Machine

Cropped Image

p-AKT-1

p-AKT-2

p-AMPK-2

p-AMPK-3

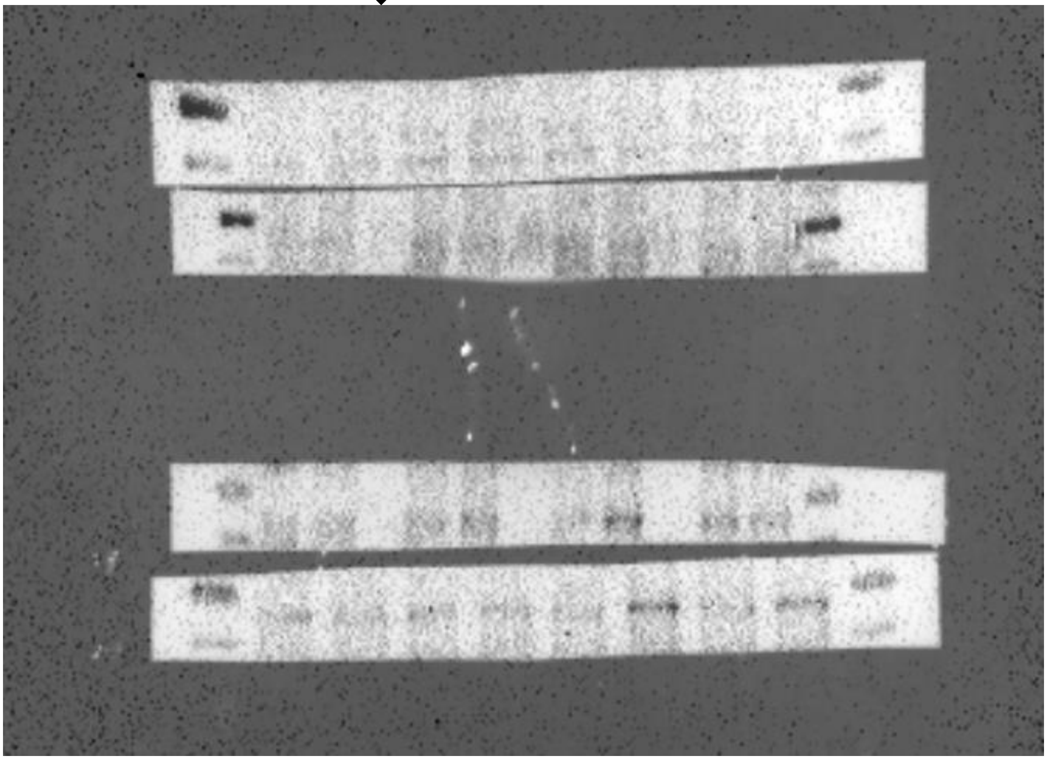

SW480

Original Western blot image for Figure 5-6

Snail

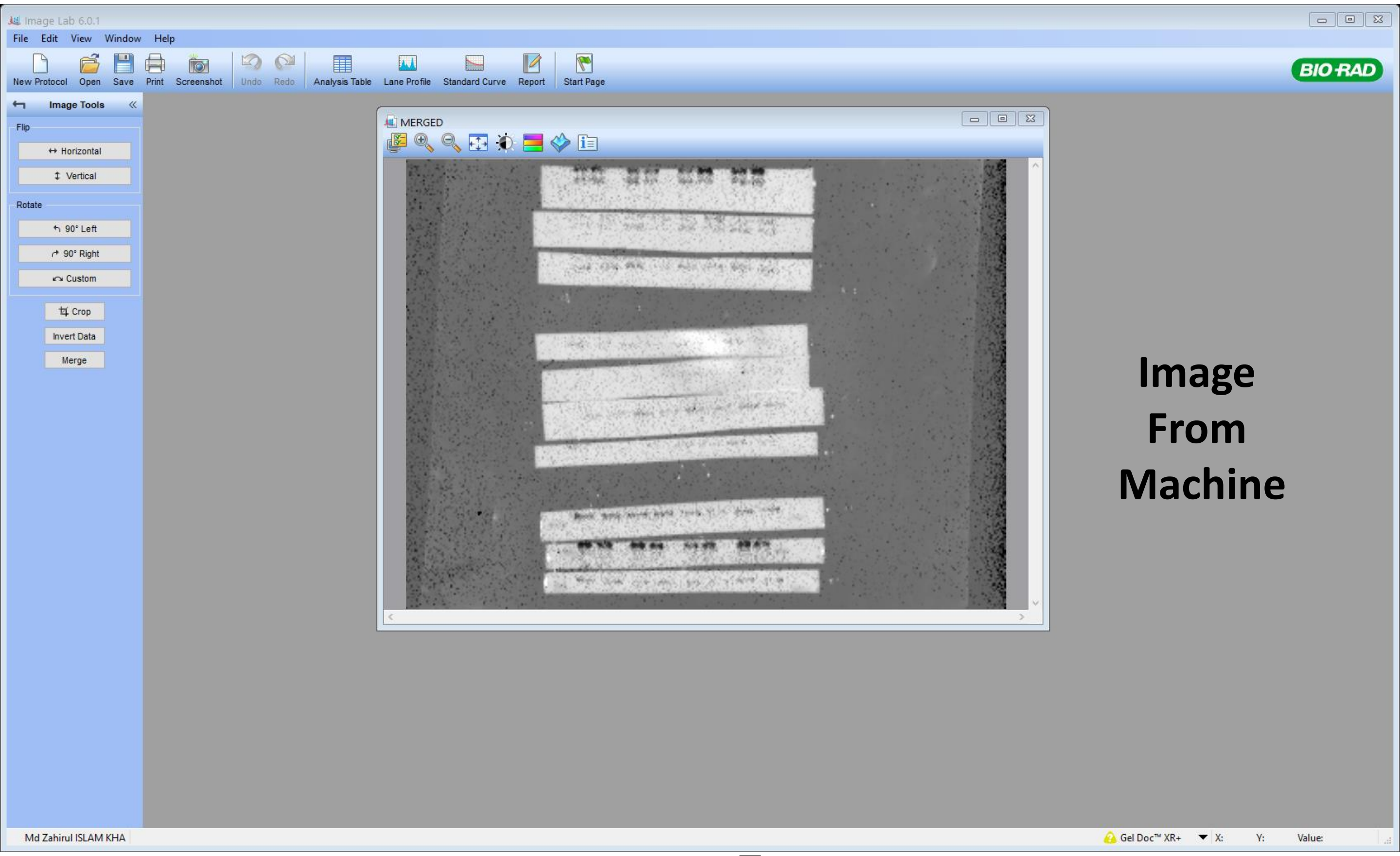

Image From Machine

Cropped Image

Snail-1
Snail-2
Snail-3

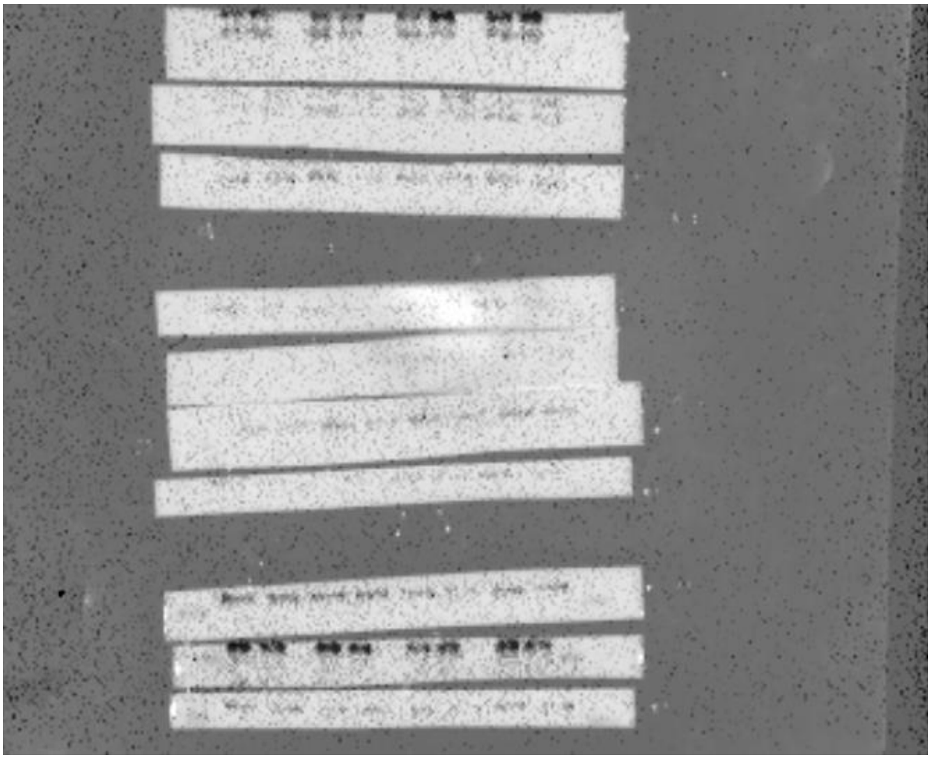

SW480

Original Western blot image for Figure 5-6

AKT, p-AKT

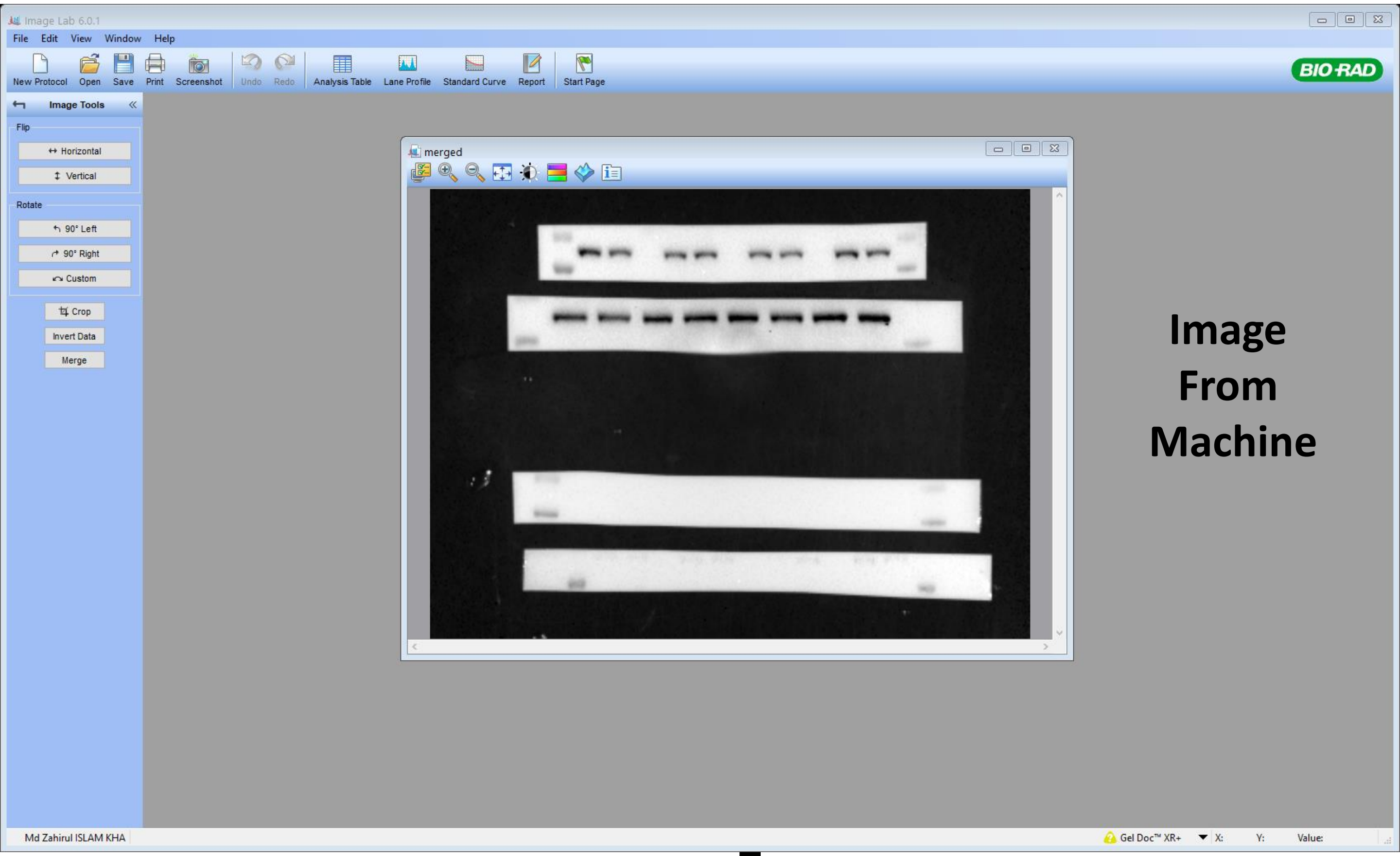

Image From Machine

Cropped Image

p-AKT-3

AKT-3

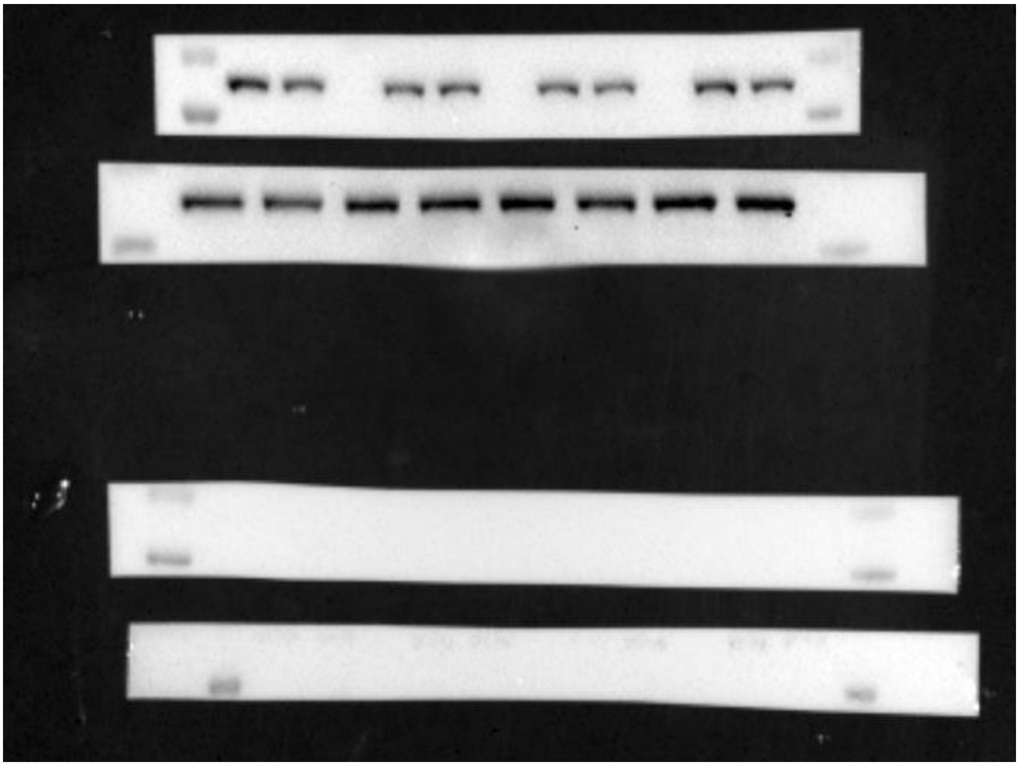

SW480

Original Western blot image for Figure 5-6

p-AMPK

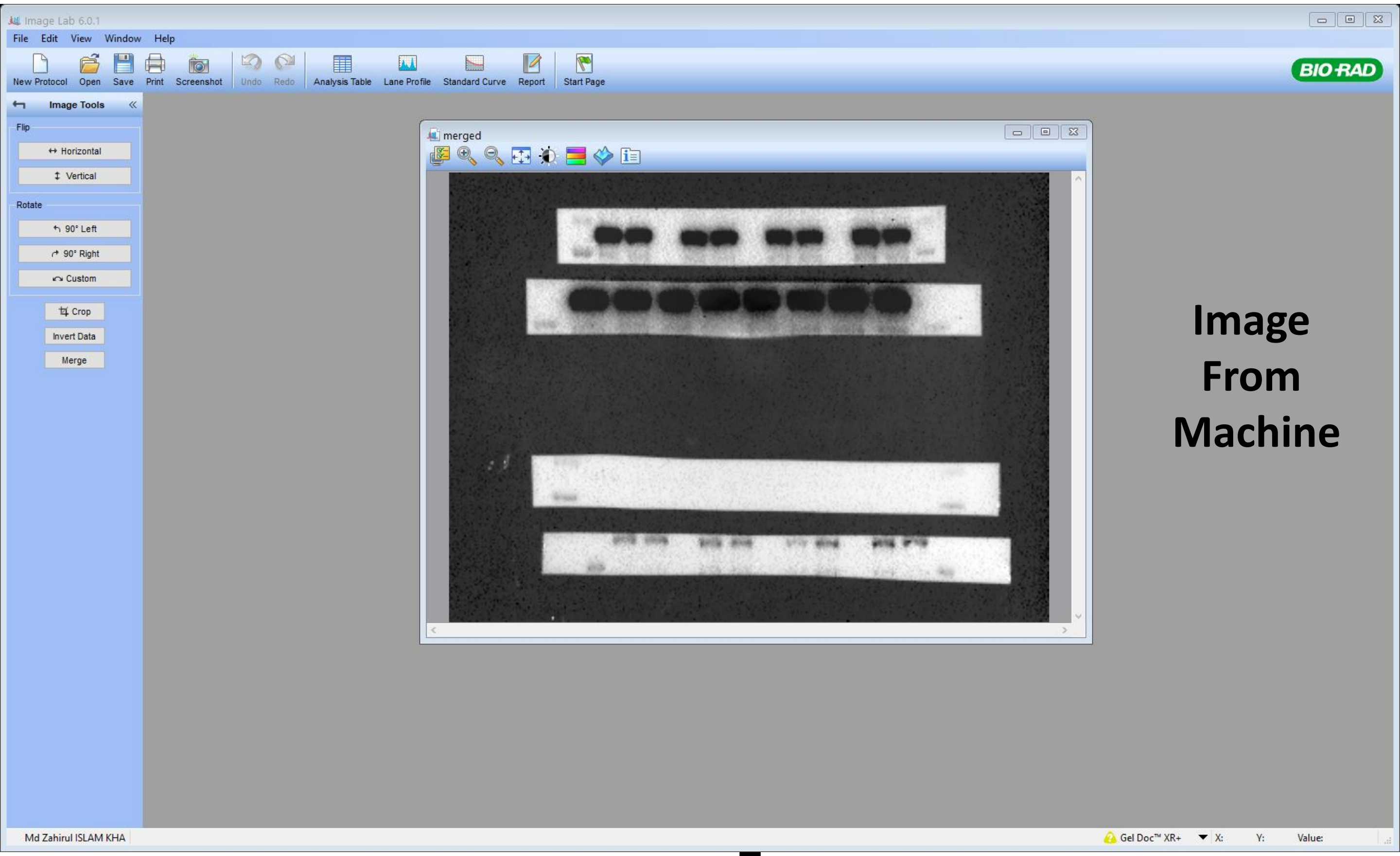

Image From Machine

Cropped Image

p-AMPK-3

p-AMPK-4

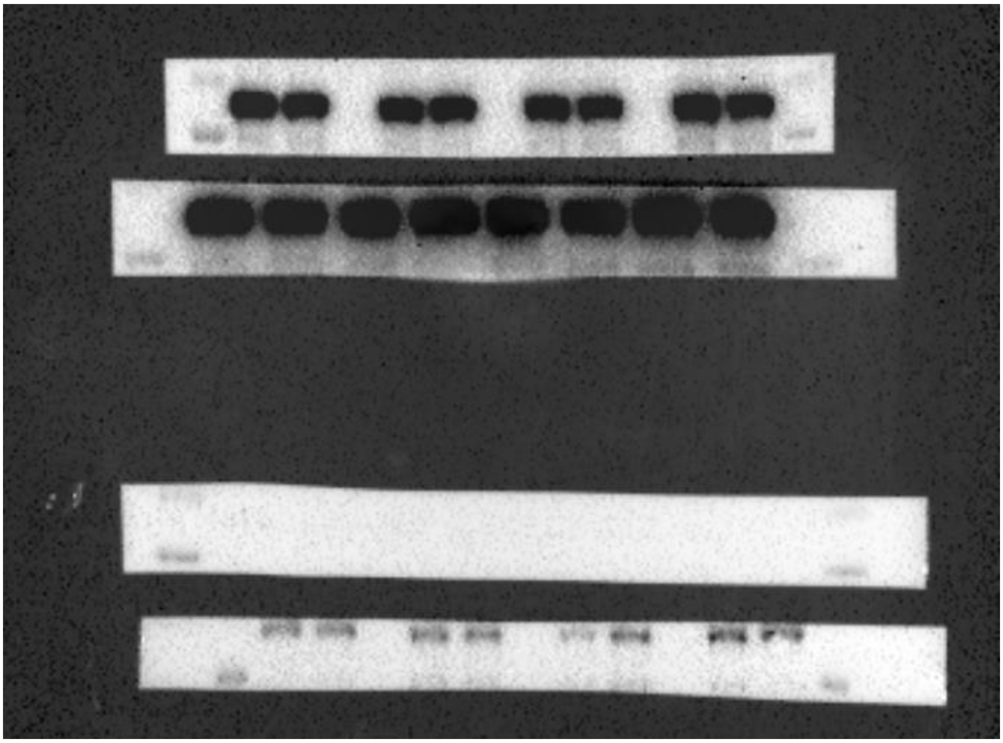

SW480

Original Western blot image for Figure 5-6

E-cadherin

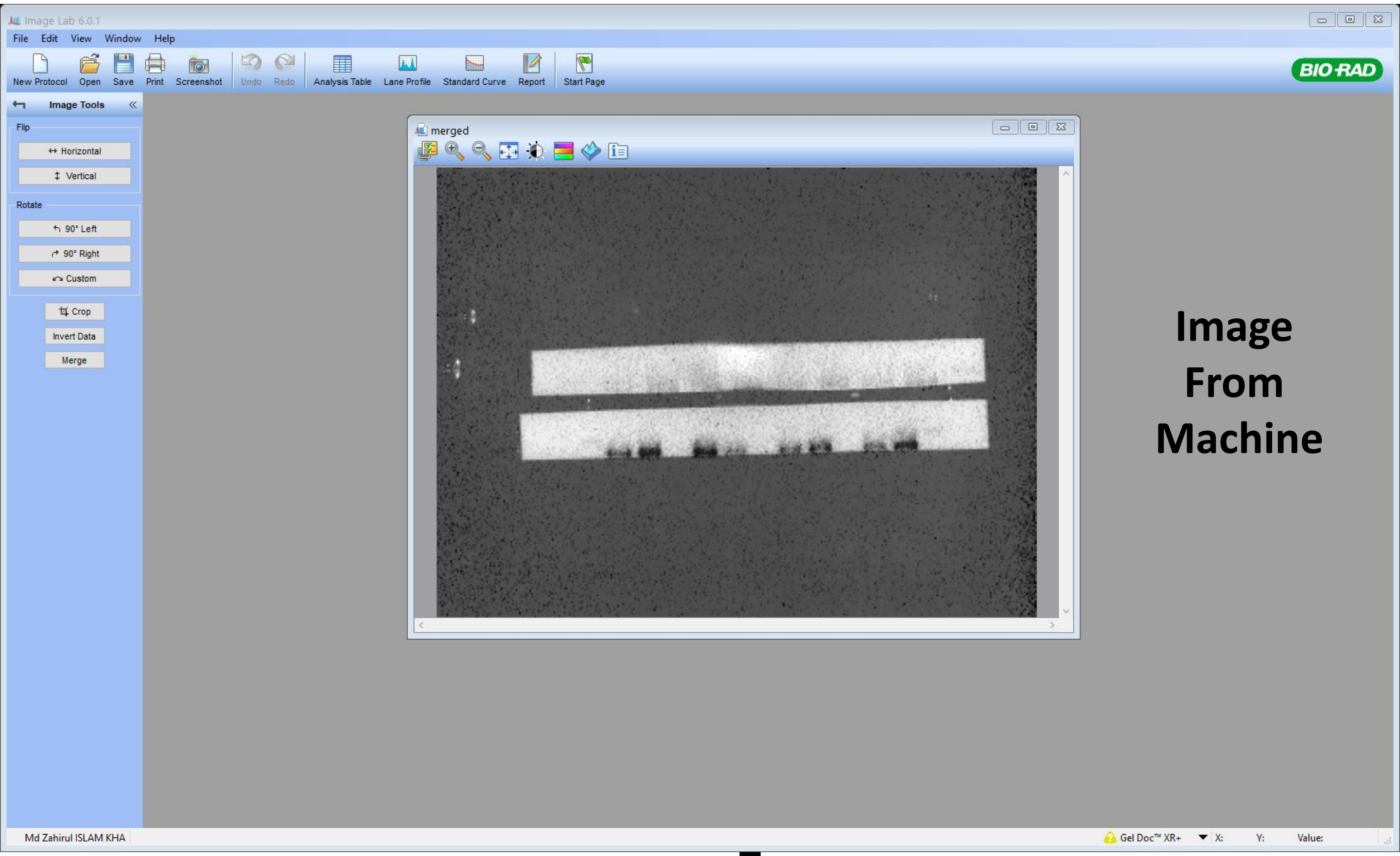

Image From Machine

Cropped Image

E-cadherine-2

E-cadherine-3

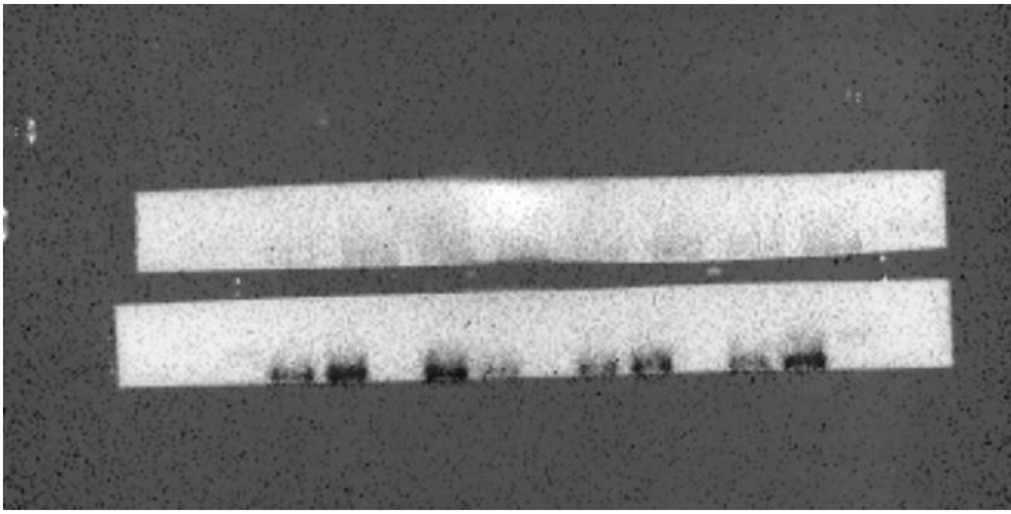

SW480

Original Western blot image for Figure 5-6

mTOR, p-mTOR

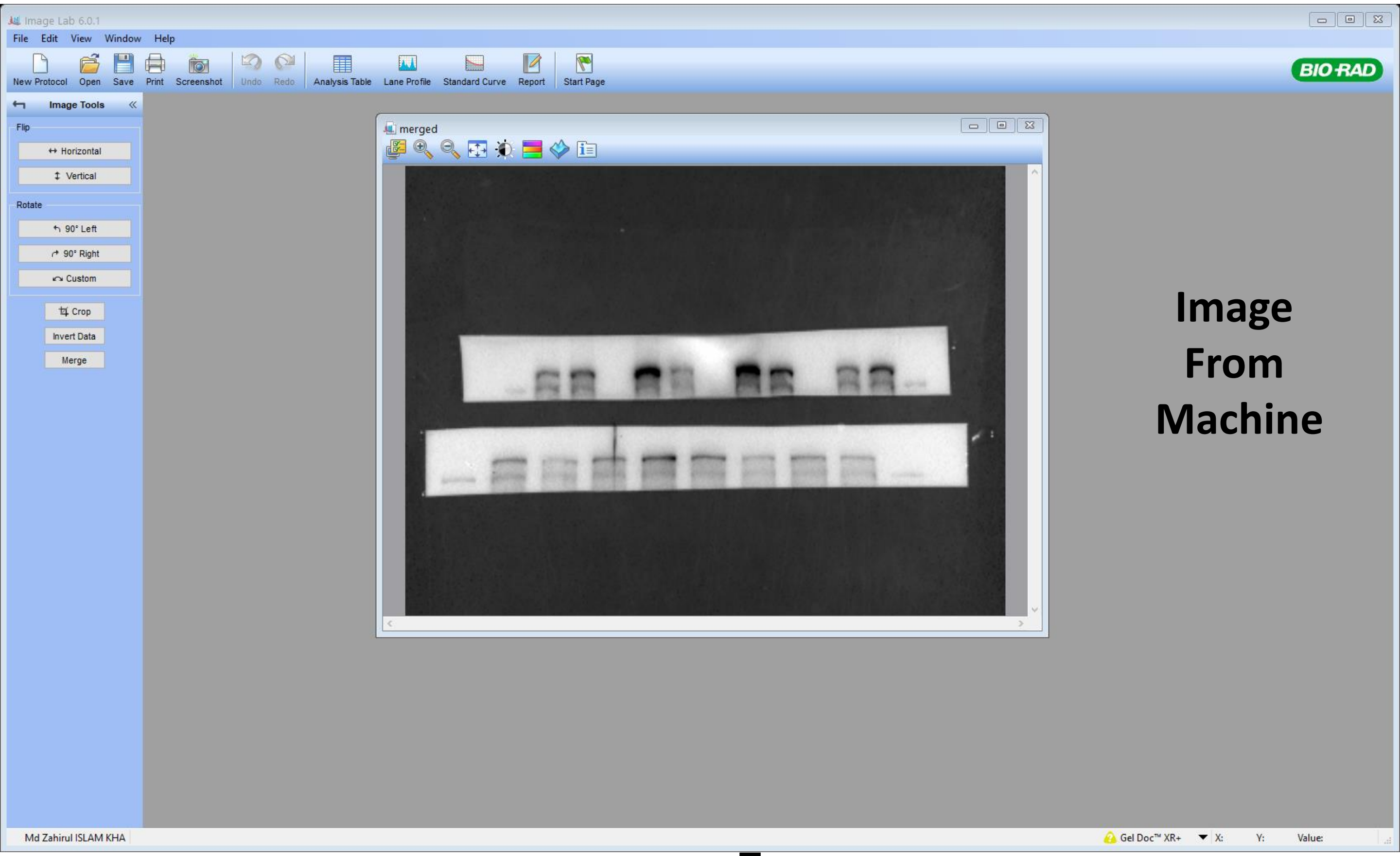

Cropped Image

p-mTOR-3

mTOR-3

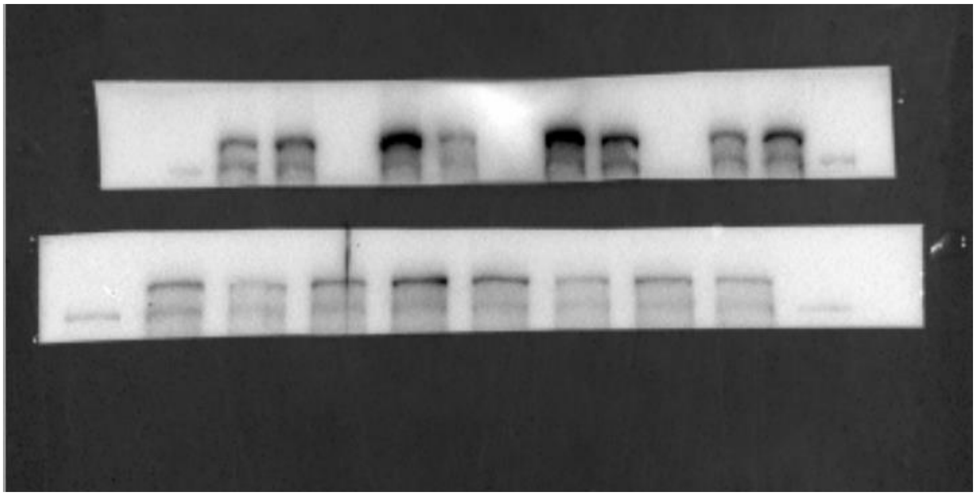

SW480

Original Western blot image for Figure 5-6

N-cadherin

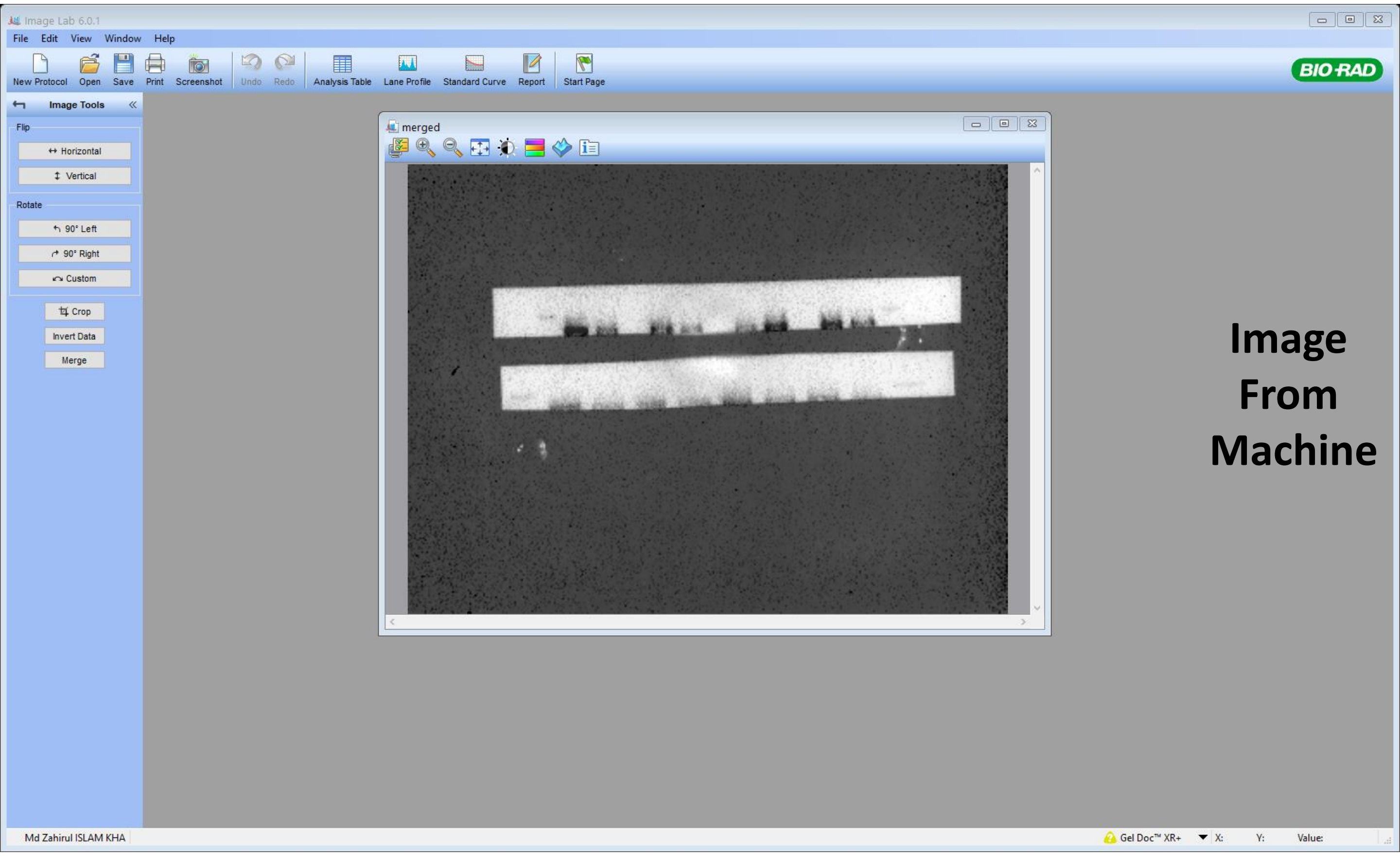

Image From Machine

Cropped Image

N-cadherin-2

N-cadherin-3

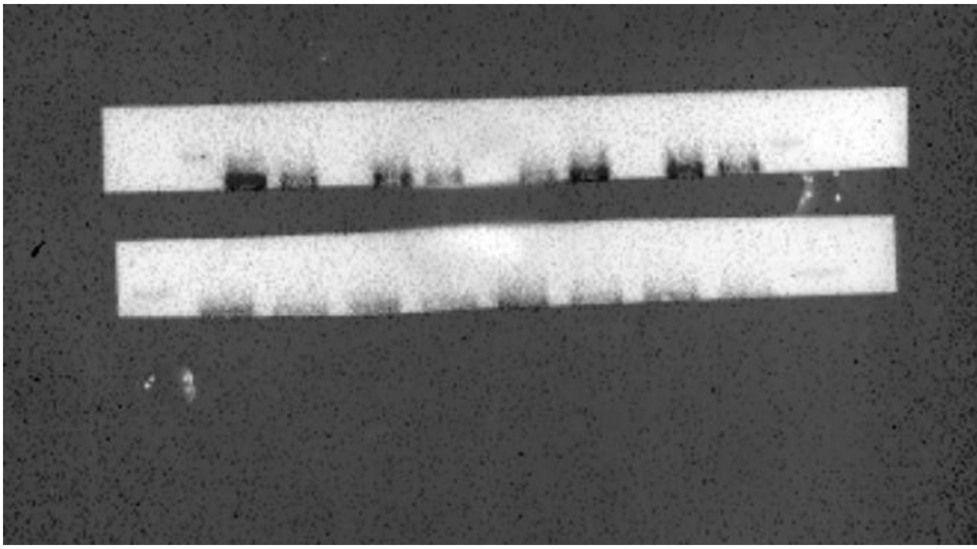

SW480

Original Western blot image for Figure 5-6

Snail, Sox-2

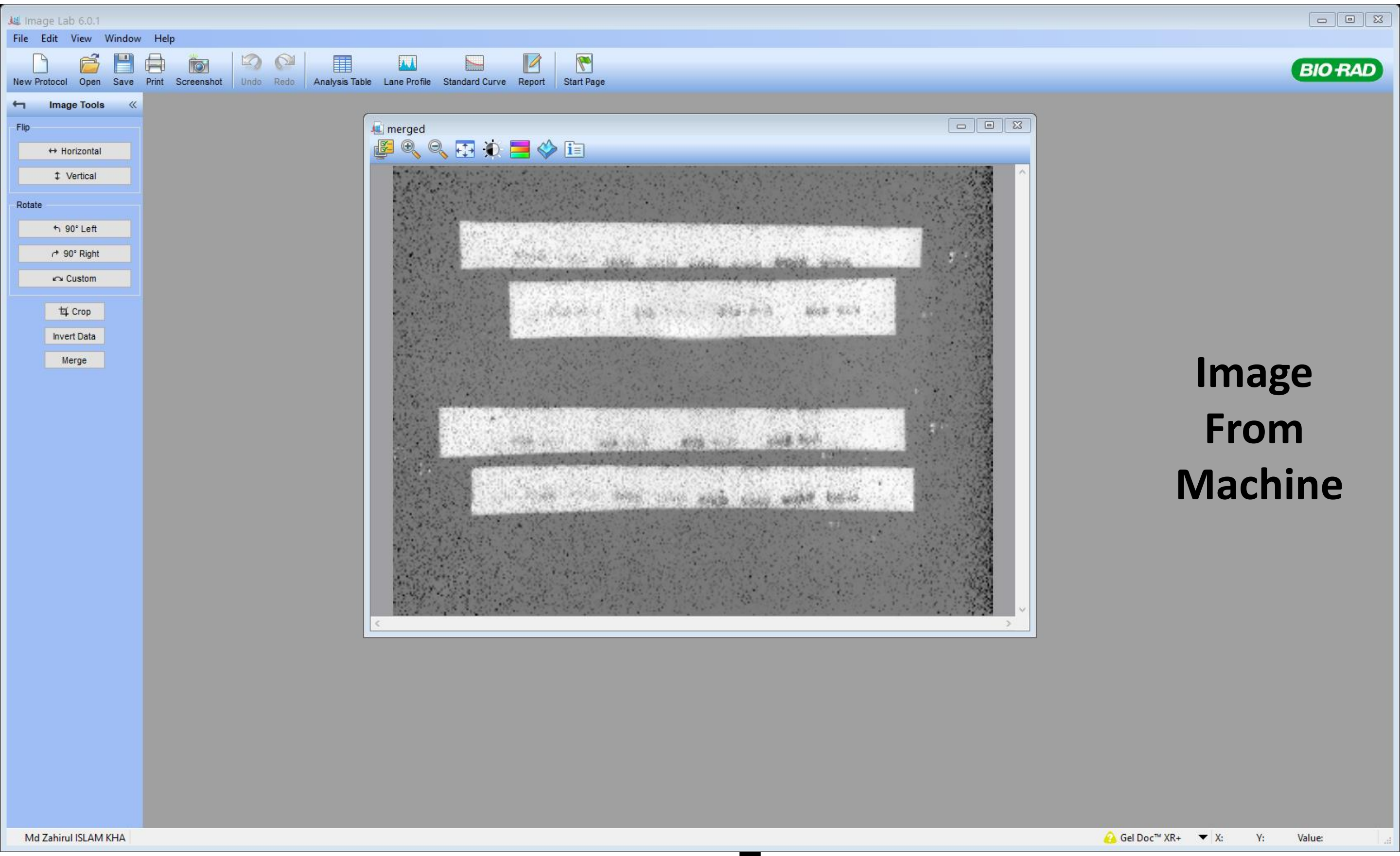

Cropped
Image

Snail-1

Snail-2

Sox-2-1

Sox-2-2

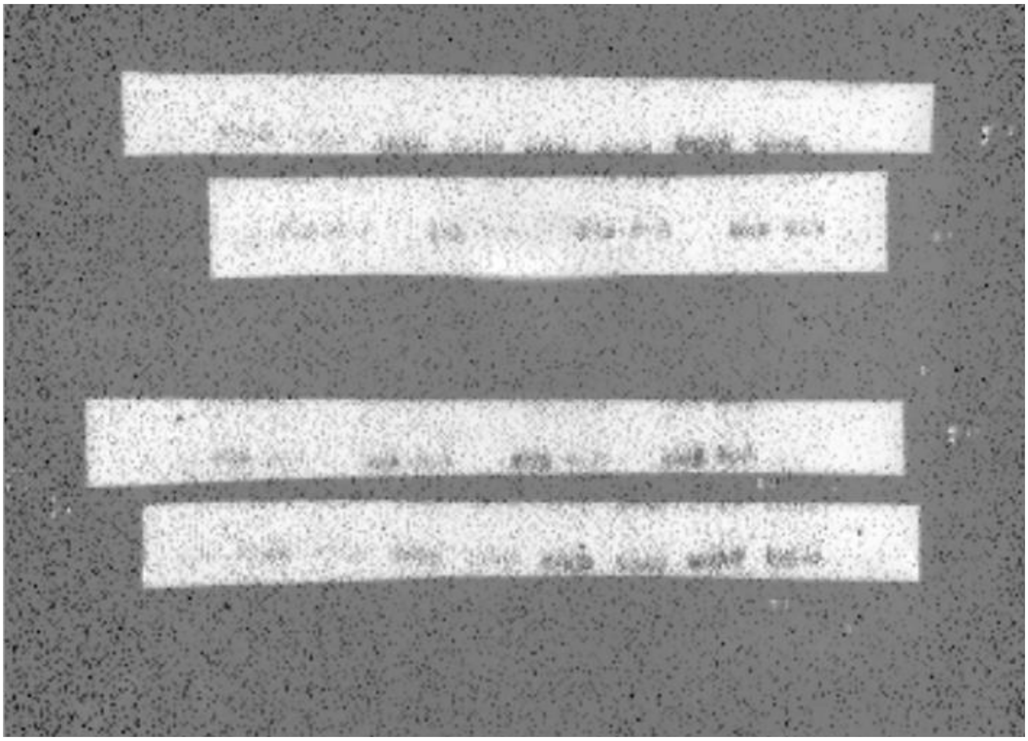

SW480

Original Western blot image for Figure 5-6

Snail, Sox-2

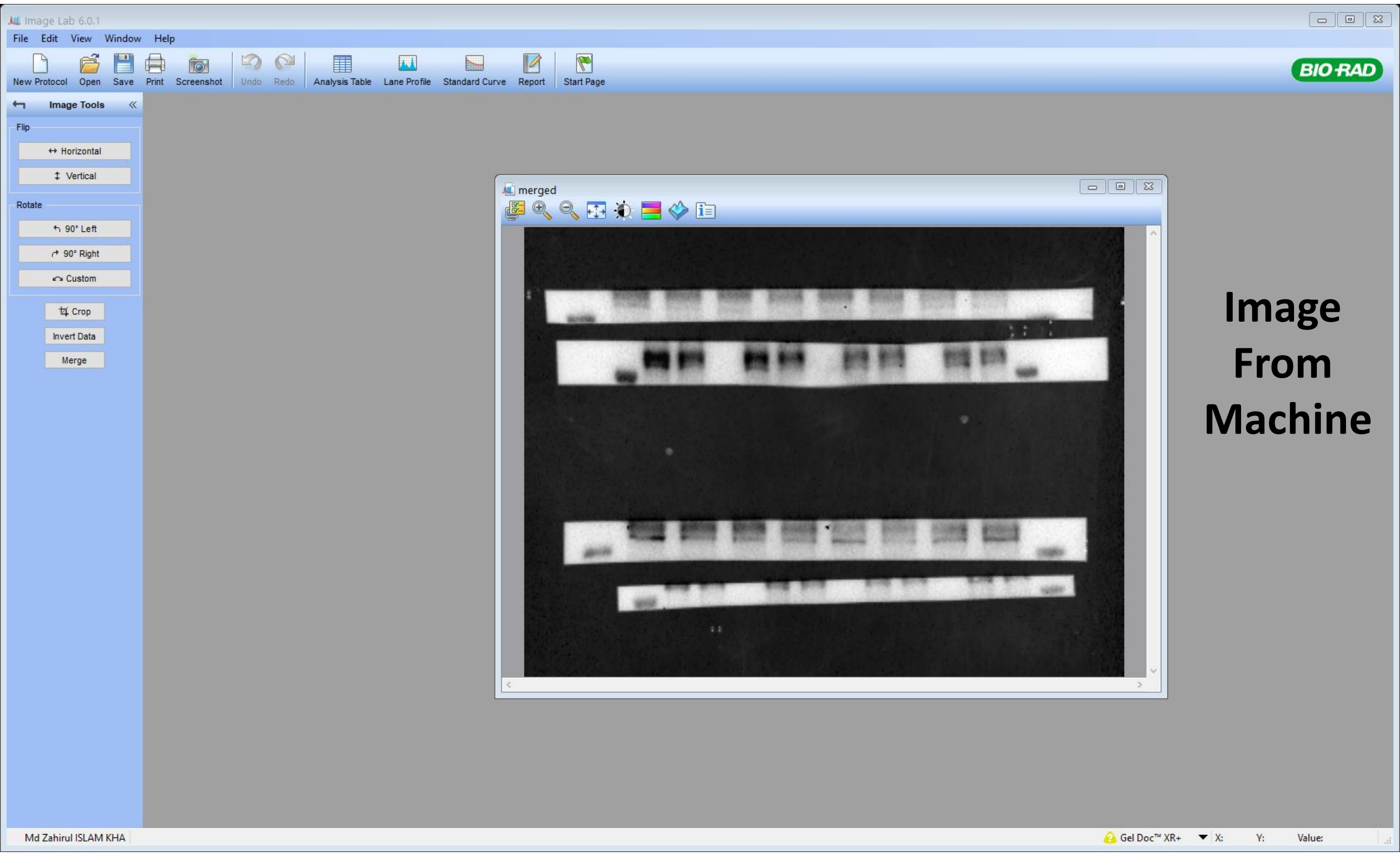

Cropped Image

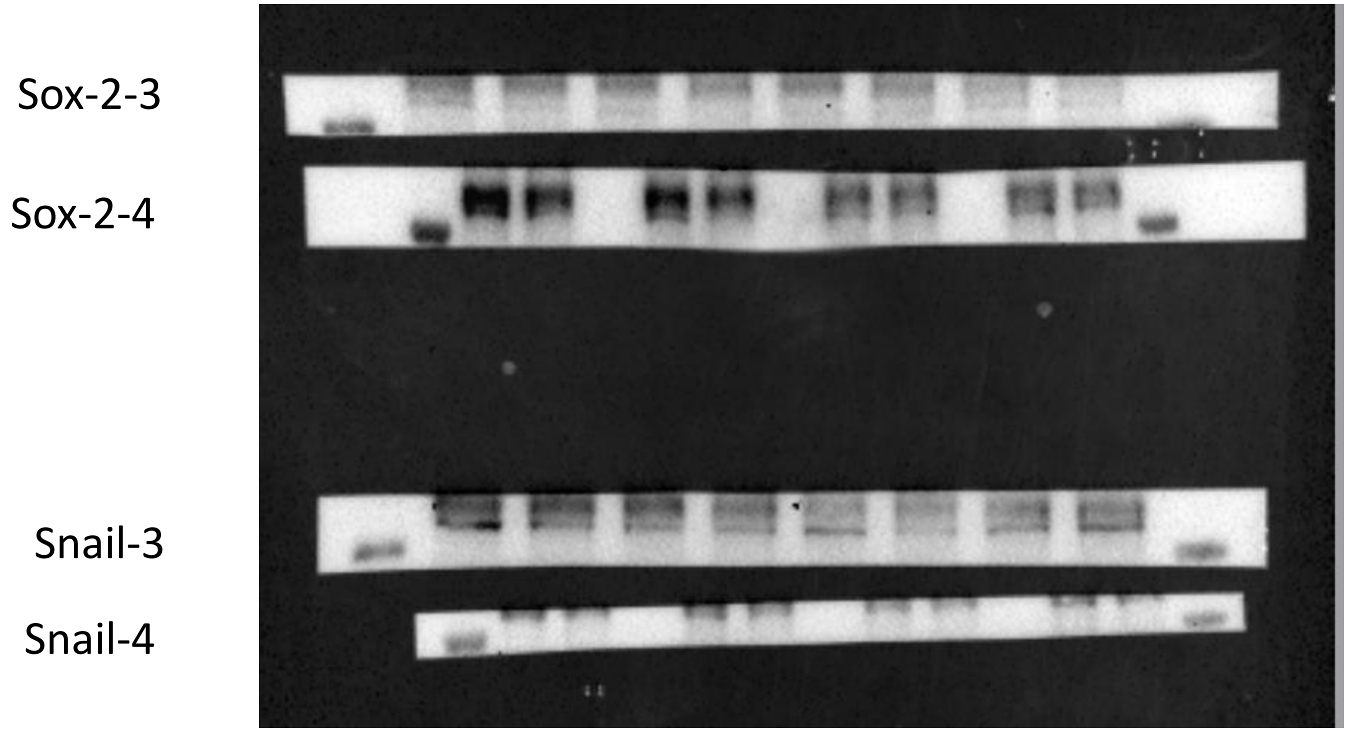

Supplement: Supplementary file 1 — Additional file 1. FASTA sequence of RAMS11 [file 12935_2021_2023_MOESM1_ESM.pdf]
