# Supplementary material for: RAMS11 promotes CRC through mTOR-dependent inhibition of autophagy, suppression of apoptosis, and promotion of epithelial-mesenchymal transition
Source: Cancer Cell Int. 2021 Jun 26;21:321. doi: 10.1186/s12935-021-02023-6 (PMC8236194; doi:10.1186/s12935-021-02023-6)
Supplement: Supplementary file 2 — Additional file 2. Original data of the results [file 12935_2021_2023_MOESM2_ESM.docx]

> RAMS11 hg19

AGAATGCCAAAGAGCAGCAGGATGGATCCAGCATCCTCTCCTGATAAAAG

AGGGCTAGAAGACGGGAGGCTCCGGGAAGTCTACTGGAGTCATGAAGACA

CTGAAAAGTGATGAATCCACATAACCATGACACTGGAAATGAAGTTTGAG

TGGCAGTCAGAATCTGGGAGGAAGCATTGCTAAGTGAAAATCTTATGGAG

CTTGACTAAAAATCCCTGTCAGGAACCGTCAAAAGCTGTGTCCCTGACAT

GAAAAATCTTGCTGGAAGTTGAGAGAGGTTTATGCCTACTCCGTGATCCG

GGAACACAAGACCTTTACCAACCAAAAAAGTGGATAGCTGTTCTTCTGCT

GTGAAGGTTAATAAAGAACGCCAGAAGTGCCAAGCAATTAACAACCCCAG

AAGCAACCCTTAACCAATGATTAAATAAAGTGGATGATTACATACCCAAG

CTCCTTCAACTCCCAGGGACATAATTCTGAGGGATGGAAAACAAACTGAA

ACTGGCTCAAGTGAATGCTCACTGGAAGGCTTACTGGAAAACTTACTGGA

AGGATGTGAGGACATGTTCGGGAATCTATTTGCAGAAAACATATTCAGCC

CTGTCCACCACAGCCAGCTGGCTGAAGAGCTCAAAAGGCAAGAAATCAGC

AAGAGAGAGAGATGAAGCATGAGAAATGAGCAAAAAACACCCAGCACATC

ATAATCTTGGACAGTTTAGCAGTACATGAAAATAGATGGTCCTCGCCCCA

AGGGACTGCAGTAACCCTGAATAAACAGGATGTCTCTCACTTTTAGCAGT

TCTTTCTGTGCTAGTATTGGGGAAATATATTTTTGGCTGCATGCAAAATG

GTAAAAGACATCTATTAAGAAAATGAAAACAATGCTTCTGTTTTAGACGA

AGCTTTTGAAGGTTTAAGGATCACCTATTTATTGACAAAATTGTTTCCGT

GGCTTAAAA
